# Noncoding RNA-nucleated heterochromatin spreading is intrinsically labile and requires accessory elements for epigenetic stability

**DOI:** 10.7554/eLife.32948

**Published:** 2018-07-18

**Authors:** R A Greenstein, Stephen K Jones, Eric C Spivey, James R Rybarski, Ilya J Finkelstein, Bassem Al-Sady

**Affiliations:** 1 Department of Microbiology & ImmunologyGeorge Williams Hooper Foundation, University of California San FranciscoSan FranciscoUnited States; 2 TETRAD graduate programUniversity of California San FranciscoSan FranciscoUnited States; 3 Department of Molecular BiosciencesThe University of Texas at AustinAustinUnited States; 4 Center for Systems and Synthetic BiologyThe University of Texas at AustinAustinUnited States; Institut CurieFrance; Harvard Medical SchoolUnited States

**Keywords:** heterochromatin spreading, multigenerational single cell tracking, epigenetic inheritance, cellular identity, epigenetics and environment, histone turnover, *S. pombe*

## Abstract

The heterochromatin spreading reaction is a central contributor to the formation of gene-repressive structures, which are re-established with high positional precision, or fidelity, following replication. How the spreading reaction contributes to this fidelity is not clear. To resolve the origins of stable inheritance of repression, we probed the intrinsic character of spreading events in fission yeast using a system that quantitatively describes the spreading reaction in live single cells. We show that spreading triggered by noncoding RNA-nucleated elements is stochastic, multimodal, and fluctuates dynamically across time. This lack of stability correlates with high histone turnover. At the mating type locus, this unstable behavior is restrained by an accessory *cis-*acting element *REIII*, which represses histone turnover. Further, *REIII* safeguards epigenetic memory against environmental perturbations. Our results suggest that the most prevalent type of spreading, driven by noncoding RNA-nucleators, is epigenetically unstable and requires collaboration with accessory elements to achieve high fidelity.

## Introduction

The formation of gene-repressive heterochromatin domains is critical for genome integrity and for the establishment and maintenance of cell identity. Most heterochromatin formation occurs by a sequence-indifferent spreading reaction that propagates heterochromatic marks, structural proteins, and associated effector proteins outwards from nucleation sites. The precise extent of the spreading reaction has critical heritable consequences for cell identity. For example, in early pluripotent precursors, pre-existing heterochromatin domains spread, sometimes over megabases, to repress specifiers of inappropriate fates. Importantly, the final extent of spreading from a locus depends on the lineage pathway, hence it varies across different precursors (Wen et al., 2009; Zhu et al., 2013) and has to be precise to achieve a stable cell fate and avoid disease (Ceol et al., 2011). Similarly, spreading also specifies cell type in yeasts, where the cell type is maintained by repressing the mating cassettes at the mating type loci (Ekwall et al., 1991; Rusche et al., 2003). Despite the centrality of the spreading reaction in shaping cell identity, its native and intrinsic cellular characteristics, as well as mechanisms for its inter-generational propagation, have remained opaque.

We have some understanding of how cells inherit silencing at nucleation sites, which constitute the DNA-sequence driven component of heterochromatin. Recent results in heterochromatin systems signaled by Histone 3 Lysine 9 and Lysine 27 methylation (H3K9me and H3K27me) indicate that several mechanisms act together to ensure intergenerational inheritance: continuous DNA-mediated recruitment of the histone methylase (Audergon et al., 2015; Jia et al., 2004; Laprell et al., 2017; Ragunathan et al., 2015; Wang and Moazed, 2017), low histone turnover (Aygün et al., 2013; Taneja et al., 2017), as well as the positive ‘read-write’ feedback loop for histone methylases (Al-Sady et al., 2013; Zhang et al., 2008). Additionally, studies suggest that either the histone mark (Gaydos et al., 2014) or the histone methylases (Petruk et al., 2012) can persist trans-generationally.

These insights concerning nucleation sites do not necessarily account for how regions of heterochromatin distal to these sites are maintained. Unlike nucleation, which depends on DNA-based enzyme recruitment (Bayne et al., 2010; Verdel et al., 2004), spreading depends on the ability of the system to propagate along the chromosome, independent of the underlying DNA sequence. Such propagation requires the ‘read-write’ positive feedback function of the system (Al-Sady et al., 2013; Margueron et al., 2009; Müller et al., 2016; Noma et al., 2004; Zhang et al., 2008).

To determine how the spreading reaction acts in the maintenance of cell fate, it is central to understand the native behavior of two interconnected but separable phases of spreading: The initial spreading event, and the propagation of the states formed by this initial event through cell divisions. There is evidence that the initial spreading, at least in contexts outside the native chromosomal position, is stochastic, that is only some nucleation events result in a spreading event. This was first demonstrated by observing position effect variegation (PEV) in flies (Elgin and Reuter, 2013; Muller, 1930). Such stochastic behavior would have to be mitigated across cells to achieve a coherent specification outcome.

Intergenerational propagation of spreading is straightforward to conceptualize when epigenetic information is strongly reinforced, but more challenging in situations where modified nucleosomes are less likely to persist. This is the case for H3K9me-signaled heterochromatin in the fission yeast system, which lacks DNA methylation that can reinforce the epigenetic state. Persistence of the modified state is opposed by an anti-silencing protein Epe1 (Ayoub et al., 2003; Zofall and Grewal, 2006), which acts by antagonizing retention of H3K9me histones (Aygün et al., 2013; Ragunathan et al., 2015), and passage through S-phase, which significantly weakens heterochromatin domains (Chen et al., 2008). For fission yeast, there is evidence in favor of both high fidelity and stochastic propagation of the state formed by spreading. In support of a high fidelity model, theoretical work suggests that heterochromatin can display fundamentally bistable behavior, indicating that the ‘ON’ and ‘OFF’ states are intrinsically highly stable (Dodd et al., 2007). Similar bistable behavior has also been experimentally observed in plants (Angel et al., 2011, 2015). Conversely, the telomere position effect (TPE) observed in budding and fission yeast supports a model where intergenerational inheritance is fundamentally stochastic. In TPE the heterochromatic state is switched at high frequencies in the inheriting generations (Gottschling et al., 1990; Nimmo et al., 1994).

To distinguish whether spreading shapes and enables epigenetic maintenance of a cell identity locus via either of those modes, or combinations thereof, we focused on one of the most well understood heterochromatin loci, the fission yeast MAT locus, as a model. This locus remains tightly repressed to avoid simultaneous expression of both mating cassettes (Ekwall et al., 1991; Noma et al., 2001). The MAT locus contains two *cis* elements that directly recruit H3K9me. (1) *cenH,* which is related to the *dg* and *dh* repeats at the pericentromere and *tlh2* at the subtelomere (Grewal and Klar, 1997; Hansen et al., 2006). These sequences nucleate H3K9me by at least two pathways, which depend on transcription of noncoding RNAs (ncRNAs): the RNAi pathway (Hall et al., 2002; Volpe et al., 2002), and at least one separate pathway dependent on nascent RNA polymerase II transcripts, which requires the budding yeast Nrd1 homology Seb1 (Marina et al., 2013) (collectively ‘ncRNA-nucleation’). Separately and unique to the MAT locus, (2) a region downstream of *cenH* including the *REIII* element, which recruits the H3K9 histone methylase, HP1 proteins and histone deacetylases (HDACs). This is dependent on *REIII-*bound transcription factors (Jia et al., 2004; Kim et al., 2004; Yamada et al., 2005), but is independent of RNA processes. Heterochromatin formation within the MAT locus is confined by boundary elements (Noma et al., 2001, 2006).

In this work, we probe heterochromatin spreading nucleated both at the MAT locus as well as ectopically in the genome with a ‘heterochromatin spreading sensor’ (HSS), which enables quantitative examination of spreading separately from nucleation in single *S. pombe* cells. Using the HSS, we show that ncRNA-dependent elements trigger epigenetically unstable spreading that is stabilized by an accessory RNA-independent *cis-*ating element. Both elements collaborate to form a high fidelity domain. The strategy we uncover has important implications for how heterochromatin spreading achieves and maintains ‘epigenetic’ character and can safeguard cell identity against environmental perturbations.

## Results

### A single-cell heterochromatin spreading sensor (HSS) controls for nucleation and cellular noise

**Figure 1.**
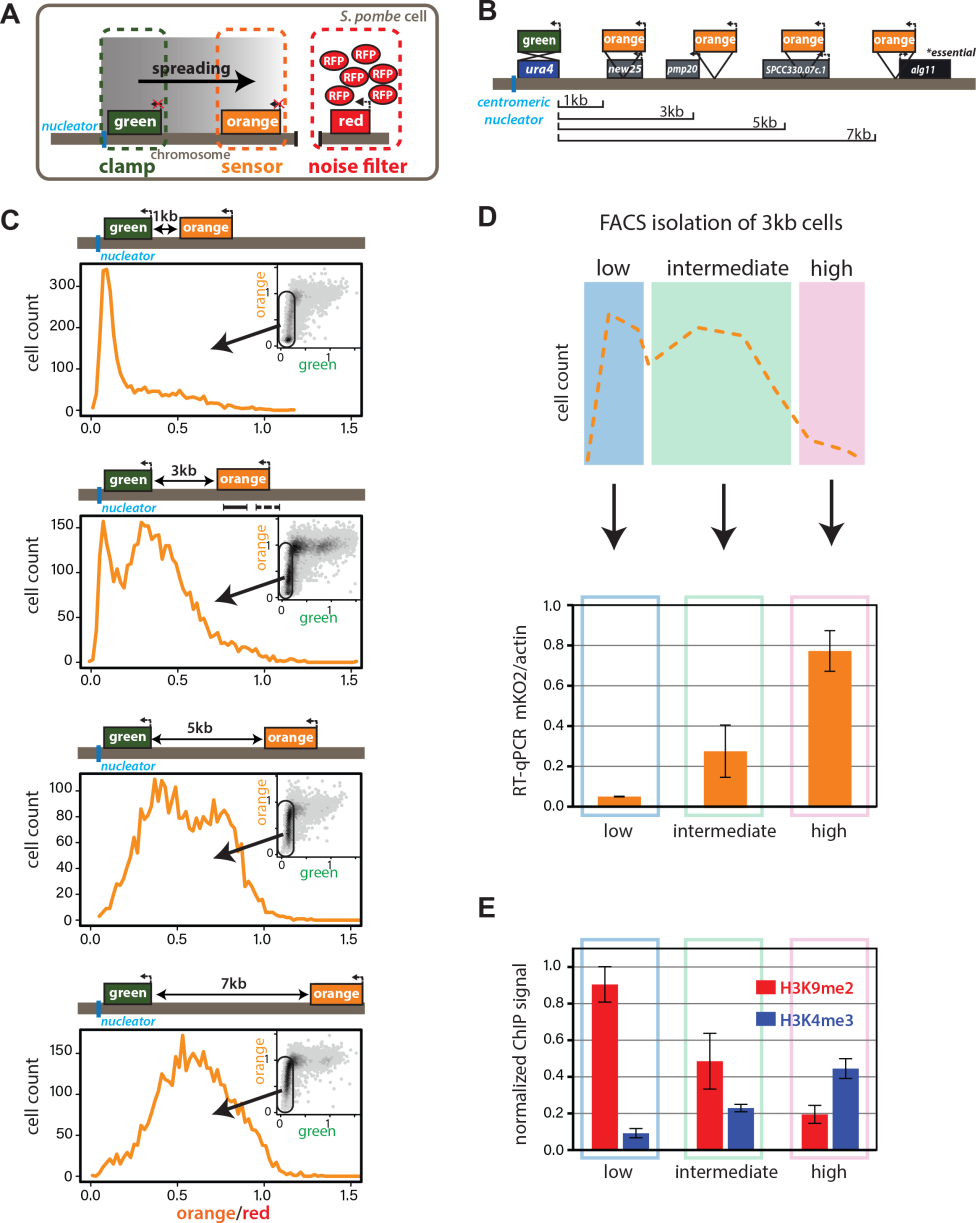
Heterochromatin spreading from ncRNA-nucleated elements is stochastic and produces intermediate states. (**A**) Overview of heterochromatin spreading sensor. Three transcriptionally encoded fluorescent proteins are inserted in the genome: The ‘clamp’ site enables isolation of successful nucleation events, the ‘sensor’ reports on spreading events and the ‘noise filter’ normalizes for cell-to-cell noise. (**B**) Overview of the *ura4::dh*HSS^1-7kb^ strains. Genes downstream of the ‘green’ nucleation color are annotated. The *alg11 *gene is essential. (**C**) Spreading from *ura4::dh* visualized by the HSS with ‘orange’ inserted at different distances shown in (**B**). The ‘red’-normalized ‘orange’ fluorescence distribution of ‘green”^OFF^ cells plotted on a histogram. Inset: 2D-density hexbin plot showing red-normalized ‘green’ and ‘orange’ fluorescence within the size gate, with no ‘green’ or ‘orange’ filtering. The ‘green'^OFF^ population is schematically circled. The fluorescence values are normalized to = 1 for the *Δclr4* derivate of each strain. (**D**) TOP: cartoon overview of the FACS experiment for D. and E. ‘green'^OFF^ cells collected from the *ura4::dh*HSS^3kb^ were separated in three populations (‘Low’, ‘Intermediate’ and ‘High’) as shown schematically based on the ‘orange’ fluorescence. BOTTOM: ‘orange’ RT-qPCR signal for the indicated populations. The y-axis is scaled to = 1 based on the ‘orange’ signal in *Δclr4.* Error bars indicate standard deviation of two replicate RNA isolations. (**E**) ChIP for H3K9me2 and H3K4me3 in the same populations as (**D**). Each ChIP is normalized over input and scaled to = 1 for a positive control locus (*dh* repeat for H3K9me2 and *act1* promoter for H3K4me3). Error bars indicate standard deviation of two technical ChIP replicates. Primer pairs for RT-qPCR and ChIP are indicated by solid and dashed line, respectively, in the C. *ura4::dh*HSS^3kb^ diagram.

To assess the intrinsic behavior of heterochromatin spreading and what shapes its precise re-establishment with respect to position and extent of repression (‘fidelity’), we employed transcriptionally encoded fluorescent reporters to read silencing by heterochromatin at a given locus, as previously reported. Several critical improvements over prior systems enable documentation of the spreading reaction at high sensitivity (Bintu et al., 2016; Hathaway et al., 2012; Obersriebnig et al., 2016; Osborne et al., 2009; Xu et al., 2006). First, our system has high signal to noise and minimized delay from epigenetic changes to fluorescent output. We accomplish this using the weak, well-characterized *ade6* gene promoter (*ade6p*) (Allshire et al., 1994; Kagansky et al., 2009) to drive production of bright, fast-folding fluorescent proteins (XFPs) (Al-Sady et al., 2016). Second, our system provides separate sensors for nucleation, spreading, and cellular noise. We used *ade6p-*driven recoded super-folder GFP (Pédelacq et al., 2006) (‘green’) and monomeric Kusabira Orange (Sakaue-Sawano et al., 2008) (‘orange’) to report on nucleation and spreading, respectively (Figure 1A). A third XFP, an *ade6p*-driven triple fusion of E2Crimson (Strack et al., 2009) (‘red’, noise filter), is fully uncoupled from heterochromatin and inserted in a euchromatic locus. Here it reports on intrinsic or extrinsic noise that arises from cell-to-cell variation in the content of specific and general transcription factors and also translational efficiency (Figure 1A). To validate this reporter system, we characterized the non-heterochromatic state, via null mutation of *clr4* (*Δclr4*), encoding the only *S. pombe* H3K9 methyltransferase. We show that in the absence of heterochromatin, expression of the noise reporter (‘red’) correlates well with that of reporters for both nucleation (‘green’) and spreading (‘orange’) (Figure 1—figure supplement 1A,B), especially when all cells in the population are considered without applying a size gate (Figure 1—figure supplement 1B, ρ ~0.83–0.93). This analysis mode is required when cell number is limiting. When a smaller subset is considered where all the cells are of similar size and stage of the cell cycle, the correlation still provides useful noise filtering (Figure 1—figure supplement 1A), which becomes evident when the normalization is applied to *clr4+* cells that fall in the size gate (Figure 1—figure supplement 1C). Thus, cellular noise is mitigated by dividing the signals from the proximal ‘green’ and distal ‘orange’ heterochromatic reporters by the signal of the ‘red’, euchromatic reporter (‘green’/‘red’; ‘orange’/‘red’). Together, these elements constitute our heterochromatin spreading sensor (HSS) (Figure 1A).

**Figure 1—figure supplement 1.**
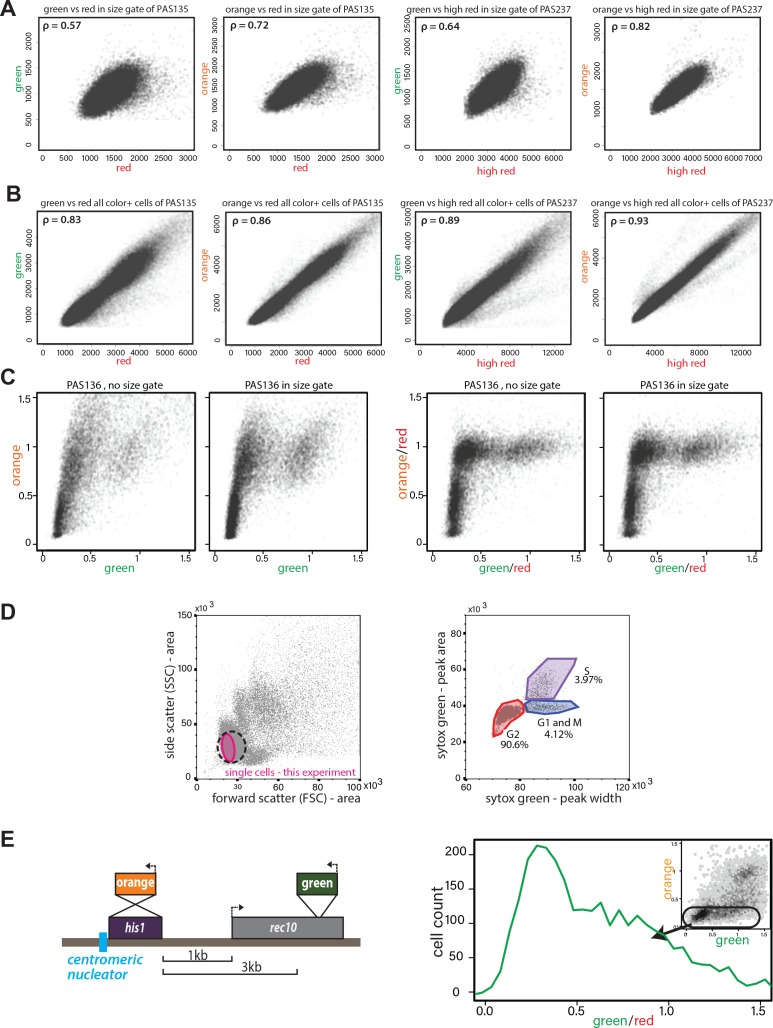
Validation of ectopic heterochromatin spreading sensor. (**A**) Correlation of *ade6p:SFGFP* or *ade6p:mKO2* with *ade6p:3XE2C* (Red) or *act1p:1XE2C* (High Red) in *Δclr4* HSS size-gated cells. LEFT: Plots of green and orange vs. red channel signals of size-gated PAS 135 (*Δclr4*, ‘red’). RIGHT: Plots of green and orange vs. red channel signals of size-gated PAS 237 (*Δclr4*, ‘high-red’). The Pearson correlation between ‘green’ and ‘red’/‘high-red’ or ‘orange’ and ‘red’/‘high-red’ is shown. (**B**) Correlation of *ade6p:SFGFP* or *ade6p:mKO2* with *ade6p:3XE2C* (Red) or *act1p:1XE2C* (High Red) in *Δclr4* HSS in cells without size gate. Plots and Pearson correlation as above. (**C**) Effect of red-normalization on distribution of *clr4+* HSS cells. Plots of green and orange vs. red channel signals of PAS 136, which contains the ectopic HSS (Figure 1C). LEFT: effect of using only size gate, without red normalization. RIGHT: effect of red-normalization with and without additional size gate. The distribution of cells is tightened by red-normalization. (**D**) Cell cycle stage of HSS and wild-type cells by flow cytometry. Wild-type cells (PM03, see strain table) were fixed, stained with Sytox green DNA stain, and analyzed by flow cytometry. LEFT: side vs. forward scatter plot. Dotted line: The approximate size gate encompassing all experiments reported. Pink area: cells analyzed in the experiment shown. RIGHT: Plot of area vs. width parameter for the Sytox green channel, gates are drawn to denote cell cycle phases, G2 (red), G1 and M (Blue), S (purple) as described (Knutsen et al., 2011). (**E**) Stochastic spreading and intermediate states produced by ncRNA-driven nucleators are replicated at a second ectopic site. LEFT: Overview of the *his1::dh*HSS^3kb^. The colors are reversed relative to the *ura4::dh*HSS^1-7kb^ with ‘orange’ as the ‘nucleation clamp’ and ‘green’ as the ‘sensor’. ‘Orange’ replaces the *his1* gene and ‘green’ is located 3 kb downstream within the *rec10* open-reading frame. RIGHT: histogram of ‘red’-normalized ‘green’ fluorescence distribution of ‘orange'^OFF^ cells. Inset: 2D density hexbin plot.

### Spreading from ectopic ncRNA nucleators is stochastic and produces intermediate states

We first examined the intrinsic behavior of the heterochromatin spreading reaction in an ectopic context. We constructed the initial ectopic HSS based on a strain where a part of the centromeric ncRNA-driven nucleation element (*dh*) is inserted proximal to the endogenous *ura4* gene (Canzio et al., 2011; Marina et al., 2013). We replaced the *ura4+* open-reading frame (ORF) with ‘green’ to track nucleation element-proximal events. Then, to track distal events, we inserted ‘orange’ at one of several sites downstream from ‘green’ (*ura4::dh*HSS^1kb^*, ura4::dh*HSS^3kb^*, ura4::dh*HSS^5kb^ *ura4::dh*HSS^7kb^, Figure 1B). The noise filter (‘red’) was inserted between *SPBC1711.11* and *SPBC1711.12,* a *bona fide* euchromatic region (Garcia et al., 2015). All strains were initially constructed in a *Δclr4* background, and we initiated heterochromatin formation by crossing in *clr4+*. We assessed heterochromatin formation after ~80–100 generations by quantifying the production of ‘green’ and ‘orange’. This period is significantly longer than ~25 generation timeframe required for full formation of a heterochromatic domain (Obersriebnig et al., 2016), ensuring that the population is at equilibrium.

To quantitatively assess the products of heterochromatin formation, we performed steady-state flow cytometry on log-phase cells, which were size-gated for small, recently divided cells (~91% G2, Figure 1—figure supplement 1D and supplemental experimental materials) to remove size- and cell cycle-related effects. At this stage, we only normalize the cells by the ‘red’ noise filter and scale the signal in each channel to *Δclr4*, giving us a broad overview of the possible expression states of ‘green’ and ‘orange’. We observe no cells that express ‘green’ but repress ‘orange’ (insets, Figure 1C), instead, all cells that are fully or partially ‘orange’ repressed are also robustly ‘green’ repressed. This observation, together with our finding that ‘green’ repression kinetically anticipates ‘orange’ repression (Figure 3—figure supplement 1), is consistent with heterochromatin spreading outward from the *ura4::dh* nucleator. Considering ‘green’ repression thus a proxy for nucleation, we observed that cells populate a wide range of nucleation states rather than a single state, with the distribution of repressed states varying among the HSS distance sensor strains (*ura4::dh*HSS^1-7kb^, Figure 1C). To specifically examine cells that have fully nucleated, we applied a computational ‘nucleation clamp’ that isolates cells with a ‘green’ signal that is lower than the median plus two standard deviations of wild-type cells containing no XFPs (see Appendix 1-Supplemental Materials and methods). Using ‘orange’ as a spreading proxy, we found spreading to be stochastic in nucleated cells, with some cells exhibiting full repression, but others partial repression or full de-repression (*Δclr4*, x = 1) of the ‘orange’ spreading sensor. The proportion of cells that are fully repressed by spreading declines linearly with distance (scheme, Figure 1B; data, Figure 1C). Intriguingly, cells that are not fully repressed mostly exhibit intermediate levels of repression, which are neither at values of full repression or de-repression.

We next assessed the nature of these intermediate states in the 3 kb distance reporter strain, where ~30% of cells had maximal repression at the ‘orange’ locus and the remainder had intermediate states ranging from strongly to weakly repressed. Using Fluorescence Activated Cell Sorting (FACS), we gated for successful nucleation in the ‘green’ channel and then binned the ‘orange’ channel for fully repressed (low), intermediate and de-repressed (high) populations (Figure 1D, cartoon). We queried each bin for molecular events associated with heterochromatin formation, using RT-qPCR to determine the expression levels of ‘orange’, and Chromatin Immunoprecipitation (ChIP) to query the presence of the marks H3K9me2 and H3K4me3. These marks are thought to be mutually exclusive, associating with repressed heterochromatin and active promoters, respectively (Noma et al., 2001). The message level of ‘orange’ is tightly repressed in the ‘low’ population (0.05 of max), partially repressed in the intermediate population (0.3 of max), and nearly fully ‘de-repressed’ (0.8 of max) in the ‘high’ population. Thus, cells with intermediate fluorescence also exhibit partial gene repression, demonstrating that fluorescence accurately reports on gene expression (Figure 1D, RT primers indicated in diagram in 1C, solid line). Histone modification levels also correlated well with the HSS signals (Figure 1E, ChIP primers indicated in diagram in 1C, dashed line). The ‘low’ fluorescence population has high H3K9me2 (0.9 of *dh*, positive control) and low H3K4me3 (0.09 of actin, positive control); the intermediate population had intermediate H3K9me2 (0.49 of *dh*) and H3K4me3 (0.23 of actin), and the high population had low H3K9me2 (0.2 of *dh*) and higher H3K4me3 (0.44 of actin). Hence, successfully nucleated cells with intermediate fluorescence also exhibit intermediate amounts of the mRNA for ‘orange’ and histone marks reflecting heterochromatin (H3K9me2) and transcriptional activity (H3K4me3). These results support the notion that intermediate states of repression observed by cytometry represent intermediate states of spreading.

These observations are not due to the particularities of the ectopic site chosen or the behavior of the XFPs, as our results are recapitulated at the *his1* locus (*his1::dh*HSS^3kb^, Figure 1—figure supplement 1E), which contains only one gene (*rec10*) in the ‘spreading zone’, rather than several transcriptional units. Additionally, switching the nucleation and spreading reporter fluorophores produced similar results (Figure 1—figure supplement 1E). These results suggest that ncRNA-driven heterochromatin spreading at ectopic sites is intrinsically stochastic and multimodal, producing intermediate states of repression.

### Distinct forms of heterochromatin spreading at MAT

**Figure 2.**
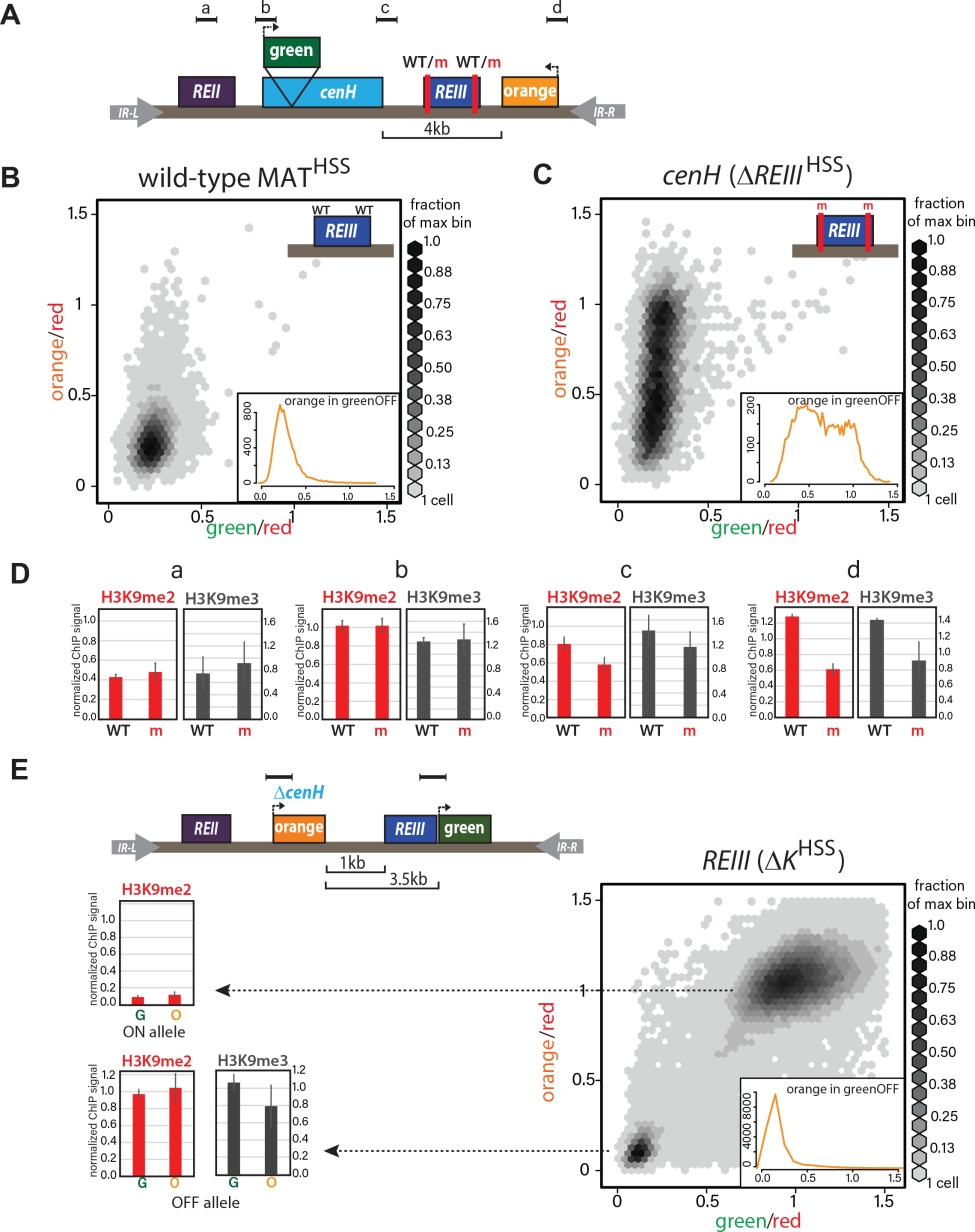
ncRNA-dependent and independent nucleation yields qualitatively different spreading reactions in the MAT locus. (**A**) Diagram of the reporters within MAT^HSS^ and *ΔREIII*^HSS^. WT and m for *REIII* indicate the presence or deletion of the Atf1/Pcr1 binding sites, respectively. (**B**) 2D-density hexbin plot showing the ‘red’-normalized ‘green’ and ‘orange’ fluorescence for wild-type MAT^HSS^ cells. Scale bar shows every other bin cutoff as a fraction of the bin with the most cells. Inset: histogram of the ‘red’-normalized ‘orange’ fluorescence distribution of ‘green'^OFF^ cells. (**C**) 2D-density hexbin plot and inset as above for *ΔREIII*^HSS^, which contains two 7 bp Atf1/Pcr1-binding site deletions (m) within the *REIII* element. (**D**) ChIP for H3K9me2 (red) and H3K9me3 (grey) for amplicons indicated in (**A**). normalized to *dh.* WT, wild-type MAT^HSS^, m, *ΔREIII*^HSS^. (**E**) TOP: diagram of the reporters within *ΔK*^HSS^. The *cenH* nucleator and additional 5’ sequence is deleted and replaced by ‘orange’. ‘green’ is located directly proximal to *REIII* and serves as the nucleation clamp. ChIP amplicons are indicated as black bars. BOTTOM: 2D- density hexbin plot and inset as above. LEFT: ChIP for H3K9me2 (red) and H3K9me3 (grey) for ‘green’ and ‘orange’ in isolated *ΔK*^HSS-ON^ or *ΔK*^HSS-OFF^ alleles. In hexbin plots, the *Δclr4* derivative of each strain was used to normalize the X- and Y-axes to = 1. Error bars indicate standard deviation of technical replicates.

We next examined spreading behavior at the endogenous mating type locus (MAT), which is tightly repressed (Grewal and Klar, 1997; Thon et al., 2002) and a *bona fide* high-fidelity locus, as it can behave in a bistable manner with stable epigenetic inheritance even when disrupted (Grewal and Klar, 1996). The MAT locus has two known elements shown to recruit the H3K9 methylase Clr4: the *cenH* element, homologous to the ncRNA-nucleated *dh* fragment we inserted at *ura4* and *his1*, and the RNA-independent element termed *REIII* (Jia et al., 2004; Thon et al., 1999). At *REIII,* two stress-responsive transcription factors, Atf1 and Pcr1, which form a heterodimer (Wahls and Smith, 1994), recognize two DNA-binding sites within *REIII,* directly recruit Clr4, Swi6/HP1 and histone deacetylases (HDACs) (Jia et al., 2004; Kim et al., 2004) and are required for heterochromatin formation at MAT when *cenH* is compromised (Noma et al., 2004). We validated that MAT retains its well-documented tight repression following insertion of the HSS, placing the ‘green’ reporter within the *cenH* nucleator, and the ‘orange’ reporter proximal to the *REIII* nucleator. Both colors were fully repressed in the large majority of cells (Figure 2B), which is reproduced when the color orientations are reversed (Figure 2—figure supplement 1A). However, for both reporter configurations, the *REIII* proximal color showed a small proportion of cells that are slightly de-repressed compared to the *cenH* internal color, consistent with previous findings (Thon and Friis, 1997). We conclude that the HSS can be used to dissect spreading at the MAT locus.

**Figure 2—figure supplement 1.**
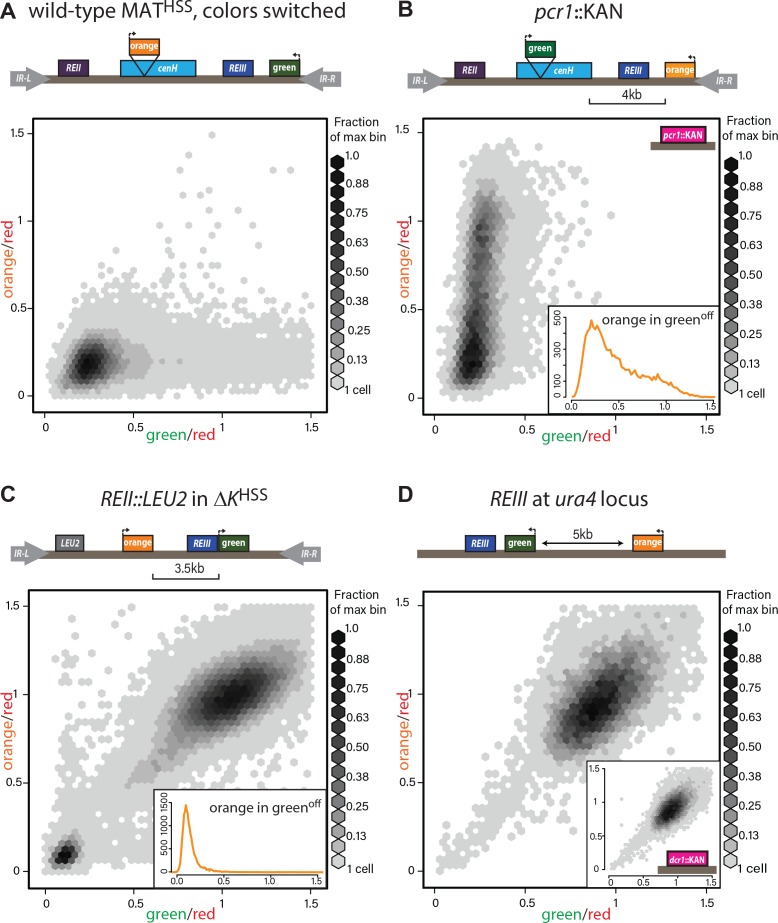
Heterochromatin spreading characteristics of *cis-*acting elements at the tightly repressed MAT locus. (**A**) The MAT^HSS^ documents tight repression of the wild-type MAT locus. As in Figure 2A and B, with ‘green’ and ‘orange’ switched. (**B**) Stochastic spreading with intermediate states in *pcr1::KAN. pcr1* transcription factor was knocked-out in the PAS217 wild-type MAT^HSS^. Plot and inset as in Figure 2B. (**C**) *REII* does not contribute to bimodal distribution seen for *ΔK*^HSS^. The *REII* locus (1 kb) was replaced with the *LEU2* gene before *clr4+* was introduced by cross. (**D**) *REIII* is unable to establish spreading at an ectopic site. 2D density hexbin plots of *ura4::REIII*HSS^5kb^. Normalized green and orange are near 1.0, indicating a failure to repress both reporters. Inset: 2D density hexbin plots of *ura4::REIII*HSS^5kb^ *dcr1::KAN. dcr1* was deleted to release extra heterochromatin factors from RNAi- repressed loci. No additional silencing is detected.

**Figure 2—figure supplement 2.**
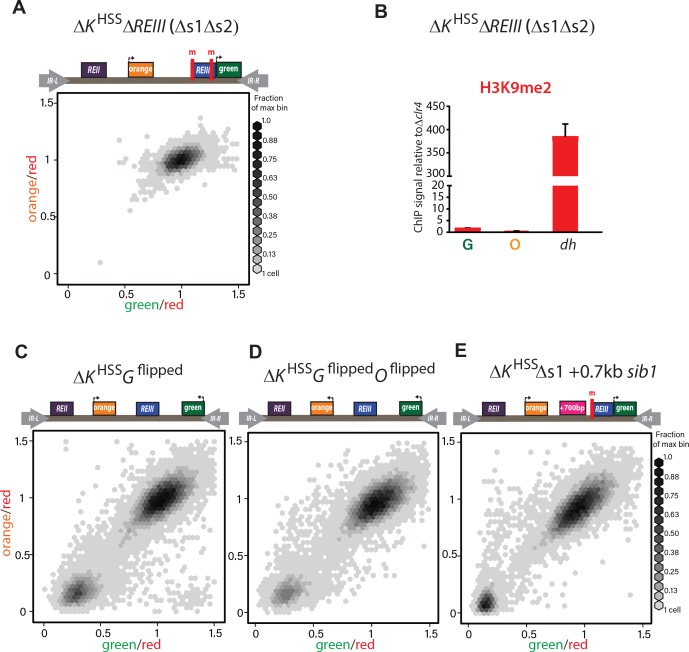
*REIII* is required for heterochromatin formation in *ΔK*^HSS^. (**A**) Deletion of both Atf1-/Pcr1-binding sites before introduction of *clr4+* in *ΔK*^HSS^ blocks gene silencing. In 34/34 strains tested (one representative shown), *ΔK*^HSS^*Δs1Δs2* cannot form repressed states. (**B**) H3K9me2 does not accumulate when both Atf1/Pcr1-binding sites are deleted in *ΔK*^HSS^. H3K9me2 ChIP in *ΔK*^HSS^*Δs1Δs2* at ‘green’, ‘orange’ and *dh.* (*ΔK*^HSS-OFF^ accumulates H3K9me2 to similar extent as *dh,* Figure 2E). Error bars indicate standard deviation of technical replicates. (**C**) ‘green’ orientation and position does not substantially affect *ΔK*^HSS^ behavior. In *ΔK*^HSS^ G^flipped^‘green’ is flipped in orientation with respect to *ΔK*^HSS^. (**D**) ‘green’ and ‘orange’ orientations do not substantially affect *ΔK*^HSS^ behavior. In *ΔK*^HSS^ G^flipped^ O^flipped^‘green’ is located as in C and ‘orange’ is flipped in orientation with respect to *ΔK*^HSS^. ‘green’ in (**C**) and (**D**) is 2.1 kb downstream from its location in *ΔK*^HSS^ now on the distal side of the *mat3m* cassette. (**E**) Increasing distance between *REIII* and ‘orange’ does not substantially affect *ΔK*^HSS^ behavior. The Atf1/Pcr1-binding site proximal to ‘orange’ was deleted (Δs1) and 700 bp of the *sib1* ORF inserted to the left of the Δs1 site. 2D-hexbin plots as in Figure 2.

We then examined spreading in cells nucleated solely by the *cenH* element. The *REIII* nucleator was inactivated by deleting the critical *cis*-acting Atf1/Pcr1-binding sites, to create a strain designated *ΔREIII*^HSS^ (Figure 2C). To our surprise, the high fidelity that the MAT locus exhibits in the repressed state (Grewal and Klar, 1996) disappeared. Instead, *cenH* nucleated spreading in the *ΔREIII* strain behaved similarly to spreading from the ectopic ncRNA-nucleated strains, showing high stochasticity and predominantly intermediate repression states (Figure 2C). We wanted to address if this stochastic silencing is reflected in weakened heterochromatin assembly. We preformed ChIP for H3K9me2 and H3K9me3, marks signaling heterochromatin assembly (Nakayama et al., 2001) and repression or spreading (Al-Sady et al., 2013; Jih et al., 2017; Zhang et al., 2008), respectively. We found that these marks decline progressively towards the distal ‘orange’ reporter in *ΔREIII*^HSS^ (Figure 2D), compared to the wild-type (WT) MAT^HSS^. This is consistent with the observed tight repression for WT MAT^HSS^ (Figure 2B) and weakened silencing at the distal ‘orange’ in *ΔREIII*^HSS^ (Figure 2D). It is possible that this difference in spreading results from an altered heterochromatin structure at *cenH* in *ΔREIII*^HSS^. However, H3K9me2 and me3 accumulation does not differ between *ΔREIII*^HSS^ and WT MAT^HSS^ at the *cenH* nucleator, or the leftward *REII* locus (Figure 2D). Thus, the observed behavior of *ΔREIII*^HSS^ is consistent with stochastic and multimodal spreading, rather than compromised nucleation at *cenH*.

To examine heterochromatin formation independent of *cenH*, we used the historical *ΔK* strain, where the entire *cenH* nucleation element is deleted and replaced with a *ura4+* reporter (Grewal and Klar, 1996). We introduced the HSS into this context (*ΔK*^HSS^, Figure 2E), placing the ‘green’ reporter proximal to *REIII* and the ‘orange’ reporter distally, replacing *ura4*. We then introduced *clr4+* by cross and directly cultured colonies derived from germinated *clr4+* spores. We found that although *ΔK*^HSS^ has very weak nucleation compared to strains with intact ncRNA nucleators, the distribution of cells is sharply bimodal: Cells were either repressed at both reporters (‘OFF’, lower left corner) or de-repressed at both reporters (‘ON’, upper right corner; Figure 2E). We note that isolation of single colonies on nonselective media from original spores of the cross yields mostly ON (*ΔK*^HSS-ON^) or OFF (*ΔK*^HSS-OFF^) colonies, consistent with each state being metastable (Grewal and Klar, 1996; Thon and Friis, 1997). This heterochromatin formation pattern requires *REIII,* as in 34/34 strains tested, no silencing can be established if Atf1/Pcr1 binding sites are deleted before *clr4+* is introduced (Figure 2—figure supplement 2A,B). Additionally, the bimodal behavior does not require the H3K9me-independent gene-repressive *REII* element (Hansen et al., 2011), as *ΔK*^HSS^ *REII::LEU2*, containing a deletion of *REII*, behaved similarly to *ΔK*^HSS^ (Figure 2—figure supplement 1C), and is further independent of reporter placement (Figure 2—figure supplement 2C,D). We next characterized the molecular signature of the locus. While in our two color plots cells that were repressed in ‘green’ did not show any de-repression in ‘orange’ (Figure 2E, cells in bottom left corner), we wanted to test if the heterochromatic state at these loci correlated with this silencing pattern. Since we can isolate *ΔK*^HSS-ON^ and *ΔK*^HSS-OFF^ alleles by simple plating of *ΔK*^HSS^ cells, we performed H3K9me2 ChIP on both and H3K9me3 ChIP for *ΔK*^HSS-OFF^ cells (not detectable for *ΔK*^HSS-ON^). We found that methylation correlates with the repression state (Figure 2E) and importantly, does not significantly differ between ‘green’ and ‘orange’. Together, these result indicate that in *ΔK*^HSS-OFF^ cells heterochromatin spreading is continuous across the locus and does not, unlike *cenH-*triggered spreading, accumulate any intermediates.

### Multi-generational single-cell imaging reveals ncRNA-driven spreading to be unstable

**Figure 3.**
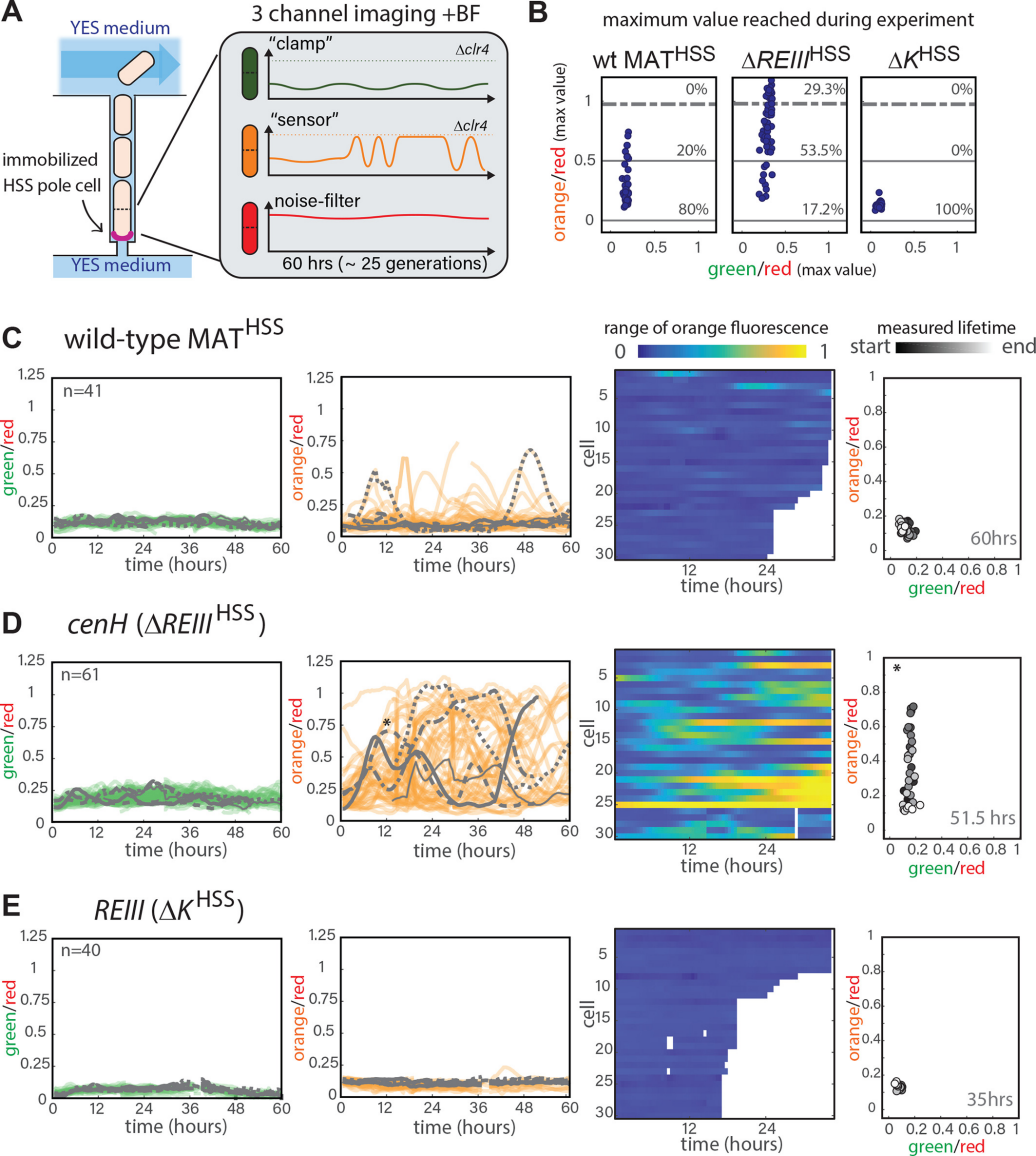
Single-cell analysis of nucleation and spreading using a Fission Yeast Lifespan Micro-dissector (FYLM). (**A**) Overview of the FYLM-based heterochromatin spreading assay. The old-pole cell is trapped at the bottom of one of hundreds of wells in the FYLM microfluidic device and is continuously imaged in brightfield (to enable cell annotation), green, orange and red channels. Hypothetical example traces are shown. (**B**) Maximum values attained by each nucleated cell for normalized ‘orange’ plotted against normalized ‘green’. Solid horizontal lines correspond to y = 0 and y = 0.5. Dashed line corresponds to an ON cutoff determined by mean less three standard deviations for each strain’s matched *Δclr4* strain. Percentage of cells between each line was calculated. (**C**) FYLM analysis of wild-type MAT^HSS^ cells. CELL TRACES: 60 hr of normalized ‘green’ (left) and ‘orange’ (right) fluorescence in cells that maintained nucleation with the same five cells overlaid in different gray line styles in both plots. Gaps indicate loss of focus. HEATMAP: Up to 36 hr of normalized ‘orange’ fluorescence for 30 cells that maintained nucleation is represented from blue (0) to yellow (1). X-Y FLUORESCENCE PLOT: for one representative sample cell, plot of normalized ‘green’ and ‘orange’ fluorescence across its measured lifetime (grayscale). (**D**) FYLM analysis of *ΔREIII*^HSS^ cells as in C. The example cell in the X-Y dot plot is marked with an asterisk(*) on the orange traces (**E**) FYLM analysis of *ΔK*^HSS-OFF^ isolate, as in C., D. All cells were normalized to *Δclr4* (max, 1).

Our measurements thus far cannot reveal the dynamics of transitions between states. This requires long-term imaging of cells over a substantial number of generations (>20), which is difficult with traditional microscopy because of cell crowding effects. To deal with this issue, we used the Fission Yeast Lifespan Micro-dissector (FYLM) microfluidic device (Spivey et al., 2017, 2014), which traps the old pole of a rod shaped *S. pombe* cell at the bottom of a chamber well for its entire lifetime. Sibling cells generated at the new pole by medial fission eventually exit the chamber. We continuously image the old-pole cell with fluorescence microscopy for up to 60 hr (Figure 3A). We note that unlike *Saccharomyces cerevisiae*, *S. pombe* does not execute an aging program but rather dies stochastically (Coelho et al., 2013; Nakaoka and Wakamoto, 2017; Spivey et al., 2017). Thus, imaging *S. pombe* over long timescales avoids the confounding effects of aging on epigenetic behavior (Guarente, 2000; Li et al., 2017). To capture the long-range dynamics of spreading, we imaged approximately one hundred cells of each strain concurrently (see Figure 3—figure supplement 2B for a summary of cell fates in all experiments). For each cell, we imaged all three channels continuously, and performed similar normalizations as for the flow cytometry data (Appendix 1-Supplemental Materials and methods). We first imaged the HSS distance sensor strain (ectopic *ura4::dh*HSS^3kb^). Our ability to observe cells that were initially fully de-repressed allowed us to trace ‘green’ and ‘orange’ repression kinetically. Consistent with linear heterochromatin spread outward of the *dh* nucleator, we find that ‘orange’ repression is anticipated by repression at ‘green’ (Figure 3—figure supplement 1). While nucleation in this strain is not stable (likely due to ‘green’ being adjacent to, rather than within *dh*), over time intervals where nucleation does persist, we observed dynamic fluctuations in the distal ‘orange’ color without a fixed temporal pattern (Figure 3—figure supplement 2A and Figure 3—videos 1 and 2), which is not due to the repression state of ‘green’ (Figure 3—figure supplement 2F).

**Figure 3—figure supplement 1.**
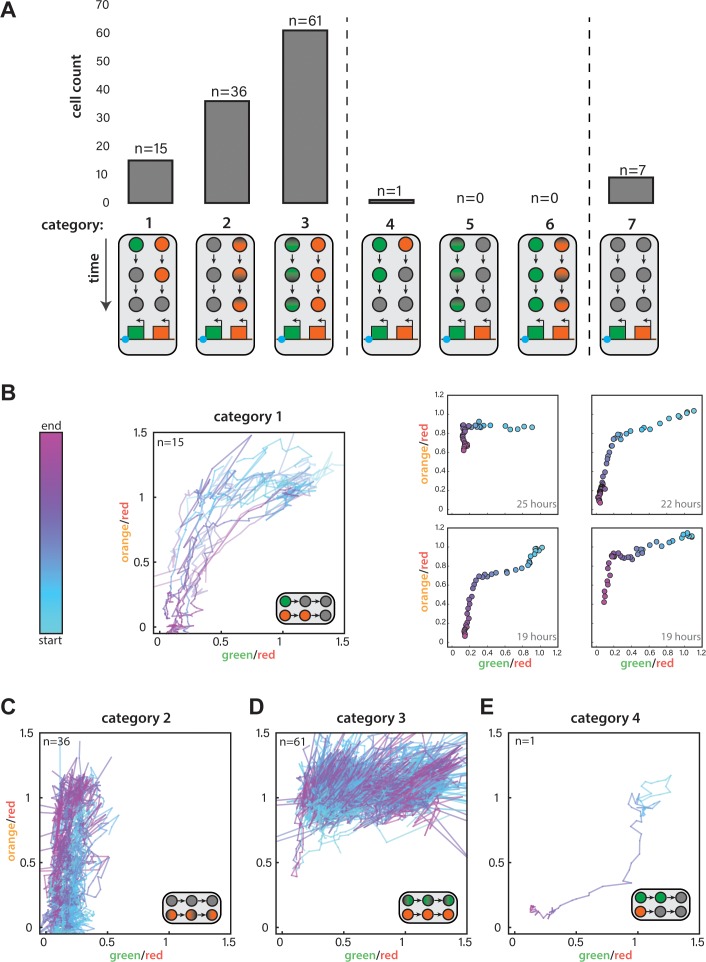
Single-cell analysis of nucleation and spreading using a Fission Yeast Lifespan Micro-dissector (FYLM). (**A**) For *ura4::dh*HSS^3kb^ FYLM experiments, counts of cells in each of seven categories. Diagrams indicate the time-dependent silencing behaviors of cells in each category. Categories 1–3 are consistent with proximal to distal silencing, whereas categories 4–6 are consistent with a distal to proximal silencing. (**B**) Time-dependent traces showing cells from Category 1 where the normalized ‘green’ and ‘orange’ values at each time point are plotted color-coded by time where blue and pink represent the start and end of the measurement, respectively. LEFT: Traces for all Category 1 cells, which begin at the start of the silencing event with both colors fully expressed and end when both colors have reached their local minimum. RIGHT: Four example cells where points represent 30-min time points colored from the start to end of the event. The duration of the time represented is indicated in the lower right corner. (**C**) Traces for Category 2 cells during their entire measured lifespan. (**D**) Traces for Category 3 cells during their entire measured lifespan. (**E**) Time-dependent traces for the one cell in Category 4. Lines are plotted and time is curated as in (**B**).

**Figure 3—figure supplement 2.**
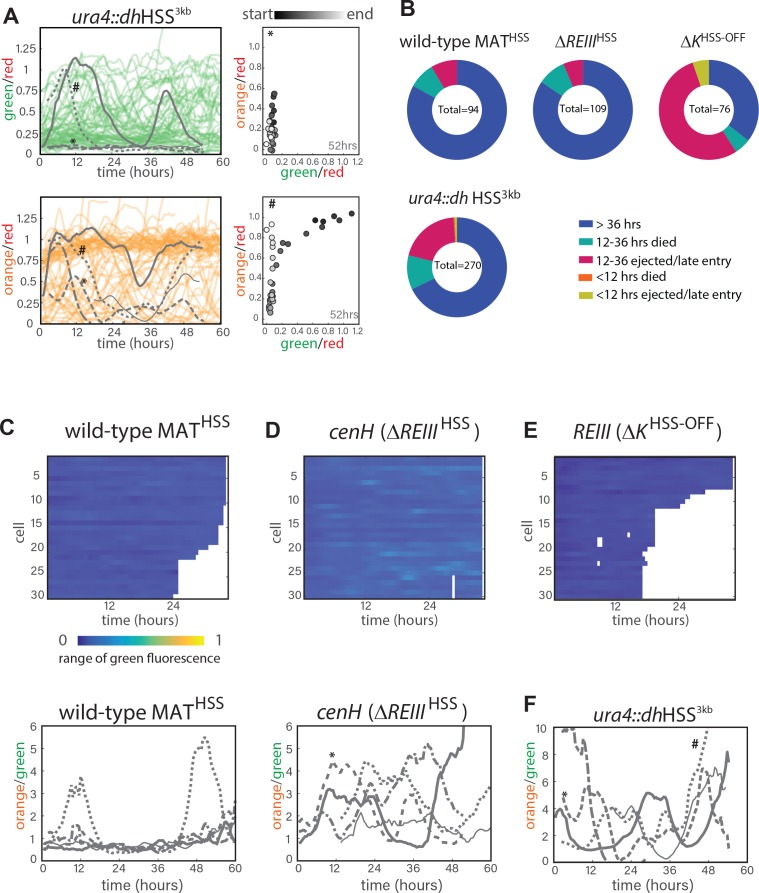
Single-cell analysis of nucleation and spreading using a Fission Yeast Lifespan Micro-dissector (FYLM). (**A**.) FYLM analysis of *ura4::dh*HSS^3kb^ cells. TOP LEFT: 60 hr of normalized ‘green’ fluorescence, a subset of cells are shown for clarity. five example cells are overlaid in gray each with different line types. BOTTOM LEFT: 60 hr of normalized ‘orange’ fluorescence in the matching subset of cells with the same five overlaid in gray. *, # represent two example cells. RIGHT: for two representative sample cells imaged, plots of normalized ‘green’ and ‘orange’ across its measured lifetime (grayscale). The corresponding cells are marked in the orange traces on LEFT. (**B**) Categorization of cell longevity of all cells analyzed in the FLYM experiment. Measured lifespan ends when a cell dies or is ejected from its capture channel. (**C**) For wild-type MAT^HSS^ TOP: ‘green’ fluorescence heatmap (blue (0) to yellow (1)) for the same 30 cells as in 3C. BOTTOM: 60 hr of traces for ‘orange’ divided by ‘green’ for the five example cells indicated in 3C. (**D**) ‘green’ fluorescence heatmap and ‘orange’/”green’ traces for *ΔREIII*^HSS^ as in C. (**E**) ‘green’ fluorescence heatmap *ΔK*^HSS^ as in C. (**F**) ‘orange’/”green’ traces for *ura4::dh*HSS^3kb^ as in C. *, # indicate the same cells as in A.

**Figure 3—video 1.** Cell #274 from strain PAS244. This movie consists of imaging in four channels, listed from top to bottom: Bright field, ‘green’, ‘orange’, and ‘red’ for cell #274 from the strain PAS244 ura4HSS^3kb^. X-Y fluorescence plot for this cell is shown in Figure 3—figure supplement 2A, top right.

**Figure 3—video 2.** Cell #271 from strain PAS244. This movie consists of imaging in four channels, listed from top to bottom: Bright field, ‘green’, ‘orange’, and ‘red’ for cell #271 from the strain PAS244 ura4HSS^3kb^. X-Y fluorescence plot for this cell is shown in Figure 3—figure supplement 2A, bottom right.

**Figure 3—video 3.** Cell #350 from strain PAS389. This movie consists of imaging in four channels, listed from top to bottom: Bright field, ‘green’, ‘orange’, and ‘red’ for cell #350 from the strain PAS389 WT MAT^HSS^. X-Y fluorescence plot for this cell is shown in Figure 3C. Fluctuations of colors in this video occur over a narrow range (see Figure 3C RIGHT) and are amplified due to relative scaling in the video with respect to background.

**Figure 3—video 4.** Cell #407 from strain PAS391. This movie consists of imaging in four channels, listed from top to bottom: Bright field, ‘green’, ‘orange’, and ‘red’ for cell #407 from the strain PAS391 *ΔREIII*^HSS^. X-Y fluorescence plot for this cell is shown in Figure 3D.

**Figure 3—video 5.** Cell #123 from strain PAS387. This movie consists of imaging in four channels, listed from top to bottom: Bright field, ‘green’, ‘orange’, and ‘red’ for cell #123 from the strain PAS387 *ΔK*^HSS^. X-Y fluorescence plot for this cell is shown in Figure 3E.

Next, we analyzed the MAT locus strains and selected cells that maintained nucleation for their entire measured lifespan (Appendix 1-Supplemental Materials and methods). Under this constraint, the three strains exhibit vastly different behaviors (Figure 3B). WT MAT^HSS^ cells maintained ‘orange’ repression for the majority of their measured lifespans (Figure 3C, Figure 3—figure supplement 2C and Figure 3—video 3). However, we documented transient loss of ‘orange’ silencing for 20% of the cells. (Figure 3B and C). In contrast, while most cells stay similarly nucleated in *ΔREIII*^HSS^ (Figure 3D, Figure 3—figure supplement 2D) 83% of the cells imaged experienced at least half-maximal ‘orange’ de-repression at some time points (Figure 3B). For this strain, 30% of the cells transited through the fully ON state (Figure 3B and D, Figure 3—figure supplement 2D and Figure 3—video 4). In fact, cells sampled a wide range of values from OFF to fully ON, indicating that cells do not occupy ON or OFF states exclusively, but adopt intermediate values across time (Figure 3D). Importantly, *ΔREIII*^HSS^ cells, just as *ura4::dh*HSS^3kb^ cells, fluctuate in their ‘orange’ values, indicating that spreading is unstable and adopts a random walk type behavior. To analyze *ΔK*^HSS^ cells, which exist predominantly in fully ‘green’ and ‘orange’ ON state (Figure 2C), we analyzed *ΔK*^HSS-OFF^ cells (see above). *ΔK*^HSS-OFF^ behaved markedly differently from *ΔREIII*^HSS^: in all the cells analyzed, ‘green’ and ‘orange’ reporters remained OFF throughout the time course (Figure 3B,E and Figure 3—video 5), up to 25 generations, revealing a fundamentally different dynamic behavior between *cenH-* and *REIII-*dependent heterochromatin. We note it remains possible that isolation of a *ΔK*^HSS-OFF^ colony may bias our analysis against potentially more frequent OFF-ON switching events in the primary mixed population derived from continuous propagation of the germinated spore (Figure 2E). However, since the mixed population resolves spontaneously into ON and OFF states once plated, and OFF cells behave similarly in either the mixed *ΔK*^HSS^ or *ΔK*^HSS-OFF^ isolated populations (compare Figure 2E and Figure 5C), we believe the stability of *ΔK*^HSS-OFF^ is intrinsic to the *ΔK* MAT locus.

### Epigenetic stability at MAT is dependent on *REIII*

**Figure 4.**
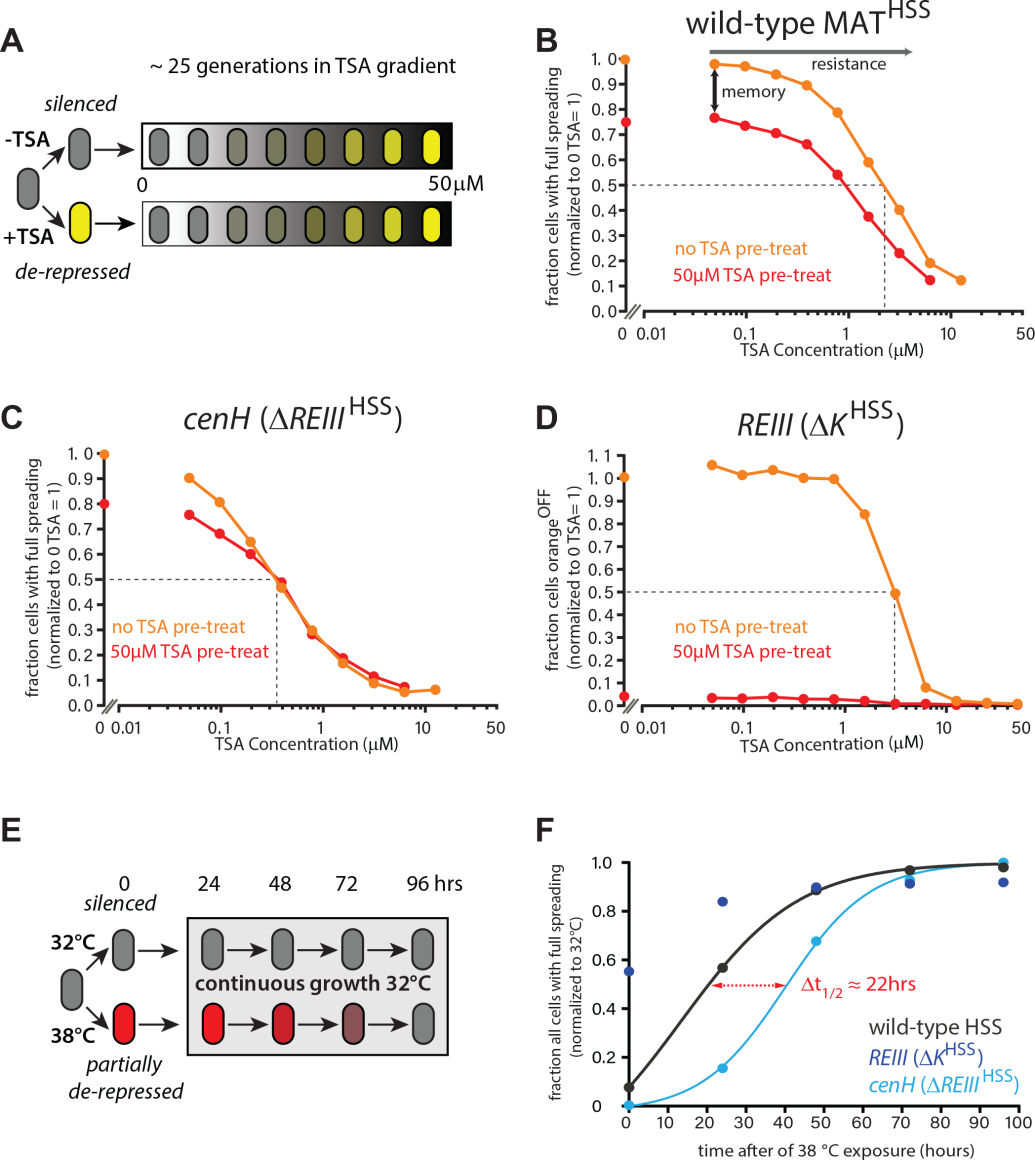
ncRNA-nucleated spreading exhibits weak memory and resistance in the absence of *REIII*. (**A**) Experimental schematic for memory and resistance measurements. Cells in log phase were treated with TSA (50 μM) for 10 generations to erase all heterochromatin (de-repressed, yellow) or kept untreated (repressed, gray). Both populations are then grown in a gradient of TSA concentration from 0 to 50 μM for 25 generations. (**B**) The wild-type MAT locus exhibits memory in silencing ‘orange’ throughout the TSA gradient. The fraction of ‘green'^OFF^ cells that fully silence ‘orange’ normalized to the no TSA pre-treatment, 0 μM TSA point are plotted for each TSA concentration. Red line: cell ancestrally TSA pre-treated; light orange line: cells without pre-treatment. (**C**) Spreading from *cenH* exhibits weak memory and low resistance. Cell populations as above. (**D**) ncRNA-independent spreading exhibits high resistance. The fraction of ‘orange'^OFF^ for all cells is plotted, because in the TSA pre-treatment almost no ‘green'^OFF^ cells can be detected. Dotted lines indicate the half-resistance points: TSA concentration at which 50% of non-pretreated cells fail to form heterochromatin at ‘orange’. Memory is the difference between orange and red lines. One of two full biological repeats of the experiment is shown.( **E**) Experimental schematic for heat stress and recovery. Cells were grown at either 32 or 38°C for 10 generations and strains subsequently grown continuously for 96 hr at 32°C. (**F**) The fraction of cells with full spreading (‘green'^OFF^ and ‘orange'^OFF^) after 38°C exposure and recovery normalized to the fraction of cells with full spreading at 32°C for each strain is plotted over time. For wild-type MAT^HSS^ and *ΔREIII*^HSS^ strains, we fit a simple sigmoidal dose response curve and determined a t_1/2_ value. The difference in t_1/2_ values or Δt_1/2_ is ~22 hr or ~9–10 generations.

To probe memory capacity (i.e. the ability of cells to retain information of an ancestral state established many generations prior), we compared cells containing an intact MAT locus to those lacking either ncRNA- or *REIII*-dependent heterochromatin. We established two ancestral states (Figure 4A); one with unperturbed heterochromatin, and a second treated with the HDAC inhibitor trichostatin A (TSA), known to fully disrupt the heterochromatin state ([Hall et al., 2002] and Figure 4—figure supplement 1). Following production of the ancestral states, we grew cells either in rich media alone or in a TSA concentration gradient (0–50 µM) for 25 generations and then measured the fraction of fully nucleated cells that effectively silence the ‘orange’ spreading marker (Figure 4A). Cells exhibit memory if the fraction of the population with full spreading (‘orange'^OFF^) depends on the ancestral state, which would be indicated by separation of the unperturbed (light orange) and perturbed (red) lines. In contrast, no memory is indicated by convergence of the two lines (graphs in Figure 4B–D). We further measure a second parameter we term relative ‘resistance’, which is defined as the TSA concentration at which the fraction of cells with ‘orange'^OFF^ declines to 50% of the no TSA pretreatment value. This value reports on the intrinsic sensitivity to perturbation of the locus formed by spreading.

**Figure 4—figure supplement 1.**
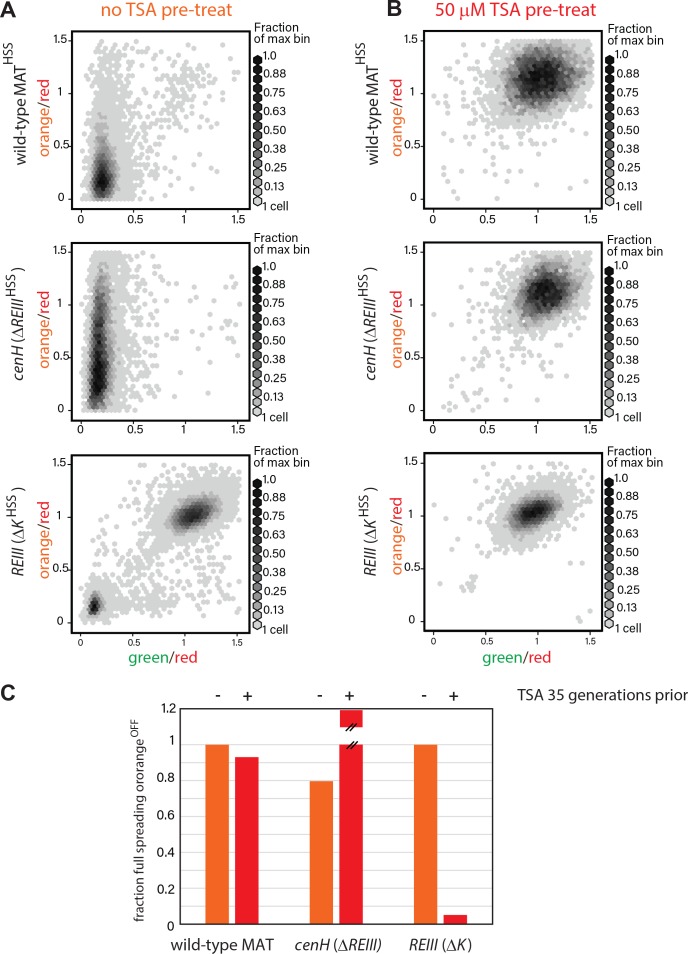
heterochromatin behaviors during TSA treatment and after 35 generations. (**A**) 2D density hexbin plots of wild-type MAT^HSS^, *ΔREIII*^HSS^, and *ΔK*^HSS^ strains grown 10 generations without TSA. (**B**) 2D density hexbin plots of wild-type MAT locus^HSS^, *ΔREIII*^HSS^, and *ΔK*^HSS^ strains grown 10 generations in 50 μM TSA. The density distributions are near 1.0 in all strains indicating complete erasure of heterochromatin. (**C**) History dependence at 35 generations after pretreatments. The fraction of cells with full spreading (wild-type MAT and *ΔREIII*) or fraction of cells with orange^OFF^ (*ΔK*) normalized to the highest value for ancestrally untreated cells (=1) is shown for the 0 µM TSA point. TSA pretreated cells for *ΔREIII*^HSS^ show higher repression than untreated cells. We interpret this to indicate experimental variations in silencing in the absence of memory. This is because for all other circumstances, TSA treatment results in reduced spreading, including for *ΔREIII*^HSS^ at 25 generations post-treatment.

**Figure 4—figure supplement 2.**
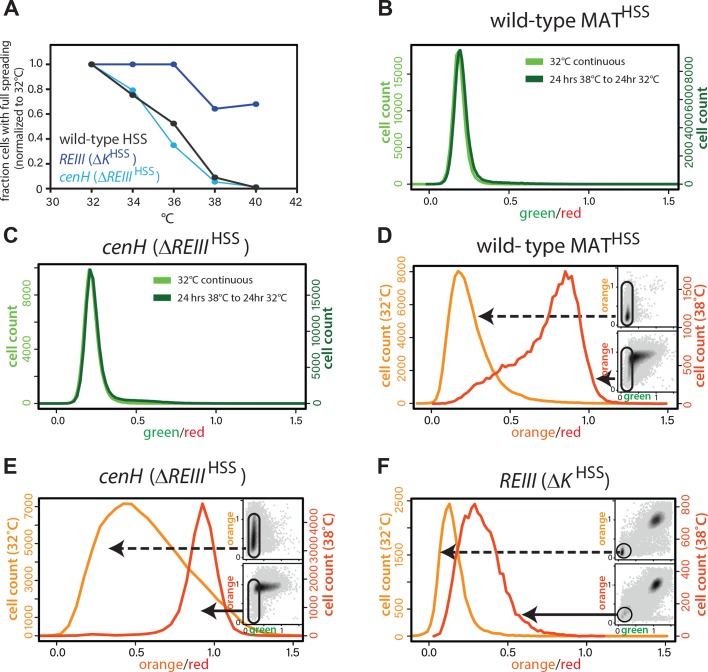
Behavior of MAT heterochromatin at elevated temperature. (**A**) The resistance of the heterochromatin state from 32°C to 40°C in wild-type MAT^HSS^, *ΔK*^HSS^, and *ΔREIII*^HSS^. The fraction of cells that fully repress both ‘orange’ and ‘green’ (full spreading) at each temperature is plotted normalized to the given strains value at 32°C. (**B** and **C**) nucleation is recovered within 24 hr at 32°C. 1-D histogram showing the distribution of green fluorescence in wild-type MAT locus^HSS^ (**B**) or *ΔREIII*^HSS^ (**C**) cells grown either for 48 hr continuously at 32°C (left y-axis, light green) or heat stressed for 24 hr at 38°C followed by 24 hr growth at 32°C (right y-axis, dark green). (**D–F**) Histograms of ‘red’-normalized ‘orange’ fluorescence distribution in ‘green'^OFF^ cells are shown for cells grown at both 32°C (light orange) and 38°C (dark orange). Insets: 2D density hexbin plots, ‘green'^OFF^ cells are schematically circled. (**C-E**) represent t = 0 in Figure 4F.

As expected, wild-type MAT^HSS^ exhibited clear memory at 25 generations (Figure 4B), which was still weakly evident even at 35 generations (Figure 4—figure supplement 1C). Among fully nucleated (‘green'^OFF^) cells, those that derived from untreated ancestral cells showed a greater fraction of silencing (‘orange'^OFF^) than those derived from treated cells throughout the entire TSA gradient, with a half-resistance point of ~2 µM (Figure 4B). Thus, wild-type MAT^HSS^ memory is robust in the face of perturbations of the heterochromatic state.

In sharp contrast, when spreading exclusively nucleates from *cenH* (*ΔREIII*^HSS^ strain), memory of silencing (‘orange'^OFF^) is significantly weaker. Memory collapsed beyond low TSA concentrations (>0.2 µM TSA), with the red and light orange lines coinciding for much of the gradient. Even at 0 μM TSA, history dependence was erased at 35 generations (Figure 4—figure supplement 2C). Interestingly, the half-resistance point was ~0.2 μM, 10-fold lower than that of wild-type MAT (Figure 4C). As *cenH-*nucleated spreading in *ΔREIII*^HSS^ produces little memory capacity and lacks resistance, the memory capacity at MAT does not derive from ncRNA-nucleated spreading. These results are consistent with *REIII* being required for the memory behavior of WT-MAT.

The *ΔK*^HSS^ strain at face value had the widest separation in the behavior of the progeny of TSA pretreated and untreated cells. However, ascribing this behavior directly to memory is complicated by the fact that *ΔK*^HSS^ cells are no longer able to re-nucleate if they were ancestrally TSA treated, consistent with previous findings indicating that RNAi factors are required for heterochromatin establishment at MAT (Hall et al., 2002). However, when examining resistance, that is the behavior of cells not ancestrally TSA pretreated, we observe that the *REIII* dependent *ΔK*^HSS^ strain has a half-resistance point of ~3 µM TSA (Figure 4D), similar to the intact locus. This indicates that the increased resistance of the wild-type over *ΔREIII*^HSS^ is conferred by *REIII.* Together these results indicate that *REIII* is required for epigenetic stability at MAT.

### *REIII* imposes epigenetic behavior under environmental stress conditions

We next studied how *REIII* contributes to epigenetic stability in the context of a physiological perturbation, such as change in ambient temperature. Consistent with previous reports, we found that ncRNA-nucleated spreading is sensitive to continuous growth at high temperature, likely due to the cytosolic shuttling of RNAi-components (Woolcock et al., 2012; Figure 4—figure supplement 2A). WT MAT behaved in a similarly sensitive manner. In contrast, heterochromatin in *ΔK*^HSS^ cells was highly resistant to elevated temperature (Figure 4—figure supplement 2A).

We next probed the ability to remember the heterochromatin state after a transient exposure to elevated temperature, by exposing cells to 38˚C for 10 doublings, followed by return to growth at 32˚C (Figure 4E). As expected from our steady-state experiments above, *REIII*-dependent heterochromatin (*ΔK*^HSS^ cells) is only minimally affected by the perturbation and regains full spreading rapidly (Figure 4F, Figure 4—figure supplement 2F), whereas WT MAT and ncRNA-nucleated (*ΔREIII*^HSS^) strains lose a significant amount of spreading (Figure 4F, Figure 4—figure supplement 2D,E) and nucleation (Figure insets). Both strains regain nucleation at *cenH* rapidly (1 day after return to 32˚C; Figure 4—figure supplement 2B,C). However, they are discrepant in their kinetics of restoration to the 32˚C extent of spreading, with WT MAT recovering much more rapidly than the strain nucleated exclusively by ncRNA (∆*REIII*^HSS^) (Figure 4F). Indeed, plot fitting reveals a half-life (t_1/2,_ time to reach 50% of initial state) difference of ~22 hr, or ~9–10 generations between WT MAT and *ΔREIII*^HSS^ (Figure 4F). Therefore, *REIII*- is required for efficient recovery to the fully repressed state after heat perturbation. These data suggest that a central role of *REIII* is to ensure that epigenetic stability at MAT is maintained in the face of environmental perturbations in the wild.

### Stability of heterochromatin in the absence of *cenH* and *REIII trans-*acting factors

**Figure 5.**
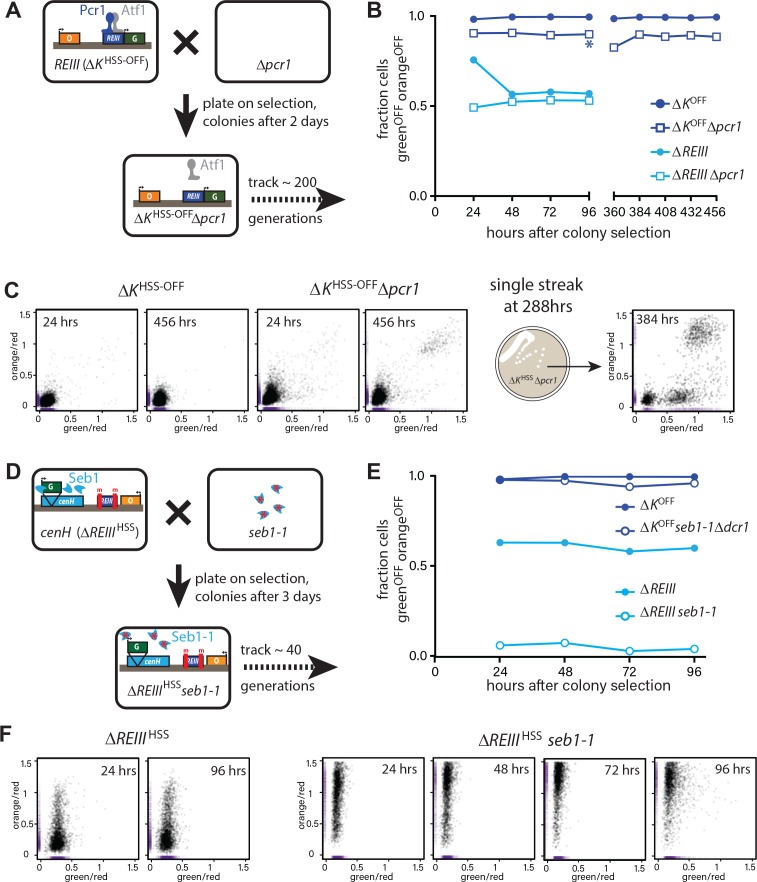
Differential inheritance of ncRNA-dependent and independent spreading in the absence of nucleation factors. (**A**) Scheme for removal of Pcr1 (*REIII* binding factor) in the *ΔK*^HSS^ strain OFF isolate (*ΔK*^HSS-OFF^). Progeny of the cross was selected for *ΔK*^HSS-OFF^*Δpcr1* genotype and identifiable colonies immediately grown for cytometry, and passaged for 456 hr. (**B**) Stable inheritance of repression in *ΔK*^HSS-OFF^*Δpcr1. ΔK*^HSS-OFF^*Δpcr1* or *ΔK*^HSS-OFF^ cells (dark blue lines) where analyzed by flow cytometry over consecutive days, the break indicating passaging without analysis. *Δpcr1* had no significant effect on *ΔREIII*^HSS^ (light blue lines). (**C**) LEFT: scatter plots with partial point transparency of *ΔK*^HSS-OFF^ or *ΔK*^HSS-OFF^*Δpcr1* early and late in the time course. RIGHT: In the middle of the time course (asterisk in (**B**)), *ΔK*^HSS-OFF^*Δpcr1* were struck for single colonies. The scatter plots for one of the isolates is shown. (**D**) Scheme for removal of functional Seb1 in *ΔREIII*^HSS^ strain. Selection and growth as in A., total passaging time 96 hr. (**E**) Weak inheritance of repression in *ΔREIII*^HSS^*seb1-1* (light blue lines). Analysis as above, total time course 96 hr. Removal of both Seb1 and RNAi pathways (*ΔK*^HSS-OFF^*seb1-1Δpcr1*) does not affect maintenance of silencing (dark blue lines). (**F**) Scatter plots of *ΔREIII*^HSS^ at 24 and 96 hr and through the entire time course for *ΔREIII*^HSS^*seb1-1.* In these scatter plots, X and Y values of each cell are represented by purple dashes along the corresponding axis.

To address dependence of the epigenetic maintenance of spreading on nucleation following heterochromatin establishment, we examined the behavior of cells following the removal of *trans-*acting factors required for the initial recruitment of nucleation factors such as Clr4, Swi6/HP1 and HDACs. This experiment is similar to the induced removal of the *cis-*acting sites in *S. cerevisiae* (Cheng and Gartenberg, 2000). *∆REIII*^HSS^ and ∆*K*^HSS-OFF^ isolate cells (see above, derived from nonselective plating of ∆*K*^HSS^) with established heterochromatin were crossed to mutants disrupting recruitment of nucleation factors at each element (Figure 5A). To impair *REIII*, we crossed the ∆*K*^HSS-OFF^ reporter strain to ∆*pcr1* (Noma et al., 2004). To impair ncRNA nucleation, we crossed the *∆REIII*^HSS^ reporter strain to *seb1-1,* a mutant allele of the Seb1 RNA binding protein. Seb1 functions redundantly with the RNAi pathway in ncRNA nucleation, including binding *cenH* transcripts, and the mutant allele *seb1-1* has defects in triggering nucleation at *dh* and *dg* pericentromeric elements (Marina et al., 2013). We focus on Seb1, as RNAi pathway mutants have little discernable effect on MAT when introduced after establishment (our unpublished data and [Hall et al., 2002]), indicating a stronger role for Seb1. Identifiable *∆REIII*^HSS^*seb1-1* and ∆*K*^HSS-OFF^*∆pcr1* colonies were grown for flow cytometry analysis immediately following mating and selection. The control cross mutant strains *∆REIII*^HSS^*∆pcr1* and ∆*K*^HSS-OFF^*seb1-1∆dcr1* (loss of all ncRNA-nucleation [Marina et al., 2013]) allowed us to assess any effects the *trans-*factor may have even in the absence of its cognate site of action.

**Figure 5—figure supplement 1.**
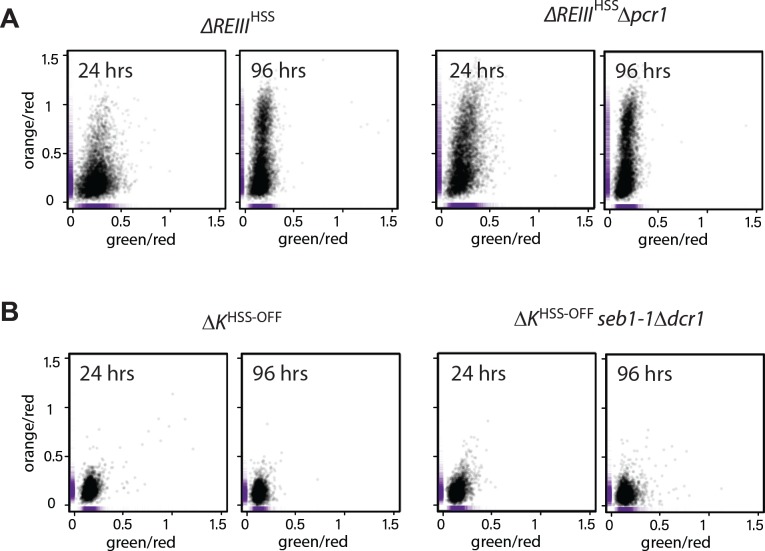
*trans-*factor mutants do not substantially affect spreading when their cognate *cis-*acting element is inactivated. (**A**) Scatter plots of *ΔREIII*^HSS^ and *ΔREIII*^HSS^*Δpcr1* at 24 and 96 hr. (**B**) Scatter plots of *ΔK*^HSS^ and *ΔK*^HSS^*seb1-1Δdcr1* at 24 and 96 hr. The *seb1-1* and *Δdcr1* double mutant should abolish all RNA-dependent nucleation (Marina et al., 2013). The X and Y values of each cell are represented by purple dashes along the axis.

Strikingly, most ∆*K*^HSS-OFF^∆*pcr1* cells remains robustly repressed over 456 hr, around 200 generations (Figure 5B). However, removal of Pcr1 does have a small discernable effect, as the ∆*K*^HSS-OFF^∆ *pcr1* strain showed a small population of cells not completely in the OFF state compared to the ∆*K*^HSS-OFF^ parent (Figure 5C LEFT). Further, by ~400 hr we detected a small fully ON population absent in the parent. This behavior is broadly consistent with the reported stability of intact ∆*K*^OFF^ (switch rate of ~10^−4^ generation, Grewal and Klar, 1997; Thon and Friis, 1997), even though our assay appears to show even smaller ON populations. Very small ON populations are more apparent in a growth selection based assay as only the targeted population survives, as opposed to our assay, which captures all cells. We note a formal possilibty remains that selection of OFF colonies yields higher apparent stability. To get a closer view of the behavior of individual isolates from the population, after 288 hr of continuous passage, we streaked for single colonies and measured the resulting populations. While 5/6 isolates behaved like the broader population, we found 1/6 isolates that experienced more severe breakdown in its heterochromatic state (Figure 5C RIGHT). In this isolate heterochromatin collapsed in a manner not ordered with respect to *REIII* proximity and exhibited ‘green”^ON^/‘orange”^OFF^ cells. In contrast, *∆REIII*^HSS^*seb1-1* lost most spreading at the first measurement point (24 hr, Figure 5E) with progressively increasing de-repression of ‘orange’, but also some loss of ‘green’, over the next 72 hr (Figure 5F). This suggests that the epigenetic inheritance *cenH*-spreading requires continuous nucleation, at least via the Seb1 pathway, consistent with the behavior at synthetic nucleators (Audergon et al., 2015; Ragunathan et al., 2015).

### *REIII-,* but not *cenH-*dependent heterochromatin suppresses histone turnover

**Figure 6.**
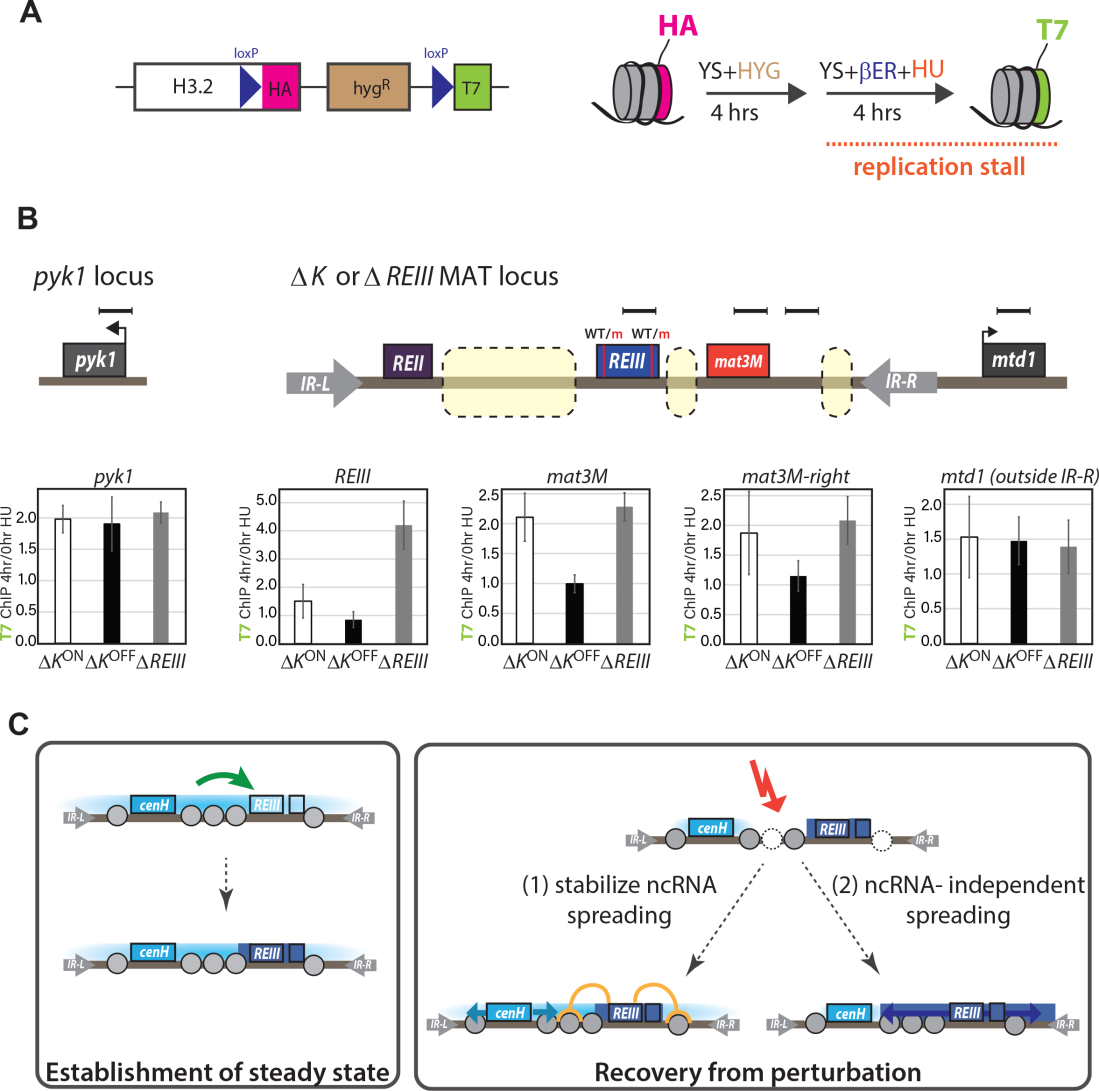
Histone turnover correlates with epigenetic stability in ncRNA-dependent and *REIII-*dependent heterochromatin. (**A**) LEFT: Overview of the RITE system for histone 3.2. Cre recombinase allows tag exchange from HA to T7. RIGHT: experimental scheme for detecting replication-independent H3 turnover. Cells were grown to log phase and then grown for 4 hr in the presence of β-estradiol and 15 mM hydroxyurea. (**B**) Enrichment for H3-T7 at indicated loci in *ΔK*^HSS-ON^*, ΔK*^HSS-OFF^or *ΔREIII*^HSS^ strain. TOP: Location of amplicons for T7-ChIP indicated by bars. Dashed boxes in MAT indicated regions of genomic difference between *ΔK*^HSS^ and *ΔREIII*^HSS^. WT and m for *REIII* indicate presence or deletion of Atf1/Pcr1-binding sites, respectively. BOTTOM: Enrichment of T7 tag by ChIP at 4 hr in HU over 0 hr for indicated strains. one indicates no enrichment over 0 hr. Error bars indicate standard deviation of technical replicates. (**C**) Model for collaboration of *cenH* and *REIII* in establishing and maintaining the high fidelity MAT locus. (LEFT) During initial establishment, *cenH* heterochromatin raises the nucleation frequency at *REIII* (green arrow). A box right of *REIII* represents a putative additional nucleation element. (RIGHT) Labile *cenH*-nucleated spreading is disrupted, in part by de-stabilized nucleosomes, in a environmental perturbation or a stochastic event. *REIII* promotes reestablishment of the initial state by repressing histone turnover, limiting nucleosome loss (orange) and thus aiding spreading from *cenH* (light blue arrows, (1)), or promoting heterochromatin spreading from surrounding elements (dark blue arrows, (2)).

It is known that *REIII* recruits the HDAC Clr3 (Yamada et al., 2005), which was later shown to repress the turnover of histones (Aygün et al., 2013). This suggested the intriguing possilibty that unstable epigenetic inheritance in the absence of *REIII* is linked to elevated histone turnover. To test this idea, we adopted the Recombination Induced Tag Exchange (RITE) system (Verzijlbergen et al., 2010) to assay replication-independent turnover of H3 in *∆REIII*^HSS^ and *∆K*^HSS^ strains (Figure 6A). Tag switching (T7 for HA tag) in log phase growth was induced by administering β-estradiol concurrently with stalling replication with 15 mM hydroxyurea (HU) for 4 hr, during which time cells remain in early S phase (Figure 6—figure supplement 1). We compared the incorporation of T7 at 4 vs. 0 hr between *∆REIII*^HSS^, *∆K*^HSS-OFF^ and *∆K*^HSS-ON^ strains. First, we examined two euchromatic genes, *pyk1* on chromosome 1, and *mtd1,* which is just outside the MAT locus. H3 turnover at these regions does not differ between the strains (Figure 6B) and is highest in in the strongly expressed *pyk1* gene. We next examined sites in the MAT locus that are shared in sequence and genomic position between *∆REIII*^HSS^ and *∆K*^HSS^ (probes indicated in diagram, Figure 6B). We note this includes also *REIII*, since this locus only differs between the strains by the 14bp containing the two Atf1/Pcr1 binding sites. In contrast to euchromatic loci, we observed that *∆K*^HSS-OFF^ experiences very low or no histone turnover at MAT targets by 4 hr HU compared to *∆K*^HSS-ON^ and *∆REIII*^HSS^, which experienced levels of H3 turnover more consistent with our euchromatic controls. This in not unexpected for *∆K*^HSS-ON^, as is it effectively not heterochromatic (Figure 2E), and is consistent with previous results (Aygün et al., 2013). However, the observation that *∆REIII*^HSS^ displays H3 exchange at levels similar to *∆K*^HSS-ON^ and euchromatin suggests that it is memory, rather than heterochromatin formation itself, that requires repressed histone turnover.

**Figure 6—figure supplement 1.**
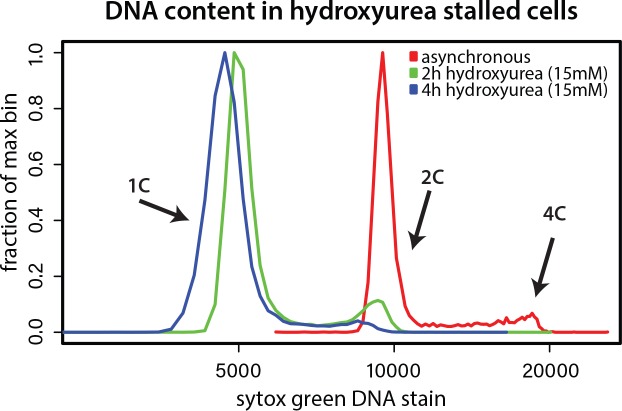
Hydroxyurea induced cell cycle arrest. Cells were grown without (asynchronous) or with 15 mM hydroxyurea for 2 or 4 hr and DNA content was determined by Sytox green staining and flow cytometry. Hydroxyurea treatment stalls cells in early S phase, evident from loss of 2 and 4C peaks.

## Discussion

The patterning of the genome into regions of activity and inactivity underlies the formation of cellular identity. In many systems heterochromatin spreading is the dominant contributor to the pattern (Schultz, 1939; Schwartz et al., 2006; Wen et al., 2009). Maintaining identity requires the capacity to ‘remember’ the positional extent of heterochromatic spreading. Yet, how precise epigenetic memory is linked to the intrinsic properties of the spreading reaction itself has remained opaque. In this work, we were able to directly measure the heterochromatin spreading reaction in single *S. pombe* cells, separate from DNA-directed events at nucleation elements, and probe its behaviors and memory characteristics. The central principle that emerges form this work is that heterochromatin spreading in fission yeast, driven predominantly by ncRNA elements, is epigenetically unstable and requires stabilization by accessory elements for high fidelity epigenetic inheritance. At the MAT locus, which carries cell identity information, a separate type of heterochromatin, independent from nc-RNA elements and dependent on the *REIII* element, safeguards epigenetic propagation by repressing histone turnover.

### ncRNA-triggered spreading is epigenetically unstable and labile in the face of perturbations

The dominant form of heterochromatin in *S. pombe,* triggered by ncRNA nucleators, leads to stochastic spreading of both silencing and H3K9 methylation that only occurs in some cells, and forms intermediate states (Figure 1 and Figure 1—figure supplement 1E, Figures 2C and 3D). This is consistent with position effect variegation in genetically disrupted systems (Elgin and Reuter, 2013; Nimmo et al., 1994). Additionally, the linear distance-dependent behavior we observe (Figure 1C) is reminiscent of the continuous spreading model in *S. cerevisiae* telomeres (Renauld et al., 1993; Talbert and Henikoff, 2006). This behavior of ncRNA spreading is not due to weak nucleation, as repressive histone marks accumulate to the same high extent at *cenH* in wild-type and *ΔREIII* and at Atf1/Pcr1 proximal region in *ΔK* cells. (Figures 1E and 2D and E). In a key result, we find ncRNA-triggered spreading to be epigenetically unstable. This is evidenced by highly dynamic behaviors over time and across generations, little discernable memory, and low resistance to chemical or environmental perturbations (Figures 3–5). Those behaviors are not necessarily predicted by the stochastic induction spreading, given that PEV in flies results in clonally inherited patches (Elgin and Reuter, 2013). This result opens the question how high fidelity can be achieved with ncRNA nucleators at loci that carry critical cell type specification information. The most likely cause for this instability is elevated and near-euchromatic levels of histone turnover (Figure 6B). This implies that while elevated histone turnover is compatible with heterochromatin formation per se, it is incompatible with epigenetic memory.

In contrast to the behavior of *ΔREIII*, *ΔK* cells, dependent on *REIII* for heterochromatin formation (Figure 2—figure supplement 2A&B), do not display stochasticity in spreading (Figures 2E and 3E), and instead repress MAT uniformly across nucleated cells in the population (Figure 2E). Under environmental perturbation, *ΔK* heterochromatin is extraordinarily resistant (Figure 4F and Figure 4—figure supplement 2A,F) and capable of high memory retention, even in the absence of the *REIII-*targeted Pcr1 protein, which attracts HDACs and Clr4/Swi6 (Jia et al., 2004; Kim et al., 2004) (Figure 5B,C). This is consistent with previously documented bistable behaviors ascribed to the overall locus (Dodd et al., 2007; Grewal and Klar, 1996). It, however, remains to be resolved whether heterochromatin in *ΔK* cells is nucleated by *REIII* and spreads outwards, or is nucleated at multiple sites, yielding apparent uniform heterochromatin formation. A *REIII* nucleated spreading model is favored by results presented here and by others (Jia et al., 2004; Wang and Moazed, 2017) that demonstrate that Atf1 and Pcr1 proteins or their binding sites are absolutely required for established of heterochromatin in *ΔK* cells, yet not for *ΔREIII* cells. However, unlike for *cenH,* where sufficiency has been clearly shown (Hall et al., 2002), we and others (Wang and Moazed, 2017) do not document significant heterochromatin formation by *REIII* when it is placed ectopically (Figure 2—figure supplement 1D). Thus, it cannot be differentiated whether the uniform heterochromatin formation in *ΔK* is the result of cooperation between different yet-to-be identified *cis*-acting elements, or a special property of *REIII-*driven spreading. Single site driven spreading of this ‘all or none’ type could be the result of looping, invoked for the polycomb system (Bantignies and Cavalli, 2011), predicted to improve spreading efficiency and memory in fission yeast (Erdel and Greene, 2016), or a unique molecular signature at *REIII.* For example, *REIII* recruits the HDAC Clr3 (Yamada et al., 2005), which promotes accumulation of the H3K9 trimethyl state, required for efficient spreading by Clr4 (Zhang et al., 2008; Al-Sady et al., 2013; Jih et al., 2017).

### *REIII* stabilizes heterochromatin spreading by repressing histone turnover

Regulation of histone turnover has been linked to epigenetic memory in fission yeast (Taneja et al., 2017) and has been previously shown to be low at wild-type MAT (Aygün et al., 2013). Hence, the high histone turnover we observe in *ΔREIII* cells results from unaided *cenH-*spreading. *REIII* recruits the HDAC Clr3 (Yamada et al., 2005), which represses histone turnover (Aygün et al., 2013). Our finding that the *ΔK*^OFF^ allele features very low histone turnover (Figure 6B), similar to the wild-type locus (Aygün et al., 2013), is thus consistent with *REIII* acting to repress histone turnover, when in a heterochromatic state. The extraordinary memory of repression we observe in *ΔK*^OFF^ likely is explained by this repressed turnover, although we should note it is possible that isolation of *ΔK*^OFF^ alleles, while consistent with the literature (for example [Grewal and Klar, 1996; Thon and Friis, 1997]), could bias the population to enhanced inheritance of repression. We speculate that reduced turnover increases retention of H3K9me3 nucleosomes, promoting methylation across nucleosomes by Clr4 via its H3K9me-dependent ‘read-write’ functionality (Al-Sady et al., 2013; Jih et al., 2017; Ragunathan et al., 2015; Zhang et al., 2008), thus facilitating re-establishment in the next generation. H3K9me3 is also directly promoted by Clr3, which is recruited to *REIII* (Yamada et al., 2005), further favoring reestablishment of methylation.

### Collaboration of ncRNA-dependent and independent mechanisms in the maintenance of MAT heterochromatin

Repression of histone turnover and resulting epigenetic stability in *ΔK* strains requires cells to first nucleate and adopt a heterochromatic state (*ΔK*^OFF^, Figure 6B). However, since *ΔK* cells only nucleate infrequently (Figure 2E), how is *REIII* able to stabilize heterochromatin in most wild-type MAT cells (Figures 2B, 3B,C and 4B)? The independent action of *cenH* and *REIII* elements cannot account for this behavior, hence they must collaborate. We propose that in the context of wild-type MAT, *cenH* stimulates *REIII* nucleation (model, Figure 6C). Recent findings indicate that Atf1/Pcr1 are present at *REIII* even in non-silenced *ΔK-*type cells (Wang and Moazed, 2017). We speculate that since Atf1/Pcr1 recruits silencing factors such as Clr4 and HDACs (Jia et al., 2004; Kim et al., 2004; Yamada et al., 2005), heterochromatin originating from *cenH* might stabilize this recruitment. This hypothesis is supported by our observation for nucleation during TSA recovery. Although *ΔK*^HSS^ cells very rarely renucleate (Figure 4D), *REIII* at the intact MAT locus must be active in most cells, as the heterochromatin reformed after erasure has much higher resistance to perturbation than that nucleated from *cenH* alone (red lines in Figure 4B and C).

Activated *REIII* in turn stabilizes the MAT locus most prominently when the heterochromatin state is perturbed. We infer this from the difference between the initial challenge and recovery from growth at high temperatures. When initially challenged, heterochromatin spreading at the wild-type MAT locus resembles that of ncRNA-nucleated heterochromatin (Figure 4—figure supplement 2A), suggesting that *REIII* or other nearby elements plays a minor role under normal circumstances at MAT. However, the heat recovery experiment suggests that changes in the *REIII*–dependent heterochromatin stabilization or assembly, not *cenH* nucleation (Figure 4—figure supplement 2B and C), takes on a major role in the accelerated recovery of the collapsed heterochromatin (Figure 4F). Thus, *REIII* is required under perturbation conditions to protect or quickly re-establish the heterochromatin state (Figure 4B,F and model Figure 6C). The relatively transient distal de-repression events experienced by wild-type MAT cells, which are much more pronounced in *ΔREIII* cells (Figure 3C and D), further points to *REIII* acting after stochastic loss of *cenH* spreading in steady state. It is possible that *REIII* does so by stabilizing existing heterochromatin via repression of histone turnover when the loss of these structures is sensed, or alternatively, that *REIII-*dependent structures expand or ‘fill-in’ the void left by collapse of *cenH-*spreading. In either case, we propose that *REIII* acts as a failsafe, ensuring the integrity, and ultimately epigenetic memory, of heterochromatin at MAT through perturbations.

In summary, we propose a model whereby the division of labor between *cenH* and *REIII* is uniquely suited for a cell type specification locus such as MAT, which requires silencing that is both robust and intergenerationally stable. ncRNA-nucleation is extremely robust but intrinsically too labile and stochastic to reliably control the cell type specification locus, thus requires support from an accessory element. The need for reliable control of cell type specification loci is shared in more complex systems. The nature of equivalent accessory elements to *REIII* and how they act in these cases remains to be determined.

## Materials and methods

### Strain construction

#### Plasmids and strain selection

Plasmids to generate constructs for genomic integration were generated by standard methods including Gibson assembly and in vivo recombination. *S. pombe* transformants were selected directly on dropout media for auxotrophic markers or onto rich media (YES) for 24 hr followed by selective media (YES + G418, YES + hygromycin or YES + nourseothricin). For all strains see Table 1.

#### Ura4 replacement method

To avoid interference of selection cassettes with heterochromatin function in our HSS, we produced ‘scarless’ genomic integrations, lacking selection markers. To do so, we marked the insertion site first with a *ura4* cassette by genomic integration and then replaced this cassette either with a XFP cassette or altered genomic sequence for site mutations. *ura4* replacements were isolated by 5-FOA counter-selection and confirmed by genomic PCR. This method was used to generate the atf/creb site deletions and sequence insertions. *ura4* was targeted to the region between Mat3M and *cenH*, specifically including the two seven base atf/creb-binding sites (s1 and s2, and [Wang and Moazed, 2017]). The entire *ura4* cassette was then replaced with a construct containing the two seven base pair deletions of s1 and s2 or a deletion of s1 with additional 700 bp of sequence from the *sib1* open-reading frame. Desired point mutations and restoration of the pre-substitution locus was confirmed by PCR and sequencing.

### Flow cytometry and FACS sorting

For standard flow cytometry experiments, cells were grown overnight in rich media (YES) and then diluted in the morning to OD = 0.1 in minimal media plus supplements (EMM complete) and grown 4–6 hr before analysis by flow cytometry. Flow cytometry was performed using Fortessa X20 Dual or LSRII instruments (Becton Dickinson, San Jose, CA). Samples sizes ranged from ~2000 to 100,000 cells depending on strain growth. Compensation was performed using cells expressing no XFPs and single-color controls expressing 1 XFP each. Compensated data was used for all downstream analyses. Fluorescence was detected for each color as described (Al-Sady et al., 2016).

For FACS sorting experiments, cells were grown overnight from OD = 0.025 in YES and then in the morning concentrated into a smaller volume to achieve a flow rate of ~5000 events/second on the cytometer. Sorting was performed using either Aria2 or Aria3u machines (Becton Dickinson). Prior to sorting cells were strained through a 35–40 μm mesh (Corning) to reduce clogs. Sorting criteria included a gate for size (forward (FSC) and side (SSC) scatter), removal of doublets, a gate for ‘green'^OFF^ (‘green’ signal within the range of an unstained control) and then gated into Low, Intermediate, High ‘orange’ signal defined by the following: Low encompassed signal overlapping that of an unstained control and High encompassed signal overlapping that of the *Δclr4* no heterochromatin control strain PAS355. Intermediate gate was set in between Low and High with about 100 fluorescence units of a gap (representing ~2% of the full range of captured fluorescence) to ensure reliable separation. The entire range of fluorescence detected was ~2.5 orders of magnitude. At least 8 × 10^6^ cells were collected for each population for Chromatin Immunoprecipitation and 2 × 10^6^ cells for RT-qPCR. Immediately after sorting, the final populations were subjected to the appropriate treatment for either Chromatin Immunoprecipitation or RT-qPCR. The R scripts for analysis is included as a text file, ‘Source Data 1’.

### Sytox green staining and cell cycle analysis

Cell cycle analyses were performed essentially as described (Knutsen et al., 2011). Briefly, cells were fixed with 70% ethanol, washed with 20 mM EDTA pH 8.0, and treated with RNaseA for 3 hr at 37°C. Immediately before analysis by flow cytometry, 2 μM Sytox Green (Invitrogen) in 20 mM EDTA pH 8.0 was used to resuspend pelleted cells. Cells were excited with a 488 nm laser and Sytox Green signal was detected with a 505-nm longpass filter and a 530/30 bandpass filter. Cell cycle analysis was performed in the FlowJo Software (Tree Star Inc, Ashland, OR) The identification of cell populations and fraction of cells in each cell cycle phase (G2, S, and G1 + M) were determined as described (Knutsen et al., 2011).

### Trichostatin A (TSA) gradient experiment

Cells were taken from fresh plates, and then grown overnight with shaking (Elmi) in 96-well plates containing 150 μL YES (Day −1). The next day (Day 0), cells were diluted into YES and measured by cytometry. At the end of Day 0, cells were passaged into YES + DMSO (0 μM TSA) or YES + 50 μM TSA overnight. The next day (Day 1), cells were diluted and grown briefly into the same pretreatment conditions and the 50 µM TSA pre-treated cells were checked for complete de-repression by flow cytometry. Complete de-repression was defined as a qualitative overlap of WT and *Δclr4* profiles, with no evidence of repression. Both 0 and 50 μM TSA pretreated cells were then diluted into a gradient of TSA of 11 two-fold dilutions from 50 μM along with a twelfth 0 μM (DMSO) point. Cells were measured after ~6 hr and then passaged into the same TSA gradient conditions to continue growth.

The next day (Day 2), cells were diluted from overnight growth into the same gradient as above, measured ~6 hr later by flow cytometry and passaged into the same gradient again overnight. The same protocol was followed for Days 3 and 4. The full experiment was performed twice at different times (biological replicate). Given the lengthy continuous growth, contamination was occasionally observed in <1% of wells. The replicate shown was chosen based on lacking contamination.

### Heat recovery experiment

Cells were taken from fresh plates, and then grown overnight with shaking (Elmi) at either 32°C or 38°C (Day-1) in 96-well plates containing 200 µL YES medium per well. In the morning, cells were diluted into 200 µL YES and grown ~6 hr at the same temperature before measurement by flow cytometry (Day 0). At the end of Day 0, all cells were all diluted again into YES and grown at 32°C. The next day (Day 1), cells were diluted from overnight growth into YES at 32°C, measured ~6 hr later by flow cytometry and passaged into the same temperature overnight. The same protocol was followed for Days 2, 3, and 4.

### Nucleation factor removal experiment

HSS strains were crossed to parent strains lacking functional nucleation factors for *REIII* (*Δpcr1*) or *cenH* (*Δdcr1 seb1-1*). Cross progeny were identified via a random spore approach by growth on selective media 2 or 3 days after plating. Absence of *pcr1* or *dcr1* open-reading frames was confirmed by PCR. Presence of *seb1-1* allele was confirmed by sequencing. Single colonies were grown in 96-well plates at 32°C containing 200 µL YES medium per well. In the morning, cells were diluted into 200 µL EMM and grown ~6 hr at the same temperature before measurement by flow cytometry. Cells were again diluted into 200 µL YES for overnight growth at 32°C and grown and measured similarly the subsequent days. For *Δdcr1* and/or *seb1-1* strains and their controls, this was continued for four days. For *Δpcr1* strains and their controls this was continued for 5 days then resulting cells were plated onto selective media and allowed to grow 48 hr at 32°C. Patches were then passaged in bulk on selective plates every 36–48 hr for 7 additional days. On the 6th day, the passaged *ΔK*^HSS^*Δpcr1* cells were additionally struck for singles. On the 8th day, patches of passaged cells and six single colonies of *ΔK*^HSS^*Δpcr1* cells were grown in 96-well plates as above and measured by flow cytometry for 5 additional days.

### Nucleosome turnover assay

Recombination Induced Tag Exchange (RITE) parent strain (HU2549) was crossed into HSS reporter strains. Resulting isolates were verified by growth on selective media. The *cdc-25ts* allele was crossed out. RITE was performed essentially as described (Audergon et al., 2015; Svensson et al., 2015) with the following exceptions. Given the labile nature of heterochromatin at elevated temperatures, replication stalling was performed with hydroxyurea as published (Aygün et al., 2013). Cells were grown to saturation overnight in YES supplemented with Hygromycin. In the morning cells were diluted to OD = 0.1 in 50 mL YES+Hygromycin and grown for 4 hr at 30°C, 225 rpm. After 4 hr of growth, 13 mL of cells were pelleted and processed for ChIP as the 0 hr time point. The remaining cells were washed twice in media devoid of Hygromycin and finally resuspended in YES supplemented with 15 mM Hydroxyurea (HU) and 1.5 μM β-Estradiol (ER) and incubated for 4 additional hours at 30°C, 225 rpm. After 4 hr incubation with HU and ER, 10 mL of cells were pelleted and processed for ChIP.

### Chromatin immunoprecipitation (ChIP) and quantification

We found that sonication of a small number of cells such as can be collected by FACS leads to a marked increase in background signal from negative control regions that was absent when ChIP was performed with larger log phase cultures (>50 × 10^6^ cells). To address this, ChIP in Figure 1E was performed on each of the FACS sorted populations with the addition of 42 × 10^6^ formaldehyde fixed cells of *S. cerevisiae* W303 strain as a carrier. ChIP in Figure 2D was performed with 15 × 10^6^ cells of each fission yeast strain and 50 × 10^6^ additional W303. ChIPs for Figure 2E and Figure 2—figure supplement 2B were performed with 80 × 10^6^ cells and no added W303. ChIPs for Figure 6B were performed with no added W303. ChIP was additionally performed on a sample of W303 alone, which only produced signal equivalent to background. *S. pombe* ChIP samples and W303 cells were fixed and pre-processed for ChIP separately, then mixed together immediately prior to lysis. Cells were cross-linked and lysates prepared for ChIP as described (Canzio et al., 2011) with the following exceptions: After lysis, the chromatin fraction was resuspended in 350 μL lysis buffer and sonication performed using a Diagenode Bioruptor Pico machine at 4°C, with 16–20 rounds of 30 s ON, 30 s rest. ChIP was essentially as described, with the total lysate split into 2–6 equal volumes (after ~8% set aside as input fraction) and ChIP performed in 600–800 μL per sample. Two or three technical replicates were performed across experiments. 1 μL of each of the following antibodies was added per ChIP replicate: anti-H3K9me2 (Abcam ab1220); anti-H3K4me3 (Active Motif 39159); anti-H3K9me3 (Millipore 07–442); anti-T7 (Novagen 69522–3). ChIP samples were agitated on a Nutator overnight at 4°C. Immune complexes were collected for 3 hr with 15–20 μL washed protein A Dynabead slurry (Invitrogen). Washing and downstream processing steps were essentially as described, except ‘wash buffer’ wash was performed once. Samples were purified using a Machery-Nagel PCR purification kit and NTB buffer for SDS containing samples. DNAs were quantified by RT-qPCR (see below). Enrichments were calculated as follows: For Figures 1E, 2D and E IP/input values for amplicons of interest were calculated and normalized to the IP/Input values for positive controls for each antibody, *dh* for H3K9me2 and H3K9me3 and the actin promoter for H3K4me3. For Figure 2—figure supplement 2B, ChIP signal was normalized to signal from a matched background *Δclr4* strain. For Figure 6B IP/input values for the 4 hr time points were normalized to the IP/input values for the 0 hr time point.

### RNA extraction and mRNA quantification

After sorting, samples were spun at 5000xg, supernatant decanted, and pellets flash frozen in liquid nitrogen and stored at −80°C. For the *Δclr4* strain PAS335, cells were grown into log phase and then cell pellets were isolated in the same fashion. Total RNA was extracted in technical duplicates from the same cell pellets using the ‘MasterPure- Yeast RNA Purification Kit’ (Epicentre), including a 30 min DNAse treatment step post-RNA isolation. Reverse Transcription was performed with SuperScript III RT (Invitrogen), using the supplied protocol and 1.5–2 μg of RNA and an oligo dT primer. Following cDNA synthesis the reaction was treated with RNAse H (New England Biolabs). cDNA samples were quantified by RT-qPCR. For each sorted sample, mKO2 cDNA values were normalized to actin and then divided by the max value calculated similarly from PAS355 (*Δclr4*).

### RT-qPCR

Real-time quantitative PCR was performed using a BioRad CFX-384 machine. 15 μL reactions were prepared, each containing 7.5 μL of Applied Biosystems SYBR Select Master Mix, 4.5 μL 3.3M betaine, 1.2 μL of 2.5 μM oligo mix, 0.8 μL water, and 1 μL template. The thermocycler protocol was: 2 min at 50°C then 2 min at 95°C followed by 40 cycles of 15 s at 95°C and then 1 min at 60°C followed by a plate read. Lastly a melt curve was generated. Standards were generated with five fold dilutions of genomic DNA containing templates for all PCR products.

### Single-cell microscopy

Single cells of strains PAS 387, 389, 391 and 244 (see Table 1; E2Crimson under *act1* promoter) were captured in microfluidic devices as described (Spivey et al., 2017). Multi-channel fission yeast lifespan microdissectors (multFYLM) contained six independent devices (channels), each of which is capable of capturing up to 392 cells (https://bio-protocol.org/e2783). In brief, the devices were cast in polydimethylsiloxane (PDMS, Sylgard 184, Dow Corning) using conventional soft lithography methods. Master structures were fabricated from P-doped silicon wafers (ID#452, University Wafers) and SU-8 photoresists 3005 and 2010 (Microchem, Westborough, MA). MultFYLMs were cleaned and adhered to glass coverslips (48 × 65 mm #1, Gold Seal), and then connected to syringes (60 mL, Becton-Dickson) containing YES 225 liquid media (Sunrise Science) via PFA tubing and microfluidic fittings (IDEX Health and Science). The multFYLM was maintained at 30˚C in a custom staged-mounted environmental chamber on an inverted microscope (Eclipse Ti, Nikon) equipped with NIS Elements software (Nikon), a 60X air objective (CFI Plan Apo λ, 0.95 NA, Nikon) fitted with an objective heater (Bioptechs), a motorized stage (Proscan III, Prior), and an active feedback-based focusing system (Perfect Focus System, Nikon). An LED lamp (Sola II, Lumencorp) and a scientific-grade CMOS camera (Zyla 5.5, Andor) were used for fluorescent imaging. Multi-color fluorescent imaging of sfGFP, mKO2 and E2Crimson fluorophores was carried out by alternating between three filter sets mounted in a computer-controlled filter ring (Chroma 49002, 49010 and 49015, respectively). To help with the semi-automated cell identification, each channel was imaged every ten minutes via brightfield imaging (100 ms exposure, both in focus and 4 μm below the focal plane). Fluorescent images of each of the three fluorophores were taken every 30 min (150 ms exposure). This illumination scheme was well below the phototoxicity limit, as described previously (Al-Sady et al., 2016). Raw images were saved as uncompressed 16 bit ND2 files and further analyzed using a custom-written image analysis pipeline (see below).

Cells were grown overnight (30°C with 225 rpm shaking) to saturation in YES media, then diluted in YES to an optical density at 600 nm (OD_600_) of 0.1 and allowed to grow for approximately 5 hr to reach an OD_600_ of 0.5. Cells (60 μL at OD 0.5 in YES + 2% Bovine Serum Albumin, BSA) were loaded at the entry port of the multFYLM. After cells entered individual channels, media lines were reattached and YES media was pumped through on a pulse cycle (14 min: 5 µLmin^−1^, 1 min: 55 µLmin^−1^) for the entire experiment. This flow regime was optimized to flush out occasional cell clumps that grew at the device inlets and other fluidic interfaces. Four genotypes were imaged simultaneously for 60 hr in each channel of a multFYLM device to ensure identical imaging and growth conditions. In all cases, we only analyze the innermost cell, which was the oldest cell pole (see below). Cells that were ejected or died within the first 12 hr after loading were not included in the downstream analysis.

### Single-cell image analysis

Single-cell imaging data was processed using an updated version of the custom-written FYLM Critic analysis package (Spivey et al., 2017). The source-code is available via GitHub (https://github.com/finkelsteinlab/fylm; Rybarski et al., 2015; copy archived at https://github.com/elifesciences-publications/fylm). FYLM Critic performs the following automated processing on the raw images: (1) rotation; (2) jitter removal via a cross-correlation algorithm; and (3) generation of kymograph and individual cell images. The latter were used to create videos of individual cells in Fiji (Schindelin et al., 2012). The final outputs of FYLM critic are the position and contour of each dividing cell, as well as the time-dependent fluorescence intensities for each cell. These fluorescence intensities are obtained by averaging the intensity across all pixels that fall within the cell volume, as defined by the bright-field images. This normalization also ensures that the fluorescence intensity is corrected for the size of the rapidly dividing cells. Time-dependent fluorescent intensities were analyzed via custom-written MATLAB scripts (version 2017a Mathworks, available upon request). Background fluorescence from the PDMS device was subtracted using catch tubes that did not receive a cell. The maximum heterochromatin reporter (GFP, mKO2) fluorescence intensity was calculated using *∆clr4* cells in the same reporter construct background. To control for expression variation across the cell cycle, the fluorescence from heterochromatin reporters was also reported as a ratio of the control fluorophore, E2Crimson. Similarly, cells fluorescing in the clamp channel were removed from analysis for MAT-locus-derived strains (see Appendix 1-Supplemental Materials and methods).

Single-cell images generated by the FYLM Critic analysis were compiled into stacked movies using Fiji. Images in bright field and for each color channel were processed separately in batch and then later combined into a vertical stack. For each channel, 0.2% of pixels were allowed to become saturated and pixel values were normalized to the maximum range for the whole sequence in that channel. For bright field, every third image was included to match the imaging frequency of the fluorescent channels. Movies were edited for length to include contiguous imaging sequences without loss of focus and for size to remove non-cellular debris and cells from the opposite side of the channel that entered the field of view. After combining all color channels and bright field, the brightness and contrast were increased for cell 407 to match the red channel brightness of the other strains. Image sequences were saved as uncompressed. avi files with a rate of 15 frames/s.

**Table 1.** Yeast strains used in this study.

Strain	Genotype
PAS075	Locus2*::ade6p::3xE2C:hygMX* at Locus2 (between SPBC1711.11 andSPBC1711.12)
PM03	Wild-type strain: h(+); ura4-D18; leu1-32; ade6-M216; his7-366
PM1035	*ura4::natMX:dh* fragment 1, *clr4::KAN* as in Marina et al. (2013)
PAS111	*ura4::natMX:dh*:*ade6p:*SF-GFP, *ade6p:*mKO2 7 kb, *ade6p:*3xE2C: *hygMX* at Locus2
PAS112	*ura4::natMX:dh*:*ade6p:*SF-GFP, *ade6p:*mKO2 7 kb, *ade6p:*3xE2C: *hygMX* at Locus2; *clr4::kanMX*
PAS133	*ura4::natMX:dh*:*ade6p:*SF-GFP, *ade6p:*mKO2 1 kb, *ade6p:*3xE2C: *hygMX* at Locus2; *clr4::kanMX*
PAS134	*ura4::natMX:dh*:*ade6p:*SF-GFP, *ade6p:*mKO2 1 kb, *ade6p::*3xE2C*: hygMX* at Locus2
PAS135	*ura4::natMX:dh*:*ade6p:*SF-GFP, *ade6p:*mKO2 3 kb, *ade6p::*3xE2C*: hygMX* at Locus2; *clr4::kanMX*
PAS136	*ura4::natMX:dh*:*ade6p:*SF-GFP, *ade6p:*mKO2 3 kb, *ade6p::*3xE2C: *hygMX* at Locus2
PAS141	*ura4::natMX:dh*:*ade6p:*SF-GFP, *ade6p:*mKO2 5 kb, *ade6p::*3xE2C: *hygMX* at Locus2
PAS142	*ura4::natMX:dh*:*ade6p:* SF-GFP, *ade6p:*mKO2 5 kb; *ade6p::*3xE2C*: hygMX* at Locus2; *clr4::kanMX*
PAS192	Δ*K*::*ade6p:*mKO2; *ade6p:* SF-GFP between *REIII* and mat3M; *ade6p:*3xE2C*: hygMX* at Locus2, h(-)
PAS193	Δ*K*::*ade6p:*mKO2; *ade6p:*SF-GFP between *REIII* and mat3M; *ade6p*:3xE2C*: hygMX* at Locus2; *clr4::kanMX,* h(-)
PAS214	Δ*K*::*ade6p:*mKO2:*ura4t*; *mat3m(Eco*RV):: *ade6p:*SF-GFP; *ade6p*:3xE2C*: hygMX* at Locus2; *clr4::kanMX,* h(-)
PAS215	Δ*K*::*ura4t:*mKO2:*ade6p*; *mat3m(Eco*RV):: *ade6p:*SF-GFP; *ade6p*:3xE2C*: hygMX* at Locus2; *clr4::kanMX,* h(-)
PAS216	*cenH*::*ade6p:*SF-GFP (Kint2); *mat3m(Eco*RV):: *ade6p:*mKO2; *ade6p:*3xE2C: *hygMX* at Locus2; *clr4::kanMX,* h90
PAS217	*cenH*: *ade6p:*SF-GFP (Kint2); *mat3m(Eco*RV):: *ade6p:*mKO2; *ade6p:*3xE2C: *hygMX* at Locus2, h90
PAS218	*cenH*::*ade6p:*mKO2 (Kint2); *mat3m(Eco*RV):: *ade6p:*SF-GFP; *ade6p:*3xE2C*: hygMX* at Locus2; in *clr4::kanMX,* h90
PAS219	*cenH*: *ade6p:*mKO2 (Kint2); *mat3m(Eco*RV):: *ade6p:SF-GFP*; *ade6p:3xE2C: hygMX* at Locus2, h90
PAS231	*ura4::natMX:dh:ade6p:*SF-GFP, *ade6p:*mKO2 3 kb, *leu1*::*ade6p:*3xE2C: *hygMX*
PAS237	*ura4::natMX:dh*:*ade6p:*SF-GFP, *ade6p:*mKO2 3 kb, *act1p::q*xE2C*: hygMX* at Locus2; *clr4::kanMX*
PAS243	*ura4::natMX:dh*:*ade6p:*SF-GFP, *ade6p:*mKO2 3 kb, *act1p::*1xE2C*: hygMX* at Locus2; *clr4::kanMX*
PAS244	*ura4::natMX:dh*:*ade6p:*SF-GFP, *ade6p:*mKO2 3 kb, *act1p::*1xE2C: *hygMX* at Locus2
PAS264	*cenH*:: *ade6p:*SF-GFP (Kint2); *mat3m(Eco*RV):: *ade6p:*mKO2; *ade6p*:3xE2C*: hygMX* at Locus2, *pcr1::kanMX,* h90
PAS268	*ΔK*:: *ade6p:*mKO2; *ade6p:*SF-GFP between REIII and mat3M; *ade6p:*3xE2C*: hygMX* at Locus2, *REII::LEU2,* h(-)
PAS269	*ΔK*:: *ade6p:*mKO2; *ade6p:*SF-GFP between REIII and mat3M; *ade6p:*3xE2C*:hygMX* at Locus2; *clr4::kanMX, REII::LEU2,* h(-)
PAS331	*cenH*:: *ade6p:*SF-GFP (Kint2); *mat3m(Eco*RV):: *ade6p:*mKO2; *ade6p:*3xE2C:*hygMX* at Locus2; Δ*REIII*::REIII(Δs1, Δs2) in *clr4::kanMX,* h90
PAS332	*cenH*:: *ade6p:*SF-GFP (Kint2); *mat3m(Eco*RV):: *ade6p:*mKO2; *ade6p:*3xE2C:*hygMX* at Locus2; Δ*REIII*::REIII(Δs1, Δs2), h90
PAS348	*ura4::hygMX:REIII*:*ade6p:*SF-GFP; *ade6p:*mKO2 5 kb, *ade6p:*3xE2C:*natMX* at Locus2
PAS350	*ura4::hygMX:REIII*:*ade6p:*SF-GFP; *ade6p:*mKO2 5 kb, *ade6p:*3xE2C:*natMX* at Locus2 *dcr1::kanMX*
PAS355	*ura4::natMX:dh*:*ade6p:*SF-GFP, *ade6p:*mKO2 3 kb, *leu1*::*ade6p:*3xE2C:*hygMX*; *clr4::kanMX*
PAS385	*ΔK*:: *ade6p:*mKO2; *ade6p:*SF-GFP between *REIII* and mat3M; *act1p*:1xE2C*:hygMX* at Locus2; *clr4::kanMX,* h(-)
PAS387	Δ*K*:: *ade6p:*mKO2; *ade6p:* SF-GFP between *REIII* and mat3M; *act1p:*1xE2C*: hygMX* at Locus2, h(-)
PAS388	*cenH:: ade6p:*SF-GFP (Kint2); *mat3m(Eco*RV):: *ade6p:*mKO2; *ura4* at Locus2; *leu1*::*act1p:*1xE2C:*hygMX*, *clr4::kanMX,* h90
PAS389	*cenH:: ade6p:*SF-GFP (Kint2); *mat3m(Eco*RV):: *ade6p:*mKO2; *ura4* at Locus2; *leu1*::*act1p:*1xE2C:*hygMX*, h90
PAS390	*cenH:: ade6p:*SF-GFP (Kint2); *mat3m(Eco*RV):: *ade6p:*mKO2; *ura4* at Locus2; Δ*REIII*::REIII(Δs1, Δs2), *leu1*::*act1p:*1xE2C:*hygMX*, in *clr4::kanMX,* h90
PAS391	*cenH:: ade6p:*SF-GFP (Kint2); *mat3m(Eco*RV):: *ade6p:*mKO2; *ura4* at Locus2; Δ*REIII*::REIII(Δs1, Δs2)*, leu1*::*act1p:*1xE2C:*hygMX*, h90
PAS398	*his1::natMX:dh*:*ade6p:*mKO2*; ade6p:*SF-GFP 3 kb, *ade6p::*3xE2C*:hygMX* at Locus2, *clr4::kanMX, ura4::phyB*.
PAS399	*his1::natMX:dh*:*ade6p:*mKO2*; ade6p:*SF-GFP 3 kb, *ade6p::*3xE2C*:hygMX* at Locus2, *ura4::phyB*.
PAS410	Δ*K*:: *ade6p:*mKO2; *ade6p:* SF-GFP between *REIII* and mat3M; *ade6p::*3xE2C*:hygMX* at Locus2, *natMX:clr4+,* h(-); ‘OFF’ allele
PAS411	Δ*K*:: *ade6p:*mKO2; *ade6p:* SF-GFP between *REIII* and mat3M; *ade6p::*3xE2C*:hygMX* at Locus2, *natMX:clr4+,* h(-); ‘ON’ allele
PAS464	Δ*K*::*ade6p:*mKO2:*ura4t*; *mat3m(Eco*RV):: *ade6p:*SF-GFP; *ade6p*:3xE2C*: hygMX* at Locus2; *natMX:clr4+,* h(-)
PAS465	Δ*K*::*ura4t:*mKO2:*ade6p*; *mat3m(Eco*RV):: *ade6p:*SF-GFP; *ade6p*:3xE2C*: hygMX* at Locus2; *natMX:clr4+,* h(-)
PAS473	Δ*K*:: *ade6p:*mKO2; *ade6p:* SF-GFP between *REIII* and mat3M; 700 bp *sib1* ORF between REIII-s1 and mKO2; *ade6p:*3xE2C*: hygMX* at Locus2, *clr4::kanMX,* h(-);
PAS474	Δ*K*:: *ade6p:*mKO2; *ade6p:* SF-GFP between *REIII* and mat3M; Δ*REIII*::REIII(Δs1, Δs2), *ade6p:*3xE2C*: hygMX* at Locus2, *clr4::kanMX,* h(-);
PAS478	Δ*K*:: *ade6p:*mKO2; *ade6p:* SF-GFP between *REIII* and mat3M; 700 bp *sib1* ORF between REIII-s1 and mKO2 *ade6p:*3xE2C*: hygMX*, *natMX:clr4+,* h(-);
PAS482	Δ*K*::*ade6p:*mKO2; *ade6p:* SF-GFP between *REIII* and mat3M; *ade6p:*3xE2C*: hygMX* at Locus2, h(-); ‘OFF’ allele
PAS483	Δ*K*:: *ade6p:*mKO2; *ade6p:* SF-GFP between *REIII* and mat3M; Δ*REIII*::REIII(Δs1, Δs2), *ade6p:*3xE2C*: hygMX* at Locus2, *natMX:clr4+,* h(-);
PAS496	*cenH*:: *ade6p:*SF-GFP (Kint2); *mat3m(Eco*RV):: *ade6p:*mKO2; *ade6p:*3xE2C:*hygMX* at Locus2; Δ*REIII*::REIII(Δs1, Δs2), *ars1::prad15:cre-EBD:LEU2*; *h3.2:lox:HA:hygMX:lox:T7;* h90
PAS497	Δ*K*::*ade6p:*mKO2; *ade6p:* SF-GFP between *REIII* and mat3M; *ade6p:*3xE2C: *hygMX* at Locus2; *ars1::prad15:cre-EBD:LEU2*; *h3.2:lox:HA:hygMX:lox:T7;* ‘OFF’ allele, h(-)
PAS498	Δ*K*::*ade6p:*mKO2; *ade6p:* SF-GFP between *REIII* and mat3M; *ade6p:*3xE2C*: hygMX* at Locus2; *ars1::prad15:cre-EBD:LEU2*; *h3.2:lox:HA:hygMX:lox:T7;* ‘ON’ allele; h(-)
PAS508	Δ*K*::*ade6p:*mKO2; *ade6p:* SF-GFP between *REIII* and mat3M; *ade6p:*3xE2C*: hygMX* at Locus2, ‘OFF’ allele; *pcr1::kanMX*
PAS510	*cenH*:: *ade6p:*SF-GFP (Kint2); *mat3m(Eco*RV):: *ade6p:*mKO2; *ade6p:*3xE2C:*hygMX* at Locus2; Δ*REIII*::REIII(Δs1, Δs2)*, pcr1::kanMX*
PAS514	Δ*K*::*ade6p:*mKO2; *ade6p:* SF-GFP between *REIII* and mat3M; *ade6p:*3xE2C*: hygMX* at Locus2, ‘OFF’ allele; *dcr1::kanMX*; *seb1-1:natMX*
PAS515	*cenH*:: *ade6p:*SF-GFP (Kint2); *mat3m(Eco*RV):: *ade6p:*mKO2; *ade6p:*3xE2C:*hygMX* at Locus2; Δ*REIII*::REIII(Δs1, Δs2)*, seb1-1:natMX*

## Additional files

Source code 1. R scripts for flow cytometry analysis

Transparent reporting form

### Data availability

All data generated or analysed during this study are included in the manuscript and supporting files. The code for analyzing live cell data is included in the submission. All reagents generated in this work are available upon request.

## Appendix 1

### Supplemental discussion

10.7554/eLife.32948.025 Alternative formal model for *cenH* and *REIII* interaction In the main discussion, we propose that *cenH* stimulates *REIII* nucleation to account for the high proportion of the spreading in wild-type MAT cells. Another formal possibility remains that non-nucleated, Atf1/Pcr1-bound, *REIII* raises *cenH* spreading efficiency. In the *ΔREIII* strain, Atf1/Pcr1 binding sites have been fully deleted, it is possible that when bound but not in a heterochromatin state, Atf1/Pcr1 acts to encourage more efficient spreading out of *cenH,* possibly by directing the locus to a more spreading competent location or via its recruitment of HDACs.

### Supplemental materials and methods

Basic three color HSS analysis in R Please note the relevant R scripts can be found in ‘Source Data 1’. Below a description of the method. Reading in the data Standard flow cytometry data files (.fcs) were read in with the R package flowCORE (Bioconductor, https://www.bioconductor.org/packages/release/bioc/html/flowCore.html). Isolating successfully nucleated cells (Figures 1 and 2) A strain closely matched in genetic background to HSS strains but containing no XFPs was analyzed under the same flow cytometry conditions in each experiment. This ‘unstained’ control was gated for cell size in the same manner as analysis strains and both the median fluorescence and standard deviation determined in green or orange channels (the signal from the *ade6p:*SF-GFP, *ade6p:*mKO2 or *ade6p:*E2C transcriptional units is referred to here as ‘green’, ‘orange’ or ‘red’). A nucleation cutoff was set for a value corresponding to the median of fluorescence units in the clamp channel plus two standard deviations from this unstained control. Only cells with signal less than this value were considered for post nucleation analysis. Normalizing to max fluorescence values from Δclr4 strains (Figures 1 and 2) Max values in *Δclr4* strains were determined by calculating the median raw fluorescence in each color channel after gating for cell size. For each cell of each strain for analysis, the signal in each channel was divided by this max value for the corresponding *Δclr4* strain. Normalizing to constitutive red signal For each cell of each strain, the ‘green’ and ‘orange’ values were divided by the ‘red’ value. The red- and *Δclr4-*normalized values range from 0 to ~1.5, where one corresponds to the *Δclr4* (max) value. As this value is derived from the mean of a cell distribution, with *Δclr4* cells falling above and below the mean, we plotted out to 1.5 to capture cells with ratio values above 1.0. Hexbin (2-D Histogram) Analysis Normalized ‘green’ and ‘orange’ values (without any nucleation cutoff applied) were plotted as hexbin (or 2-D histogram) plot where density is color-coded in grayscale. Data points within x = 0–1.5 and y=0–1.5 were isolated and a hexbin plot was generated using n = 40–45 bins along each axis. Hexbin plots were generated using the R package hexbin (https://CRAN.R-project.org/package=hexbin) Spreading Analysis with Nucleation Clamp Cells within the FSC/SSC gate and with signal below the cutoff value for the nucleation color were plotted in a 1-D histogram with n = 50–200 bins where the points in the middle of the histogram bins were plotted connected by a line. Modifications for Figure 4 For every TSA concentration and pre-condition (no TSA or 50 μM) each strain was normalized to the median fluorescence of a *Δclr4* strain grown under the same treatment. The cytometer settings were adjusted so an unstained control had mean fluorescence in the green channel of 10^2^. A ‘green’ cutoff for nucleation was assigned to be 400 fluorescence units. ‘orange’ cutoff values for each analysis strain were generated by determining mean and two standard deviations in PAS217 at 0 μM TSA from the no TSA precondition normalized to the appropriate *Δclr4* strain for each analysis strain. Previous analysis demonstrated both colors in PAS217 to be fully repressed, as evident in the mean for each channel. For Figure 4B,C we calculated at each TSA concentration the fraction of cells with green signal below the ‘green’ cutoff that have an orange signal below the ‘orange’ cutoff. These values were normalized to the fraction calculated for cells of that strain at 0 μM TSA from the no TSA precondition. For Figure 4D we calculated at each concentration of TSA the fraction of all cells with orange signal below the ‘orange’ cutoff, because in the 50 μM TSA pre-condition, insufficient cells exist that are below the cutoff for ‘green’ to perform above analysis. These values were normalized to the fraction calculated for cells of that strain at 0 μM TSA from the no TSA precondition. In Figure 4F, for each strain at each temperature, we calculated the fraction of cells that had ‘green’ signal less than the ‘green’ cutoff AND ‘orange’ signal less than the ‘orange’ cutoff. These values were normalized to the fraction calculated for cells of that strain at 32°C. The cutoff values were based on calculations of two standard deviations from the mean of a red only strain normalized to *Δclr4* controls. For ‘green’ and ‘orange’ these values were approximately 0.35 and 0.4 respectively so these values were used for all strains to standardize the analysis. Modifications for Figure 5 For Figure 5B,E for each strain we calculated at each time point the fraction of cells that had ‘green’ signal less than the ‘green’ cutoff AND ‘orange’ signal less than the ‘orange’ cutoff. The cutoff values for ‘green’ and ‘orange’ were each 0.4 as in Figure 4—figure supplement 2A. Samples in Figure 5B,C were gated for size because we were able to measure 10^5^ cells as the strains grew well. Samples in Figure 5E,F were not gated for size because cell growth was poor in *seb1-*1 isolates. In Figure 5C 5000 cells were plotted in a scatter plot with point transparency. In Figure 5D ~1400 cells were plotted in a scatter plot with point transparency. For Figure 5F ~2500 cells were plotted in a scatter plot with point transparency. Data in Figure 5 Supplement A were plotted as in Figure 5C and in Figure 5 Supplement B as in Figure 5F. Fitting t_1/2_ for 38°C spreading recovery To derive a t_1/2_, which is the time required to recover to 50% of the full spreading observed at 32°C, we fit the data to a simple sigmoidal dose-response variable slope model:

$$fractionallcellswithfullspreading=Bottom+\frac{t^{n}*(Top-Bottom)}{t^{n}+t_{1/2}^{n}}$$

where Bottom is the starting fraction of cells with full spreading at t = 0, Top = 1, t is time in hrs. *n* represents a Hillslope. In Figure 4—figure supplement 2A, we calculated the fraction of cells that had ‘green’ signal less than the ‘green’ cutoff AND ‘orange’ signal less than the ‘orange’ cutoff. The cutoff values were based on two standard deviations from the mean of a red only strain normalized to *Δclr4* controls. For green and orange these values were approximately 0.4 and 0.4 respectively so these values were used for all strains to standardize the analysis. These cells were gated for FSC and SSC to isolate live cells because at the highest temperature many cells had died. In Figure 4—figure supplement 2B,C the 1-D histograms for all ‘green’ values were calculated as above with the modification that no size gate was called. In Figure 4—figure supplement 2C–E the 1-D histograms and hexbin plot insets were calculated as above with the modification that no size gate was called and the ‘green’ cutoff values were generated as in Figure 4F. FYLM data analysis Initial data calculations Loss of focus was identified by red fluorescence measurements below a cutoff of one standard deviation from the mean of all collected values of red for all cells. This loss of focus data was removed from analysis. Background fluorescence from the PDMS device at each time point was then subtracted using catch tubes that did not receive a cell. For each MAT strain, its matched *Δclr4* strain was also imaged and a mean and standard deviation were calculated. In each strain cells were normalized to this mean *Δclr4* value (defined as ‘max’) and to their own red values as in the flow cytometry data analysis. An ON gate (used in Figure 3B) for cells that reached maximal de-repression was calculated for each strain from the *Δclr4* strain mean less three standard deviations. Calculating nucleation gates As seen by flow cytometry and visual inspection of collected movies, the vast majority of MAT locus cells have a repressed nucleation reporter (‘green’), which allowed us to formulate a very strict nucleation cutoff from the collected FYLM data itself. This cutoff was the mean plus two standard deviations of all measured values of all cells. Only cells that maintained a green signal less than this cutoff for their entire measured lifespan were included for further analysis in Figure 3. We did not apply this nucleation gate to the ectopic strain, as few cells maintained ‘green’ tightly repressed throughout their measured lifespan. Instead, we show all the cells in the traces plot and highlight in grey example cells that remain nucleated or mostly nucleated throughout their measure lifespans. Rescaling orange to fix negative values Due to background subtraction (see above) a significant fraction of cells experienced negative values for their adjusted fluorescence in the orange channel. To account for this, the data for the MAT strains in Figure 3 was rescaled by determining the lowest value measured (minVal) and adding the difference between that value and 0 to every time point of every cell. Values for all timepoints were then divided by 1+minVal to rescale back to 1 = max. The ectopic strain was not rescaled. Data smoothing For trace plots and heatmaps data was smoothed using a moving average of the two-nearest neighbor data points before and after. This number was chosen as it represents the timeframe of one cell division and is on the timescale of the expression and maturation of the XFPs used in these strains. Traces Individual cell traces represent the red normalized and smoothed, green and orange fluorescence data plotted over time. Traces begin and end at whatever time a cell entered or exited the channel or died. Therefore, not all traces start at x = 0 or end at x = 60. Curated example cells were also plotted as overlays using gray lines. For these curated cells similar trace plots for orange divided by green was plotted in Figure 3—figure supplement 2C-BOTTOM, D-BOTTOM, and F. Heatmaps Points with red values greater than 50% of the mean were removed. For cells that remained nucleated throughout their measured lifespan, up to 36 hr of measurements of normalized green or orange was plotted as a heat map from blue (0) to yellow (1) for 30 of the longest imaged cells. White gaps indicate transient loss of focus of less than 2 hr (four time points). Heatmaps were no longer plotted for any cell that had a loss of focus event for more than four time points. X-Y fluorescence line plots For the cells in each behavior category (Figure 3—figure supplement 1A), an X-Y plot was generated that plots the unsmoothed, red-normalized ‘orange’ vs ‘green’ values for each cell as a line. Values were normalized to the mean in ‘green’ and ‘orange’ from a matched background *Δclr4* strain to set the value of 1, while background fluorescence from empty channels were set to the value of 0. Line segments are colored from blue to pink generated based on the measurement time of that cell while the behavior was observed. The first measured point is represented in blue, the last in pink, and the color values in between are divided into increments by the total measured time for the cell. X-Y fluorescence dot plots For one or two selected cells per strain an X-Y fluorescence dot plot was generated that plots the smoothed ‘orange’ vs ‘green’ values for every third time point imaged over its entire measured lifespan. Points are colored in a grayscale that is generated based on the measured lifespan of that cell. The first measured point is represented in black, the last in white and the number of remaining points set by the total measured lifespan of the cell. In Figure 3—figure supplement 1B X-Y fluorescence dot plots were generated for four example category 1 cells. In this figure, time is colored from blue to pink as in the X-Y line plots and points represent 30 min intervals beginning when both colors are fully expressed and ending when both reach a local minimum. Cell fate pie charts The number of measured time points for each cell was determined and converted to hours (one fluorescence image every 30 min). Cells were then binned into lifetime groups of < 12 hr, 12–36 hr, or > 36 hr. Within these bins the cells were separated based on whether they died as annotated in FYLM Critic or if their traces were cut short due to late entry into or ejection from the catch channel.
