# Supplementary material for: Noncoding RNA-nucleated heterochromatin spreading is intrinsically labile and requires accessory elements for epigenetic stability
Source: eLife. 2018 Jul 18;7:e32948. doi: 10.7554/eLife.32948 (PMC6070336; doi:10.7554/eLife.32948)
Supplement: Peer review history

# Decision letter

**DOI:** 10.7554/eLife.32948.029

**Affiliations:** Institut CurieFrance

In the interests of transparency, eLife includes the editorial decision letter and accompanying author responses. A lightly edited version of the letter sent to the authors after peer review is shown, indicating the most substantive concerns; minor comments are not usually included.

[Editors’ note: this article was originally rejected after discussions between the reviewers, but the authors were invited to resubmit after an appeal against the decision.]

Thank you for submitting your work entitled "A memory element imposes epigenetic behavior on intrinsically labile RNAi-induced heterochromatin spread" for consideration by *eLife*. Your article has been reviewed by a Senior Editor, a Reviewing Editor, and three reviewers.. The reviewers have opted to remain anonymous.

Our decision has been reached after consultation between the reviewers. Based on these discussions and the individual reviews below, we regret to inform you that your work will not be considered further for publication in *eLife*. The overall conclusion is that this work is potentially of interest and could be worthy of publication in *eLife* in the future. However, in its current version it would require too much work and thus it cannot be deemed appropriate for revision within a reasonable time frame.

If you can incorporate the important critiques and suggestions made by the reviewers below, you are encouraged to resubmit your manuscript to *eLife*. In that case, the resubmitted manuscript would be considered a "new" submission and go through the same 2-stage process. However, unlike a true new submission, it would be typically seen by the editor and if needed the original reviewers.

Summary:

In this manuscript the authors set out to explore nature of heterochromatin spreading in fission yeast using a system that quantitatively measures heterochromatic silencing in living cells and follows the spreading reaction using fluorescent reporters. They report that spread of silencing from RNAi-nucleated elements is stochastic and multimodal and fluctuates over time. This is also supported by recent work by Obersriebnig et al. The authors also find another form of spreading, nucleated by the cis-acting element *REIII*, that they term deterministic, and that appears to show greater memory and fidelity in local spreading, being better buffered. This finding is novel. However, in the absence of a mechanism for *REIII* -mediated spreading, the work remains preliminary and there may be other interpretations of the data presented, as highlighted in the reviewers' comments. In summary, the authors do not really address the questions of heterochromatin spreading fidelity or memory as they set out to. In particular, evidence for actual "spreading" of heterochromatin from the so-called nucleation site and the genes assayed for silencing at some distance from it, is lacking. Based on the reviewers' comments, the overall conclusion is that this work is potentially of great interest and could be worthy of publication in *eLife* in the future. However, in its current version it would require too much work and thus it cannot be deemed appropriate for revision within a reasonable time frame.

Reviewer #1:

The fundamental problem conceptual with this paper is that the experiments are designed and interpreted in the context of a model in which spreading is assumed to be a process that emanates from a regulatory site and progressively reaches a detector site or sites. There is no evidence that "spreading" of heterochromatin of this type actually occurs in *S. pombe* or its relative *S. cerevisiae*. The crucial missing datum for all spreading models is some kind of time resolution between events at the cis-regulatory site, called the nucleation site in this study and others, and the genes at a distance that fall under the control of this regulatory site. Moreover, the way the experiments are conducted, for example by starting with cells and colonies that are preselected to be nucleated (green off) prevents the authors from the opportunity to observe the very data that could falsify the hypothesis (i.e. observing cells in which green is on and orange is off.)

The second major problem in the paper is the assertion of being able to distinguish between stochastic spreading and deterministic spreading. Let's for the moment imagine that spreading actually occurs. The distinction the authors make between stochastic and deterministic spreading is based upon the range of expression of the detector genes that are positioned to be under the influence of the nucleation site. If that range of expression is narrow, spreading is defined to be deterministic. If it is broad, spreading is defined to be stochastic. The competing model, never mentioned nor tested, is that heterochromatin formed in once case is more stable than heterochromatin formed in the other, but "spreading" is identical in both. Distinguishing between these two models would be easier than some of the experiments in the manuscript, but one needs to acknowledge the competing model before it can be tested.

The concept of fidelity of spreading is never defined and is less than obvious, yet experiments are interpreted in the context of this fidelity entity.

The "memory" experiments are interesting but again memory implies a time over which something is remembered. The experiments in Figure 4 may bear upon memory, but they are done in a rather complicated way show the sensitivity of those cells that have been pregrown for 25 generations to a subsequent challenge of various concentrations of TSA with respect to the stability of their expression state at that one time. A demonstration of memory requires a time component over which the thing of interest is remembered, but this experiments design precludes that. To be sure, there is some interesting biology behind the curves in this figure, but at present the experimental design would allow the authors to make interesting claims about the contribution of the various elements to the efficiency of re-establishing heterochromatin…. interesting in itself, but it isn't memory. It seems to me that the issue of memory would be resolved by the kind of clever experiments performed in *S. cerevisiae* by the Broach and Gartenberg labs in which the nucleation sites is removed and then the persistence of a state is measured over time in the absence of the element that "nucleated" it. Alternatively, the microfluidic technology behind Figure 3 could be used to address this issue.

Reviewer #2:

The assembly of heterochromatin domains involves nucleation and spreading of silencing factors. However, the degree of fidelity and heritable stability among heterochromatin domains throughout the genome can vary. Here, the authors dissect the origins of the intrinsic high-fidelity and memory capacity associated with stable epigenetic heterochromatin states. To do this, they utilize a system that simultaneously monitors nucleation and spreading driven by individual regulatory elements. They find that heterochromatin spreading driven by an RNAi nucleation site (*cenH*) is multimodal and occurs stochastically. This finding is consistent when measured at ectopic sites as well as at the endogenous MAT locus. By contrast, they argue that spreading driven solely from the endogenous *REIII* element at the MAT locus shows no intermediate levels and is complete ("deterministic"). They conclude that the *REIII* element is required to preserve the epigenetic memory of an ancestral state, as well as to ensure heterochromatin domain maintenance during environmental stress.

A major strength of this article is the use of a system that quantitatively measures heterochromatic silencing in living cells. The development of such tools has allowed them to tackle key questions in the field of heterochromatin assembly. The conclusion that the spreading of heterochromatin from an RNAi nucleated element is stochastic is also supported by recent work by Obersriebnig et al., 2016, which also used single cell assays. A key new finding here is that a cis-acting element, *REIII*, promotes deterministic spreading of heterochromatin that has high memory capacity. However, in the absence of a mechanism for the *REIII* -mediated spreading, this aspect of the work remains preliminary. Moreover, the evidence suggesting deterministic spreading from *REIII* needs further careful evaluation.

1) Regarding the mechanism for the role of *REIII* in epigenetic memory, previous studies have provided important insights that are highly relevant to this story and deserve to be addressed clearly as part of the main discussion. *REIII* (a binding site for ATF-CREB proteins Atf1-Pcr1) was initially shown to be critical for the stability of heterochromatin at MAT, particularly when heterochromatin nucleation by RNAi is compromised (Jia et al., 2004). A recent study confirmed this function of *REIII* (Wang and Moazed, 2017). However, more relevant to this paper are subsequent studies that revealed the mechanism as follows:

Yamada et al., 2005 found that *REIII* recruits an HDAC Clr3 (see Figure 2), the activity of which is critical for the stability and spread of heterochromatin. Further work showed that Clr3 is a component of the SHREC complex that localizes to *REIII* (see Figure 4 in Cam et al., 2008). These studies set the stage for a critical finding that explained the role of *REIII* /Clr3 in heterochromatin stability. Aygun et al., 2013 discovered Clr3 HDAC as a unique factor that promotes heterochromatin stability by suppressing turnover of histones. Indeed, loss of Clr3 is linked to loss of tri-H3K9me (see Figure 5A in Yamada et al., 2005), which provides the binding site for the chromodomain of Clr4 to reinforce a feedback loop in which tri-H3K9me recruits Clr4 HMTase to promote heterochromatin spreading/maintenance (Zhang et al. 2008; also, Al-Sady et al., and Jih et al., 2017).

In short: *REIII* → Atf1-Pcr1 → Clr3/SHREC → Suppresses histone turnover/stabilizes tri-H3K9me → Clr4 chromo binding to tri-H3K9me → Heterochromatin spreading/maintenance via feedback loop.

In light of these previous findings, an important possibility is that mutations in *REIII* impair the recruitment of Clr3 HDAC (e.g. see Figure 3 in Yamada et al., 2005), which in turn results in increased in turnover of H3K9 methylated histones that are required for the spreading and maintenance of heterochromatin. The authors shall test whether ∆*REIII* cells show an elevated level of histone turnover, hence affecting spreading and epigenetic memory.

2) An important assumption in this study is that cells carrying ORANGEON or ORANGEoff states contain comparable levels of heterochromatin at the nucleation site. However, it is possible that ORANGEON cells contain a relatively lower level of heterochromatin (but still sufficient to repress GREEN with a weak promoter) than ORANGEoff cells (e.g. in WT and ∆*REIII* cells). This should be tested experimentally by assaying H3K9me levels by ChIP at GREEN and ORANGE loci. Also, GREEN inserted in *cenH* is likely silenced post transcriptionally (transcripts of markers inserted near centromeric repeats are converted to siRNAs) and its silencing might not provide an accurate measure of silencing caused by heterochromatin modifications. If true, this would complicate the interpretation of the results.

3) This reviewer is concerned that the evidence that *REIII* causes deterministic spreading is an artifact of the system. Unlike WT or ∆REII cells that contain GREEN at a bona fide nucleation site, from which heterochromatin is known to spread outward, GREEN in ∆K cells is likely not placed at a nucleation site (there is no evidence that *REIII* alone can nucleate heterochromatin, and no evidence is presented to show that heterochromatin spreads from *REIII* toward ORANGE). In cells with GREEN located closer to the edge of the heterochromatin domain, the silencing of ORANGE located in the middle of the silenced domain might precede GREEN. In this situation, when silencing of ORANGE is assayed in GREENoff cells it would give the false impression of deterministic spreading. Consistent with this, markers inserted near mat3 are known to be silenced less efficiently. Similarly, if nucleation in ∆K cells occurred on the left side of ORANGE, all cells showing GREENoff will carry the ORANGEoff state. Can they confirm that heterochromatin in ∆K cells is indeed nucleated at *REIII*, or if *REIII* simply stabilizes heterochromatin (for example by recruiting Clr3 HDAC Yamada et al., 2005 and Aygun et al., 2013)? Moreover, it is also possible that heterochromatin in ∆K cells is formed in such as a way that it does not involve stepwise mechanism of nucleation and spreading and that the entire domain is silenced simultaneously.

4) The authors argue in the abstract that the epigenetic capacity contributing to heterochromatin spreading and maintenance is not encoded in the spreading reaction itself, but rather requires specialized memory elements. This statement is misleading. The factors involved in spreading and maintenance of heterochromatin, such as HDACs, are known to be loaded across silenced domains by HP1, which is also required for heterochromatin spreading. However, these same factors (HDACs) can be also loaded by so called specialized/memory elements, further enhancing the stability of heterochromatin. In this respect, the epigenetic capacity is at least partially encoded in the spreading reaction and can be further strengthened by elements that also load activities, such as HDACs, promoting heterochromatin stability.

Reviewer #3:

The fidelity and robustness are two essential characteristics to epigenetic inheritance, which is important both for chromatin structure and cell differentiation in many species. Its full establishment comprises of two stages: nucleation and spreading, which can be executed through RNA interference (RNAi)-dependent and RNAi-independent pathways.

In this study, Greenstein and colleagues set up an elegant and sensitive single-cell reporter system to dissect these two characteristics. They have used it for population studies and cell-lineage tracking for trans-generation memory. Consistent with previous findings, they showed spreading of heterochromatin nucleated by RNAi alone is robust but low on fidelity, as compared to that nucleated by wild-type locus. Importantly, they have attributed the fidelity in heterochromatin spreading to an RNAi-independent DNA element *REIII*. Further, they have tested the persistence of "memory" as nucleated by RNAi or *REIII* with and without perturbations. The biological importance and complicated nature should make it appealing to a broad audience.

In Figure 2B and 2C, where modified nucleators of RNAi-alone or *REIII* -alone are tested, the fidelity in spreading and robustness in nucleation segregated clearly in these population studies. However, one concern still remains. As spreading is greatly affected by the distance from the nucleator to the reporter, the actual distance for *REIII* -nucleated spreading should be measured between *REIII* and "orange" reporter, rather than between "green" and "orange" reporter as they are on different sides of the *REIII* nucleator, and to be consistent with Figure 2A and 2B. If the genome feature in Figure 2C is drawn to scale, it is around 1-2 kb in distance. At this distance, even RNAi-nucleated heterochromatin is demonstrating high fidelity in spreading as in the top panel of Figure 1C. At least one testing should be done with the reporter cassette moved further away from *REIII* nucleator by inserting "inert" DNA sequence (from a euchromatic region), to the similar 3-4 kb distance scale as Figure 2A and 2B. It is necessary to exclude the possibility that higher fidelity as in Figure 2C is due to a shorter loop for spreading. If the experiment is not possible, it shall be clearly explained in the text.

[Editors’ note: what now follows is the decision letter after the authors submitted for further consideration.]

Thank you for resubmitting your work entitled "A memory element imposes epigenetic behavior on intrinsically labile ncRNA-induced heterochromatin spread" for further consideration at *eLife*. Your revised article has been reviewed by Kevin Struhl (Senior Editor), a Reviewing Editor, and two reviewers.

The manuscript has been improved but there are some remaining issues that need to be addressed before acceptance, as outlined below:

In the previous submission of this manuscript, the work presented was found to be potentially of great interest, particularly the discovery of a novel form of putative heterochromatin spreading, nucleated by the cis-acting element *REIII* that the authors claim has a deterministic role. However, direct evidence for *REIII* -mediated heterochromatin spreading was found to be lacking, and there was no mechanism. In their revised manuscript, the authors have provided several lines of new evidence to address some of the reviewers' comments. In particular, by deleting the *REIII* element, they show that this leads to increased turnover of histones which provides some insight into how *REIII* may be promoting epigenetic stability of heterochromatin. However, the conclusion that *REIII* promotes deterministic spreading of heterochromatin is still not really supported, as pointed out by reviewer 2. Furthermore, the argument that ∆K cells have better "memory" than WT cells is not supported by previous findings that ∆K cells contain metastable heterochromatin, with frequent conversion from the OFF to the ON state compared to WT. As both reviewers point out, it remains possible that bias may be introduced in the "memory" determinations, due to preselection for ∆K_OFF_ colonies.

Despite these limitations, and given the new work included in this manuscript on this *REIII* -based heterochromatin process, we would be willing to consider a revised text, that focused on the single cell assay showing that loss of *REIII* affects the stability of heterochromatin. In this revised manuscript, specific references to « memory elements » and « deterministic » spreading should be removed, as suggested by reviewer 2.

Reviewer #2:

The authors performed several experiments that addressed the reviewers' comments and strengthened their conclusions. For example, the extensive chromatin immunoprecipitation analyses performed to assay H3K9me2 and H3K9me3 levels in different strains now correlates changes in gene silencing with heterochromatin levels. Also, this reviewer suggested that they investigate the effects of *REIII* deletion on histone turnover, which has implications for heterochromatin spreading and epigenetic memory. They do indeed find that deletion of *REIII* causes increased turnover of histones. Together with previously published findings showing that *REIII* recruits HDAC Clr3 (Yamada et al., 2005), which is involved in the suppression of histone turnover (Aygun et al., 2013), this finding provides a possible mechanistic insight into how *REIII* promotes epigenetic stability of heterochromatin. With the new additions, this part of the story has improved significantly.

However, their conclusion that *REIII* promotes deterministic spreading of heterochromatin remains ambiguous. In particular, unlike *cenH* that has been shown to nucleate heterochromatin, there is no clear evidence presented that heterochromatin spreads outward from the *REIII* element in ∆K cells. Rather, given the bistable nature of heterochromatin at mat in ∆K cells, the assembly of heterochromatin in ∆K cells cells might involve cooperativity between yet unknown elements, as opposed to reliance on the *REIII* element alone. In presenting their case for deterministic spreading from *REIII*, the authors mention previous work showing that loss of *REIII* affects heterochromatin in ∆K cells. However, it is important to note that previous studies (such as Jia, Noma and Grewal, 2004 and Wang and Moazed,.2017) and this article only show that *REIII* is *required* for the stability of heterochromatin (most likely via recruitment of HDAC Clr3 that suppresses histone turnover to prevent loss of H3K9me) but is not *sufficient* to nucleate heterochromatin. Similarly, their argument that ∆K cells have better "memory" than WT cells contrasts with a previous finding that ∆K cells contain metastable heterochromatin, as indicated by the frequent conversion from the OFF to the ON state at a rate many fold higher than in WT. Moreover, it remains possible that bias may be introduced in the "memory" determinations, due to preselection for ∆K_OFF_ colonies (see subsection “Multi-generational single cell imaging reveals ncRNA-driven spreading to be unstable”), in which entire mat locus is known to be coated with heterochromatin (Nakayama, Klar and Grewal, 2000).

My recommendation is to prepare a revised manuscript for publication in *eLife* that focuses on the results from their elegant single cell assays showing that the loss of *REIII* affects the stability of heterochromatin and that this behavior of *REIII* is mechanistically linked to the suppression of histone turnover at mat. The discussion would be focused on succinctly describing this feature of *REIII* in the context of previous findings, including the results showing that *REIII* recruits the HDAC Clr3 (Yamada et al., 2005) that also suppresses histone turnover to promote epigenetic stability of heterochromatin (Aygun et al., 2013). Indeed, suppression of histone turnover by *REIII* /HDAC Clr3 may promote retention of methylated histones that are critical for the recruitment of Clr4; thus, promoting the efficient spreading of heterochromatin. Framing the results in this way would avoid the problematic issues raised by applying the terms "memory element" or "deterministic" spreading to *REIII*.

Reviewer #3:

The authors have addressed all the critiques raised by this reviewer. In particular, they have now provided more compelling evidence of "spreading" of heterochromatin from corresponding nucleation sites with a more complete testing cassette of different distances, directions, nucleation factors, etc. They have used these testing cassettes to demonstrate the stochasticity of ncRNA-mediated spreading and stability of *REIII* -mediated memory. In addition, they have now tested the anti-correlation of histone turnover and memory.

# Reply: Author response

**DOI:** 10.7554/eLife.32948.030

[Editors’ note: the author responses to the first round of peer review follow.]

Our decision has been reached after consultation between the reviewers. Based on these discussions and the individual reviews below, we regret to inform you that your work will not be considered further for publication in eLife. The overall conclusion is that this work is potentially of interest and could be worthy of publication in eLife in the future. However, in its current version it would require too much work and thus it cannot be deemed appropriate for revision within a reasonable time frame.

If you can incorporate the important critiques and suggestions made by the reviewers below, you are encouraged to resubmit your manuscript to eLife. In that case, the resubmitted manuscript would be considered a "new" submission and go through the same 2-stage process. However, unlike a true new submission, it would be typically seen by the editor and if needed the original reviewers. Summary: In this manuscript the authors set out to explore nature of heterochromatin spreading in fission yeast using a system that quantitatively measures heterochromatic silencing in living cells and follows the spreading reaction using fluorescent reporters. They report that spread of silencing from RNAi-nucleated elements is stochastic and multimodal and fluctuates over time. This is also supported by recent work by Obersriebnig et al. The authors also find another form of spreading, nucleated by the cis-acting element REIII, that they term deterministic, and that appears to show greater memory and fidelity in local spreading, being better buffered. This finding is novel. However, in the absence of a mechanism for REIII -mediated spreading, the work remains preliminary and there may be other interpretations of the data presented, as highlighted in the reviewers' comments. In summary, the authors do not really address the questions of heterochromatin spreading fidelity or memory as they set out to. In particular, evidence for actual "spreading" of heterochromatin from the so-called nucleation site and the genes assayed for silencing at some distance from it, is lacking. Based on the reviewers' comments, the overall conclusion is that this work is potentially of great interest and could be worthy of publication in eLife in the future. However, in its current version it would require too much work and thus it cannot be deemed appropriate for revision within a reasonable time frame.

We very much like to thank the editors and reviewers for their thoughtful feedback. We have made major additions to this work to address the reviewers’ comments. The experiments we have included here resulted in a more robust view of spreading in *S. pombe* and enable us to make mechanistic inferences about how the divergent spreading types we describeare enacted. Additionally, the data point to mechanisms underlying memory of repression that is required at a cell type specification locus like MAT. Together, they have made this a much stronger manuscript. The most important additions are as follows.

1) Establishing directionality of spreading: We have added an additional single cell tacking experiment in the ectopic strain, which together with our previous data, allows us to address the directionality of spreading using the FYLM device. We are happy to report that our data is consistent with a successive nucleation and spreading model. Additionally, we clarify our analysis method, which does not pre-filter cells of any nucleation proximal (“green”) or distal (“orange”) reporter values. We can now report that our cytometry without proximal or distal gating supports a nucleation seeded spreading model.

2) Tracking heterochromatin structure in our MAT locus strains: We have performed a suite of Chromatin Immuno-precipitation (ChIP) experiments to address the following issues: a. correlation of repression and heterochromatin structure, b. the stability of nucleation elements in strong and weak spreading strains and c. *REIII -*dependent heterochromatin formation. We can report that the new data support the conclusion that differences in spreading manifest as weaker heterochromatin but are not rooted in weak nucleation.

*3) REIII* mechanism and memory: We added an additional experiment, where we remove nucleation factors post-establishment, which more directly addresses the differences in memory over time in our strains. We provide a mechanistic explanation for increased or decreased memory by tracking histone 3 turnover at euchromatic and MAT locus heterochromatin sites. We can report that *REIII* confers a high degree of memory that is linked to very low histone turnover compared with strains nucleated by RNA-processes that show more euchromatic levels of histone turnover.

We have additionally adopted the reviewers’ suggestions to simplify and clarify the writing. Finally, both reviewer #1 and #2, compared our findings to those of Obersriebnig et al., a previous study of heterochromatin in single fission yeast cells. This was an important study and we would like to clarify the differences in experimental design and conclusions that distinguishes the two reports.

In the Obersriebnig work, heterochromatin formation was tracked either by following one color, at *cenH,* near *REII* or *REIII,* or with two colors, one at *cenH* and one near *REIII* in wild-type MAT. Since in all cases both *REIII* and *cenH* nucleators were intact, silencing of either color cannot be ascribed to spreading. In our work, we both inactivate one of the nucleators at a time and track nucleation behavior with one color and spreading with a second. The use of one color can yield silencing kinetics, but by design, the nucleation and spreading events cannot be untangled. The authors make claims about the stochasticity of heterochromatin establishment, which is novel, but not spreading, as is consistent with their experimental design. Conclusions they draw about establishment kinetics are noteworthy, yet those about spreading kinetics again are complicated by having both nucleators intact. This is important, because we believe the novelty of our work, beyond the novel behavior of *REIII,* is the ability to isolate any type of spreading event from the DNA/RNA sequence-driven nucleation component and define its characteristics, including in real time.

Reviewer #1:

The fundamental problem conceptual with this paper is that the experiments are designed and interpreted in the context of a model in which spreading is assumed to be a process that emanates from a regulatory site and progressively reaches a detector site or sites. There is no evidence that "spreading" of heterochromatin of this type actually occurs in S. pombe or its relative S. cerevisiae. The crucial missing datum for all spreading models is some kind of time resolution between events at the cis-regulatory site, called the nucleation site in this study and others, and the genes at a distance that fall under the control of this regulatory site.

We thank the reviewer for the comments which helped improve this work.

Spreading outward from nucleation sites, linearly or via loops, is the paradigm for propagating heterochromatin along the chromosome in *S. pombe* (Hall et al., 2002; Li et al., 2009; Noma et al., 2004; Zhang et al., 2008), *S. cerevisiae* (reviewed in (Kueng et al., 2013)), flies (reviewed in (Elgin and Reuter, 2013)), plants (Yang et al., 2017) and humans (Tchasovnikarova et al., 2015). However, we agree that experiments documenting kinetic separation of nucleation and spreading are rare. One example is vernalization of the *FLC* locus in *Arabidopsis*, where nucleation and spreading are separated in time (Yang et al., 2017). Our single cell tracking data already contained information that addresses this question directly, which we expanded in the revision:

Three lines of evidence establish that heterochromatin spreads outward from nucleation sites in our assays. First, we examined the kinetics of proximal and distal silencing in cells where the system switches from full derepression to repression. Our 3kb ectopic strain afforded us an opportunity to do so, (~5% of the cells in FYLM single cell tracking data of our 3kb ectopic strain, new Figure 3—figure supplement 1), while the MAT locus strains remain near uniformly repressed in the “green” nucleation proximal color. Full silencing of the “green” nucleation-proximal color almost exclusively precedes the distal “orange” spreading color in time (N=15/270 cells with repression events where “green” and “orange” start out fully de-repressed, see new Figure 3—figure supplement 1A,B). We find only one example (cat 4, 1/270) with “orange” repression preceding “green”, with the green closely following orange. This small kinetic gap therefore may suggest a fast spreading event.

Second, in the FYLM data, we did not observe any cells (N=0/270) where “green” becomes de-repressed while “orange” stays repressed (see new Figure 3—figure supplement 1A). In contrast we find many cells in which “orange” fluctuates while “green” is OFF for the entire measured lifespan (N=36/270, new Figure 3—figure supplement 1A,C) and more cells where “orange” is fully de-repressed while “green” is at least repressed partially for the entire measured lifespan (N=61/270, new Figure 3—figure supplement 1A,D). This reinforces the notion that silencing is directional with “green” preceding “orange”.

Third, using our steady state flow cytometry data for all RNA- nucleated strains, both ectopic and at MAT, gating for “green”^OFF^ (nucleation site) yields a distribution of orange repression, OFF to ON, while gating in “orange”^OFF^ (spreading site) only yields “green”^OFF^, supporting the above single-cell dynamics with a broader, population-based snapshot (see 2D-hexbins below as well as Figure 1C insets, Figure 2B,C,D). Therefore, we interpret this to indicate that spreading follows nucleation.

Moreover, the way the experiments are conducted, for example by starting with cells and colonies that are preselected to be nucleated (green off) prevents the authors from the opportunity to observe the very data that could falsify the hypothesis (i.e. observing cells in which green is on and orange is off.)

We apologize if our analysis method was not sufficiently clear, and we have now clarified the text. In fact, we do not preselect “green”^OFF^ cells for analysis: All the 2D histogram and scatter plots in the paper show cells normalized only by “red”. No “green” or “orange” value filter is applied at this stage. Even for the Δ*K* strain, which can be isolated as ON or OFF alleles (Grewal and Klar, 1996), we describe the overall distribution of cells after introduction of *clr4+* in Figure 2E. Since we established from our data (cytometry and single cell tracking) that nucleation indeed precedes spreading, we produce 1D “orange” histograms with an additional “green”^OFF^ gate to isolate fully nucleated cells (Figure 1C, insets Figure 2B, C, E). Importantly, in the cytometry data, we do not observe cell populations that are “orange”^OFF^ and “green”^ON^ (Figure 1C inset plots are not pre-filtered and almost contain no cells in bottom right quadrant, highlighted for ectopic strains in Author response image 1). We believe our data is consistent with directional spreading from nucleation to distal sites and hope this clarification highlights that we include all cells with respect to “green” and “orange” expression for analysis.

**Author response image 1.** Exceedingly few cells are “green”ON and “orange”OFF. 2D hexbin plots of cells (max and min bin cell numbers indicated) with ectopic nucleation site (“green” nucleation proximal, “orange” distal at indicated distances, see Figure 1C). Fluorescence of all cells gated for cell size is plotted and normalized by “red” with no green or orange filtering. Note the very small number of cells detectable in the bottom right quadrant.

The second major problem in the paper is the assertion of being able to distinguish between stochastic spreading and deterministic spreading. Let's for the moment imagine that spreading actually occurs. The distinction the authors make between stochastic and deterministic spreading is based upon the range of expression of the detector genes that are positioned to be under the influence of the nucleation site. If that range of expression is narrow, spreading is defined to be deterministic. If it is broad, spreading is defined to be stochastic. The competing model, never mentioned nor tested, is that heterochromatin formed in once case is more stable than heterochromatin formed in the other, but "spreading" is identical in both. Distinguishing between these two models would be easier than some of the experiments in the manuscript, but one needs to acknowledge the competing model before it can be tested.

The reviewer points out an alternative possible scenario for differences in spreading, which we now address in the text. It could be envisaged that the spreading probability depends primarily on the how stability of heterochromatin the nucleation site. In such a scenario the frequency of spreading would correlate precisely with the stability of the nucleation site. This model predicts that a narrow range of repression should therefore correlate with very robust heterochromatin, and conversely that spreading that has a wider range of states when the heterochromatic state is less stable or robust. Two lines of evidence argue against this possibility:

We examined the stability of the relevant nucleation sites to test this hypothesis, *cenH* in the wild-type (“orange” mostly repressed) or *REIII -*disrupted context (Δ*REIII*, stochastic spreading) and *REIII* in the *cenH* deleted context (Δ*K*, deterministic spreading). To do so, we performed Chromatin Immuno-precipitation.

(ChIP) experiments for the heterochromatin assembly mark H3K9me2 and the repressive/spreading mark H3K9me3 (see (Al-Sady et al., 2013; Jih et al., 2017)). Since factors assembling the heterochromatin structure are directly or indirectly attracted by these marks, we believe that they are the central features to examine. We observe that at all nucleation sites, the degree of both H3K9me2 and H3K9me3 methylation is extremely robust (around the value of the pericentromeric *dh*) and nearly invariant between the nucleation sites. Critically, there is no difference in H3K9me2 and me3 methylation at *cenH* between wildtype MAT and Δ*REIII,* strainswhich feature very different distributions of distal “orange” repression (Figure 2B, C). This new data is included in Figure 2D and E.

**Author response image 2.** “green” at cenH shows the same resistance to TSA irrespective of spreading behavior. The same cells plotted in Figure 4B and C are plotted for the 0 TSA condition. Note that green lines represent fraction of total “green”OFF, orange lines represent fraction of “orange” repressed cells within “green”OFF.

The TSA experiment in Figure 4 additionally afforded us an opportunity to compare directly the stability of the same *cenH* heterochromatin region in near-isogenic strains that spread robustly (wild-type MAT^HSS^) or stochastically (∆*REIII*^HSS^). To this end we plotted the fraction of “green”^OFF^ cells along the [TSA] gradient (Author response image 2, relative to the 0 TSA point, =1) to determine if heterochromatin is differentially stable at the *cenH* locus between these strains. We found that the curves for “green” are essentially super-imposable, while the “orange” differs markedly, as shown in Figure 4B and C.

Together, we believe that both these new ChIP data and reanalysis of our existing TSA data shown here are not consistent with a model in which the differences in “orange” repression reflect stability of the heterochromatin domain.

Rather we believe that they more consistent with distinct heterochromatin spreading behaviors in these strains.

The concept of fidelity of spreading is never defined and is less than obvious, yet experiments are interpreted in the context of this fidelity entity.

We thank the reviewer for the helpful comments concerning terminology. We define spreading fidelity as the accurate inheritance of the positional and repressive extent of heterochromatin formed by spreading. High fidelity spreading should manifest as a tight distribution of states in the population and invariant inheritance when single cells are tracked. We have clarified and simplified terminology throughout the manuscript.

The "memory" experiments are interesting but again memory implies a time over which something is remembered. The experiments in Figure 4 may bear upon memory, but they are done in a rather complicated way show the sensitivity of those cells that have been pregrown for 25 generations to a subsequent challenge of various concentrations of TSA with respect to the stability of their expression state at that one time. A demonstration of memory requires a time component over which the thing of interest is remembered, but this experiments design precludes that. To be sure, there is some interesting biology behind the curves in this Figure, but at present the experimental design would allow the authors to make interesting claims about the contribution of the various elements to the efficiency of re-establishing heterochromatin…. interesting in itself, but it isn't memory. It seems to me that the issue of memory would be resolved by the kind of clever experiments performed in S. cerevisiae by the Broach and Gartenberg labs in which the nucleation sites is removed and then the persistence of a state is measured over time in the absence of the element that "nucleated" it. Alternatively, the microfluidic technology behind Figure 3 could be used to address this issue.

Measuring events over time is a central way to assess memory in a system, and we have provided additional measurements in this vein in the revised version. However, it is only one of several approaches to probe memory. The TSA experiments borrow conceptually from hysteresis measurements, which are a rigorous means to probe the memory capacity in the system, illustrated by Ferrell and colleagues (Bagowski and Ferrell, 2001). In this analysis, memory is manifested by the system behaving differently depending on its starting state. A stimulant-responsive system exhibits hysteresis (memory) if the behavior along the stimulant gradient is divergent between a fully stimulated or naïve starting condition. The similarity in molecular architectures, cooperativity and positive feedback, which drive both the biochemical systems traditionally subjected to hysteresis analysis and epigenetic systems (see (Angel et al., 2015; Dodd et al., 2007)), motivated us to perform hysteresis-type experiments. Here, we are probing whether the ancestral state biases the decision of system to undergo a spreading event. We measure the retention of this bias along a gradient of inhibitor, which is conceptually similar to measurements for a stimulant. We chose this course because stimulants are not readily available for heterochromatin. We believe this analysis is both rigorous and valid for probing memory capacity.

However, inspired by the reviewer’s invocation of the Gartenberg experiments, we probed the effect of removing *trans-*acting nucleation factors on the retention of the state originally formed by spreading. These experiments are conceptually similar to removal of *cis-*acting sites but avoid the caveat of low induced Cre-lox recombination frequency in *S. pombe.* We crossed our reporter strains to mutants required for either the *cenH* or *REIII* nucleator. Immediately following detection of cross progeny colonies, we proceeded with flow cytometry analysis. We find indeed that *REIII* nucleated heterochromatin can maintain itself after removal of a required *trans-*acting nucleation factor for ~ 200 generations, while *cenH*-nucleated heterochromatin cannot, and appears to require continual nucleation to maintain the state. These new data support our overall conclusions and are presented in the new Figure 5 and new Figure 5—figure supplement 1.

Finally, we also believe that our single cell tracking data in Figure 3 and Figure 3—figure supplement 2 lend independent support for the conclusion that *REIII* spreading is remembered, while *cenH-*spreading is not. Intergenerational tracking of spreading in the *cenH,* noncoding RNA (ncRNA)*-nucleatedstrains (Figure 3D) as well as ectopically ncRNA-nucleated strains (Figure 3—figure supplement 1 and Figure 3—figure supplement 2) show fluctuations of “orange” not compatible with strong memory, while *REIII* strains (Figure 3E) show no such fluctuations over similar generation times, strongly implying memory.

Together, our three independent approaches strongly support a differential in the ability to remember the state formed by spreading between *cenH* and *REIII* nucleated spreading.

We now use this more general descriptor for *cenH* and *dh* rather than RNAi.

Reviewer #2: The assembly of heterochromatin domains involves nucleation and spreading of silencing factors. However, the degree of fidelity and heritable stability among heterochromatin domains throughout the genome can vary. Here, the authors dissect the origins of the intrinsic high-fidelity and memory capacity associated with stable epigenetic heterochromatin states. To do this, they utilize a system that simultaneously monitors nucleation and spreading driven by individual regulatory elements. They find that heterochromatin spreading driven by an RNAi nucleation site (cenH) is multimodal and occurs stochastically. This finding is consistent when measured at ectopic sites as well as at the endogenous MAT locus. By contrast, they argue that spreading driven solely from the endogenous REIII element at the MAT locus shows no intermediate levels and is complete ("deterministic"). They conclude that the REIII element is required to preserve the epigenetic memory of an ancestral state, as well as to ensure heterochromatin domain maintenance during environmental stress. A major strength of this article is the use of a system that quantitatively measures heterochromatic silencing in living cells. The development of such tools has allowed them to tackle key questions in the field of heterochromatin assembly. The conclusion that the spreading of heterochromatin from an RNAi nucleated element is stochastic is also supported by recent work by Obersriebnig et al,. 2016, which also used single cell assays. A key new finding here is that a cis-acting element, REIII, promotes deterministic spreading of heterochromatin that has high memory capacity. However, in the absence of a mechanism for the REIII -mediated spreading, this aspect of the work remains preliminary. Moreover, the evidence suggesting deterministic spreading from REIII needs further careful evaluation.

We thank the reviewer for this assessment.

1) Regarding the mechanism for the role of REIII in epigenetic memory, previous studies have provided important insights that are highly relevant to this story and deserve to be addressed clearly as part of the main discussion. REIII (a binding site for ATF-CREB proteins Atf1-Pcr1) was initially shown to be critical for the stability of heterochromatin at MAT, particularly when heterochromatin nucleation by RNAi is compromised (Jia et al., 2004). A recent study confirmed this function of REIII (Wang and Moazed, 2017). However, more relevant to this paper are subsequent studies that revealed the mechanism as follows: Yamada et al., 2005 found that REIII recruits an HDAC Clr3 (see Figure 2), the activity of which is critical for the stability and spread of heterochromatin. Further work showed that Clr3 is a component of the SHREC complex that localizes to REIII (see Figure 4 in Cam et al., 2008). These studies set the stage for a critical finding that explained the role of REIII /Clr3 in heterochromatin stability. Aygun et al., 2013 discovered Clr3 HDAC as a unique factor that promotes heterochromatin stability by suppressing turnover of histones. Indeed, loss of Clr3 is linked to loss of tri-H3K9me (see Figure 5A in Yamada et al., 2005), which provides the binding site for the chromodomain of Clr4 to reinforce a feedback loop in which tri-H3K9me recruits Clr4 HMTase to promote heterochromatin spreading/maintenance (Zhang et al. 2008; also, Al-Sady et al., and Jih et al., 2017). In short: REIII → Atf1-Pcr1 → Clr3/SHREC → Suppresses histone turnover/stabilizes tri-H3K9me → Clr4 chromo binding to tri-H3K9me → Heterochromatin spreading/maintenance via feedback loop In light of these previous findings, an important possibility is that mutations in REIII impair the recruitment of Clr3 HDAC (e.g. see Figure 3 in Yamada et al..,2005), which in turn results in increased in turnover of H3K9 methylated histones that are required for the spreading and maintenance of heterochromatin. The authors shall test whether ∆REIII cells show an elevated level of histone turnover, hence affecting spreading and epigenetic memory.

We thank the reviewer for raising these points. We had included a description of possible mechanisms in our supplemental discussion in the original submission, including a number of the references cited by the reviewer. However, we recognize that the mechanistic arguments should be highlighted more prominently in the paper and have included them now in the main discussion.

We have now addressed the role of histone turnover as a possible mechanism for the differences in spreading and epigenetic memory by performing additional experiments. We adopted the published (Audergon et al., 2015; Svensson et al., 2015) Recombination Induced Tag Exchange system (RITE) to probe H3 turnover. Given the labile nature of heterochromatin at elevated temperatures (Figure 4E,F and Figure 4—figure supplement 2), replication stalling was performed with hydroxyurea as published (Aygun et al., 2013). We address turnover in the ∆*K*^HSS-OFF^, ∆*K*^HSS-ON^ and ∆*REIII*^HSS^ strains to address the role of *REIII,* with ∆*K*^HSS-ON^ serving as an isogenic non-heterochromatic control for ∆*K*^HSS-OFF^. We measured H3 turnover at two euchromatic control loci and find no difference between the strains. However, when we examined turnover at MAT using amplicons that are shared by all strains, we found significantly decreased histone turnover in ∆*K*^HSS-OFF^, while ∆*K*^HSS-ON^ and ∆*REIII*^HSS^ consistently display elevated turnover, more consistent with euchromatic behavior. These new data are included in the new Figure 6 A,B. The data that histone turnover is elevated in the absence of nucleated *REIII* thus presents a mechanistic explanation for the strongly reduced memory we see in _∆_*REIII* and stochastic spreading, while the exceedingly low turnover in the presence of *REIII* accounts for the deterministic spreading and robust memory. The robust memory is underlined by a new experiment we include, where we remove by cross factors required for nucleation following establishment (new Figure 5).

2) An important assumption in this study is that cells carrying ORANGEON or ORANGEoff states contain comparable levels of heterochromatin at the nucleation site. However, it is possible that ORANGEON cells contain a relatively lower level of heterochromatin (but still sufficient to repress GREEN with a weak promoter) than ORANGEoff cells (e.g. in WT and ∆REIII cells). This should be tested experimentally by assaying H3K9me levels by ChIP at GREEN and ORANGE loci. Also, GREEN inserted in cenH is likely silenced post transcriptionally (transcripts of markers inserted near centromeric repeats are converted to siRNAs) and its silencing might not provide an accurate measure of silencing caused by heterochromatin modifications. If true, this would complicate the interpretation of the results.

We thank the reviewer for the comment. We have now performed extensive Chromatin Immuno-precipitation (ChIP) analysis for the heterochromatin assembly mark H3K9me2 and repressive/spreading mark H3K9me3 across the MAT locus.

Specifically, we find:

1) H3K9me2 and H3K9me3 marks accumulate to similar extents at the *cenH* nucleation site (“green” in WT and ∆*REIII*^HSS^), even though this strain, unlike WT, features a high proportion of “orange”^ON^ (new Figure 2D). We also find very similar proportion of H3K9me2 and me3 at “green” in ∆*K*^HSS-OFF^, see the new data in Figure 2E. H3K9me2 and me3 marks accumulate to very high extents at these sites in the three strains, which is not consistent with heterochromatin spreading behaviors being solely the result of different level of heterochromatin assembly at nucleation regions.

2) H3K9me2 and me3 marks decline rightwards of *cenH* only in the ∆*REIII*^HSS^, but not wild type stain. This is evident even at the edge of *cenH,* and more strongly at the more distal “orange” (Figure 2D). However, H3K9me2 and me3 do not significantly decline at “orange” in ∆*K*^HSS-OFF^ versus “green” (Figure 2E). We note that for H3K9me3 only there is a small, but not significant, difference between “green” and “orange”.

Together, these data are consistent with differences in heterochromatin spreading, as opposed to weakened nucleation, driving the differences in distal “orange” repression, and further that our single cell silencing data for “orange” accurately reflects differences in the spreading of heterochromatin outwards from nucleation sites.

In *S. pombe*, heterochromatin assembled via noncoding RNA (ncRNA)* processes triggers co-transcriptional silencing as the reviewer states. However, transcriptional silencing also plays important roles (Fischer et al., 2009) at these sites, and silencing broadly correlates well with H3K9me2 accumulation, a read-out of heterochromatin structure. Yet, it remains possible that placement within an RNAi generating element affects the behaviors we track. We believe the reviewer’s concern ought to be mitigated by a difference in the placement of “green” at ectopic loci versus the MAT locus. In ectopic strains, “green” (for *ura4*, “orange” for *his1*) is placed immediately adjacent but not within the *dh* element, reporting on silencing at the nucleation locus, while for MAT strains “green” is within *cenH.* As we find the behavior with respect to spreading to be similar (e.g. Figure 1C 3kb versus Figure 2C) we conclude that the nature of H3K9me- dependent silencing at the nucleation marker not to interfere with our interpretation.

*We now use this more general descriptor for *cenH* and *dh* rather than RNAi

3) This reviewer is concerned that the evidence that REIII causes deterministic spreading is an artifact of the system. Unlike WT or ∆REII cells that contain GREEN at a bona fide nucleation site, from which heterochromatin is known to spread outward, GREEN in ∆K cells is likely not placed at a nucleation site (there is no evidence that REIII alone can nucleate heterochromatin, and no evidence is presented to show that heterochromatin spreads from REIII toward ORANGE). In cells with GREEN located closer to the edge of the heterochromatin domain, the silencing of ORANGE located in the middle of the silenced domain might precede GREEN. In this situation, when silencing of ORANGE is assayed in GREENoff cells it would give the false impression of deterministic spreading. Consistent with this, markers inserted near mat3 are known to be silenced less efficiently. Similarly, if nucleation in ∆K cells occurred on the left side of ORANGE, all cells showing GREENoff will carry the ORANGEoff state. Can they confirm that heterochromatin in ∆K cells is indeed nucleated at REIII, or if REIII simply stabilizes heterochromatin (for example by recruiting Clr3 HDAC Yamada et al., 2005 and Aygun et al., 2013)? Moreover, it is also possible that heterochromatin in ∆K cells is formed in such as a way that it does not involve stepwise mechanism of nucleation and spreading and that the entire domain is silenced simultaneously.

We thank the reviewer for this comment.

Recent evidence suggests that in the absence of *cenH,* Atf1/Pcr1 binding sites at *REIII* are absolutely required for heterochromatin formation (Figure 3C in (Wang and Moazed, 2017)). Independently of these studies, three lines of evidence from our work suggest that *ΔK*^HSS^ reports specifically on *REIII -*nucleated spreading, detected by “green”, towards “orange”:

1) Deletion of both Atf1/Pcr1 binding sites at *REIII* blocks any silencing in *ΔK*^HSS^ (in 34/34 strains examined) and H3K9 methylation at “green” or “orange”. This new data is presented in Figure 2—figure supplement 2A,B. Because *ΔREIII* does not abolish heterochromatin at MAT, i.e. it is not required to stabilize heterochromatin formed elsewhere (Figure 2C), the above result points to *REIII* nucleating heterochromatin in *ΔK*^HSS^.

2) No repressive elements other than *REII, cenH* and *REIII* have been identified at MAT. Technically, it possible that in *ΔK*^HSS^, which lacks *cenH*, silencing is due to *REII*. We had addressed this in the original submission by probing *ΔK*^HSS^ *REII::LEU2*, in which the *REII* element is deleted. This strain behaved similarly to *ΔK*^HSS^ (Figure 2—figure supplement 1C), strongly suggesting that in *ΔK*^HSS^, *REIII* is the only functional nucleator.

3) We should note that in our 2D histogram and scatter plots (Figure 1, Figure 2 and Figure 5) cells are not gated for “green”^OFF^. Rather, all cells within the size gate are plotted normalized by “red”. In those 2D plots, no significant de-repression of “green” is evident in *ΔK*^HSS^ in the repressed population. We only use the “green”^OFF^ gate for 1D histograms, to plot spreading in cells that fully nucleate. To address the point concerning spreading from leftwards of the “orange” nucleator, we moved “green” 2.1 kb towards the right *IR-R* boundary in *ΔK*^HSS^. This did not lead to the appearance of significant de-repression of “green” in “orange”^OFF^ cells, which might be expected if spreading emanated from a site leftwards of “orange” in *ΔK*^HSS^. This new data is presented Figure 2—figure supplement 2 C,D.

Together, we believe these lines of evidence and previously published data affirms that *REIII* is indeed the nucleating element in *ΔK*^HSS^ and that it spreads heterochromatin outward.

As to the comment about a non-stepwise mechanism, this is entirely possible for *ΔK*^HSS^ and we had discussed this possibility in the previous supplemental, and now moved it to the main discussion. Heterochromatin spreading can occur by a number of theoretical mechanisms (Talbert and Henikoff, 2006), such as linear “oozing”, skipping or looping. Looping mechanisms have been invoked for the polycomb system (Bantignies and Cavalli, 2011). Looping is one possible mechanism to account for deterministic behavior of *ΔK*, while linear “oozing” is more consistent with our observations for ncRNA-nucleated spreading (Figure 1, Figure 2C). In experiments outside the scope of this work, we look forward to testing this hypothesis.

4) The authors argue in the Abstract that the epigenetic capacity contributing to heterochromatin spreading and maintenance is not encoded in the spreading reaction itself, but rather requires specialized memory elements. This statement is misleading. The factors involved in spreading and maintenance of heterochromatin, such as HDACs, are known to be loaded across silenced domains by HP1, which is also required for heterochromatin spreading. However, these same factors (HDACs) can be also loaded by so called specialized/memory elements, further enhancing the stability of heterochromatin. In this respect, the epigenetic capacity is at least partially encoded in the spreading reaction and can be further strengthened by elements that also load activities, such as HDACs, promoting heterochromatin stability.

We thank the reviewer for raising this point. We have modified the language in the Abstract to reflect that the ncRNA-nucleated spreading reaction specifically does not confer the epigenetic fidelity required for robust and precise epigenetic inheritance, which additionally requires memory elements.

Reviewer #3: The fidelity and robustness are two essential characteristics to epigenetic inheritance, which is important both for chromatin structure and cell differentiation in many species. Its full establishment comprises of two stages: nucleation and spreading, which can be executed through RNA interference (RNAi)-dependent and RNAi-independent pathways. In this study, Greenstein and colleagues set up an elegant and sensitive single-cell reporter system to dissect these two characteristics. They have used it for population studies and cell-lineage tracking for trans-generation memory. Consistent with previous findings, they showed spreading of heterochromatin nucleated by RNAi alone is robust but low on fidelity, as compared to that nucleated by wild-type locus. Importantly, they have attributed the fidelity in heterochromatin spreading to an RNAi-independent DNA element REIII. Further, they have tested the persistence of "memory" as nucleated by RNAi or REIII with and without perturbations. The biological importance and complicated nature should make it appealing to a broad audience.

We thank the reviewer for the favorable assessment of this work.

In Figure 2B and 2C, where modified nucleators of RNAi-alone or REIII -alone are tested, the fidelity in spreading and robustness in nucleation segregated clearly in these population studies. However, one concern still remains. As spreading is greatly affected by the distance from the nucleator to the reporter, the actual distance for REIII -nucleated spreading should be measured between REIII and "orange" reporter, rather than between "green" and "orange" reporter as they are on different sides of the REIII nucleator, and to be consistent with Figure 2A and 2B. If the genome feature in Figure 2C is drawn to scale, it is around 1-2 kb in distance. At this distance, even RNAi-nucleated heterochromatin is demonstrating high fidelity in spreading as in the top panel of Figure 1C. At least one testing should be done with the reporter cassette moved further away from REIII nucleator by inserting "inert" DNA sequence (from a euchromatic region), to the similar 3-4 kb distance scale as Figure 2A and 2B. It is necessary to exclude the possibility that higher fidelity as in Figure 2C is due to a shorter loop for spreading. If the experiment is not possible, it shall be clearly explained in the text.

While *REIII* contains two potential nucleation sites (s1 and s2), only the distal (s2) sites has been implicated as the dominant nucleation element (Thon et al., 1999). The distance in *ΔK^HSS^*from the end of the spreading sensor ORF to s1 is ~1kb, and to s2 ~2.2kb. In comparison, the comparable distance in *ΔREIII HSS* is ~3.8kb. To mitigate the concern that the spreading distance in *ΔK* may be much shorter than in *ΔREIII* (i.e., from the ORF end to s1), we deleted s1 in the *ΔK* strain (*ΔKΔs1*) and inserted two different ~700bp sections of the *sib1* gene ORF between the spreading sensor and the s2 site in the *ΔKΔs1* background, increasing the spreading distance to ~3kb. Importantly, in both of these strains, we see the same deterministic behavior as in the *ΔK* parent. One of the *ΔKΔs1* +700bp insertions, representative of two behaviorally indistinguishable insertion strains we constructed, is shown in the new Figure 2—figure supplement 2E. Larger insertions where not compatible with heterochromatin nucleation in *ΔK*. Given these data, and the observation that significant stochastic spreading and intermediate states are detected for the 3kb, and even 1kb spreading distance with ectopically placed ncRNA nucleators (Figure 1), we conclude that the deterministic behavior is *ΔK* in intrinsic to nucleation from the *REIII* element.

[Editors’ note: the author responses to the re-review follow.]

The manuscript has been improved but there are some remaining issues that need to be addressed before acceptance, as outlined below: In the previous submission of this manuscript, the work presented was found to be potentially of great interest, particularly the discovery of a novel form of putative heterochromatin spreading, nucleated by the cis-acting element REIII that the authors claim has a deterministic role. However, direct evidence for REIII -mediated heterochromatin spreading was found to be lacking, and there was no mechanism. In their revised manuscript, the authors have provided several lines of new evidence to address some of the reviewers' comments. In particular, by deleting the REIII element, they show that this leads to increased turnover of histones which provides some insight into how REIII may be promoting epigenetic stability of heterochromatin. However, the conclusion that REIII promotes deterministic spreading of heterochromatin is still not really supported, as pointed out by reviewer 2. Furthermore, the argument that ∆K cells have better "memory" than WT cells is not supported by previous findings that ∆K cells contain metastable heterochromatin, with frequent conversion from the OFF to the ON state compared to WT. As both reviewers point out, it remains possible that bias may be introduced in the "memory" determinations, due to preselection for ∆KOFF colonies. Despite these limitations, and given the new work included in this manuscript on this REIII -based heterochromatin process, we would be willing to consider a revised text, that focused on the single cell assay showing that loss of REIII affects the stability of heterochromatin. In this revised manuscript, specific references to « memory elements » and « deterministic » spreading should be removed, as suggested by reviewer 2. Reviewer #2: The authors performed several experiments that addressed the reviewers' comments and strengthened their conclusions. For example, the extensive chromatin immunoprecipitation analyses performed to assay H3K9me2 and H3K9me3 levels in different strains now correlates changes in gene silencing with heterochromatin levels. Also, this reviewer suggested that they investigate the effects of REIII deletion on histone turnover, which has implications for heterochromatin spreading and epigenetic memory. They do indeed find that deletion of REIII causes increased turnover of histones. Together with previously published findings showing that REIII recruits HDAC Clr3 (Yamada et al., 2005), which is involved in the suppression of histone turnover (Aygun et al., 2013), this finding provides a possible mechanistic insight into how REIII promotes epigenetic stability of heterochromatin. With the new additions, this part of the story has improved significantly.

We thank the reviewer for this positive assessment.

However, their conclusion that REIII promotes deterministic spreading of heterochromatin remains ambiguous. In particular, unlike cenH that has been shown to nucleate heterochromatin, there is no clear evidence presented that heterochromatin spreads outward from the REIII element in ∆K cells. Rather, given the bistable nature of heterochromatin at mat in ∆K cells, the assembly of heterochromatin in ∆K cells cells might involve cooperativity between yet unknown elements, as opposed to reliance on the REIII element alone. In presenting their case for deterministic spreading from REIII, the authors mention previous work showing that loss of REIII affects heterochromatin in ∆K cells. However, it is important to note that previous studies (such as Jia, Noma and Grewal, 2004 and Wang and Moazed,.2017) and this article only show that REIII is required for the stability of heterochromatin (most likely via recruitment of HDAC Clr3 that suppresses histone turnover to prevent loss of H3K9me) but is not sufficient to nucleate heterochromatin.

The reviewer raises an important point. In ∆K cells, formation of H3K9me- marked heterochromatin is dependent on the action of *REIII* Atf1 and Pcr1 binding sites. Since there is no pre-existing H3K9me before introduction of *clr4+* in our experiments, heterochromatin nucleation must occur in some fashion in ∆K cells. However, sufficiency of Atf1 and Pcr1 binding sites for nucleation remains to be demonstrated. Specifically, *REIII,* unlike *cenH (*e.g. Hall el al., 2002) cannot effect significant repression when placed ectopically in the genome (references cited and Figure 2—figure supplement 2). This result can be interpreted in at least two ways: *REIII* is not by itself sufficient to nucleate and requires other, yet to be defined elements, or, the local genomic context/or chromatin organization of *REIII* within MAT is required for its nucleation activity, hence it cannot function ectopically. Since it cannot be easily resolved which case is correct, we now soften the conclusion that *REIII* drives spreading in ∆K cells in the text and remove the term “deterministic spreading” in favor of *REIII -*dependent heterochromatin.

Similarly, their argument that ∆K cells have better "memory" than WT cells contrasts with a previous finding that ∆K cells contain metastable heterochromatin, as indicated by the frequent conversion from the OFF to the ON state at a rate many fold higher than in WT. Moreover, it remains possible that bias may be introduced in the "memory" determinations, due to preselection for ∆K_OFF_ colonies (see subsection “Multi-generational single cell imaging reveals ncRNA-driven spreading to be unstable”), in which entire mat locus is known to be coated with heterochromatin (Nakayama, Klar and Grewal, 2000).

We thank the reviewer for this point. Here, we (1) Clarify our observations in the context of the literature, (2) Revise our interpretation of Figure 4D, and (3) Clarify our ∆K selection process.

Broadly, our results from ∆K cells are consistent with the published literature, namely a very low transition rate form OFF to ON per generation, about 10^-4^, (for example Thon and Friis, 1997; Grewal and Klar, 1998). The continuous growth in non-selective media of OFF cells over > 25 generations resulted in the appearance 1-2% ON colonies, consistent with these low transitions (Grewal and Klar, 1996). A key difference between our and some previous experiments is observing the entire population without selection (our assays) versus selection assays that permit survival of either the ON or OFF state. Thus, the appearance of very small ON populations may be less obvious in our experiments.

The memory experiments in Figure 4 reveal the key contribution of *REIII* to resistance and memory of MAT heterochromatin. The comparison of wild-type to ∆*REIII* cells here is central and valid in our view. However, we recognize that in Figure 4D, the results on its face point to stronger memory in ∆K cells than wild type. The “extreme” aspect of this memory is most likely due to the fact that ∆K cells, once ancestrally TSA treated, cannot efficiently re-nucleate. Given this caveat, we now refrain from interpreting Figure 4D as indicative of the degree of memory in ∆K. Overall, we did not intend to advance the notion that ∆K cells have more/stronger memory than wild-type and wherever this is ambiguous, have clarified the language. We note that unlike for ∆K cells, it is not possible to select for WT cells with active *REIII,* thus the population may contain some cells with *REIII* in an inactive state, which likely are more labile than the bulk of cells with active *REIII*. Hence, ∆K_OFF_ populations can appear somewhat more stable as a result.

As to ∆K_OFF_ selection, our approach is consistent with the literature (Grewal and Klar, 1996 and 1998; Jia et al., 2004; Thon and Friis, 1997 and others). We produce ∆K^HSS^ strains in a ∆*clr4* background and introduce *clr4+* by cross. The population resulting from the germinated spore is often mixed, with both ON and OFF cells as shown in Figure 2E. Simply plating this population on nonselective media predominantly yields colonies that are ON or OFF. This reflects the metastable nature of ∆K cells, with some cells nucleating heterochromatin and some not, but then maintaining the state. In that sense, we do not isolate ∆K_OFF_ cells using a directed selection process, promoting a given state, and do not feel that there should be any bias introduced by this method. Isolation of ON/OFF is required in some cases, for example, due to the insufficient number of OFF cells in the mixed population. However, we now qualify our results pertaining to memory clearly and state that bias may have been introduced by picking OFF colonies.

In general, as suggested, we re-focused our conclusions on ∆*REIII*. Together with our reinterpretation of the ∆K result in Figure 4, we believe we addressed remaining concerns.

My recommendation is to prepare a revised manuscript for publication in eLife that focuses on the results from their elegant single cell assays showing that the loss of REIII affects the stability of heterochromatin and that this behavior of REIII is mechanistically linked to the suppression of histone turnover at mat. The discussion would be focused on succinctly describing this feature of REIII in the context of previous findings, including the results showing that REIII recruits the HDAC Clr3 (Yamada et al., 2005) that also suppresses histone turnover to promote epigenetic stability of heterochromatin (Aygun et al., 2013). Indeed, suppression of histone turnover by REIII /HDAC Clr3 may promote retention of methylated histones that are critical for the recruitment of Clr4; thus, promoting the efficient spreading of heterochromatin. Framing the results in this way would avoid the problematic issues raised by applying the terms "memory element" or "deterministic" spreading to REIII.

We thank the reviewer for these suggestions. Indeed, we have re-written the manuscript, most prominently the Abstract and Discussion section, to focus on the overall result that ncRNA spreading is unstable and stabilized by *REIII* via suppression of histone turnover. We no longer use the terms “deterministic” or “memory element” with respect to *REIII.*

Reviewer #3: The authors have addressed all the critiques raised by this reviewer. In particular, they have now provided more compelling evidence of "spreading" of heterochromatin from corresponding nucleation sites with a more complete testing cassette of different distances, directions, nucleation factors, etc. They have used these testing cassettes to demonstrate the stochasticity of ncRNA-mediated spreading and stability of REIII -mediated memory. In addition, they have now tested the anti-correlation of histone turnover and memory.

We thank the reviewer for this positive assessment.
